# Supplementary material for: A Chiral Relay Race: Stereoselective Synthesis of Axially Chiral Biaryl Diketones through Ring-Opening of Optical Dihydrophenan-threne-9,10-diols
Source: Molecules. 2023 Aug 8;28(16):5956. doi: 10.3390/molecules28165956 (PMC10459955; doi:10.3390/molecules28165956)
Supplement: Supplementary file 1 [file molecules-28-05956-s001.zip › molecules-2505306-supplementary.pdf]

A Chiral Relay Race: Stereoselective Synthesis of Axially Chiral Biaryl Diketones through Ring-Opening of Optical Dihydrophenanthrene-9,10-diols

Lei Shi,[1] Jiawei Zhu,[1] Biqiong Hong[2] and Zhenhua Gu*[1,2]

1. Hefei National Research Center for Physical Sciences at the Microscale and Department of Chemistry, University of Science and Technology of China, 96 Jinzhai Road, Hefei, Anhui 230026, P. R. China
2. College of Materials and Chemical Engineering, Minjiang University, Fuzhou, Fujian, 350108, China
Email: zhgu@ustc.edu.cn

Free radical capture experiment

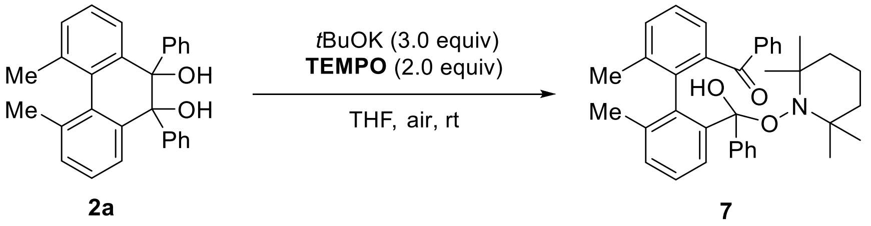

Under air atmosphere, a solution of **2a** (39.2 mg, 0.10 mmol, 1.0 equiv) in THF (2 mL) was added *t*BuOK (33.6 mg, 0.30 mmol, 3.0 equiv) and **TEMPO** (31.3 mg, 0.20 mmol, 2.0 equiv) at room temperature, then the mixture was stirred at the same temperature for 30 min. 0.5 mL of the reaction mixture was taken out and it was passed through a short pad of silica gel. **7** was detected by the analysis of HRMS. HRMS (ESI) calcd for C37H42NO3 [M+H]+ 548.3159, found 548.3168. HRMS (ESI) calcd for 37H41NO3Na [M+Na]+ 570.2979, found 570.2992.

Copies of NMR Spectroscopies

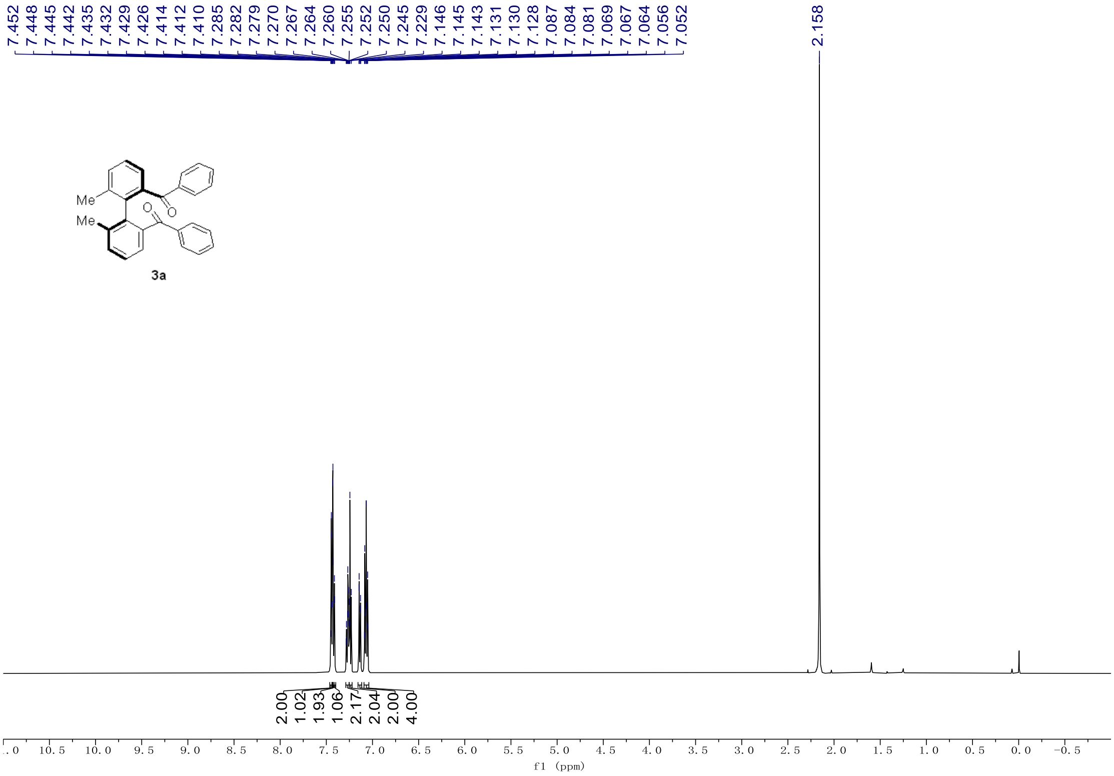

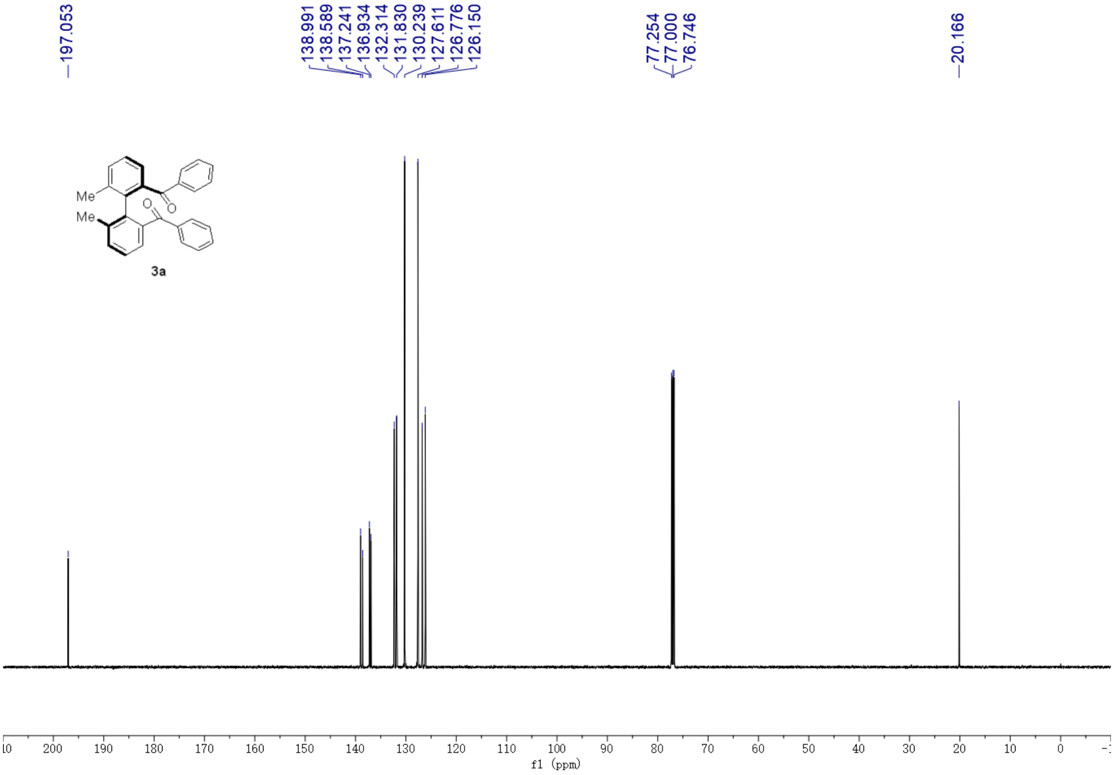

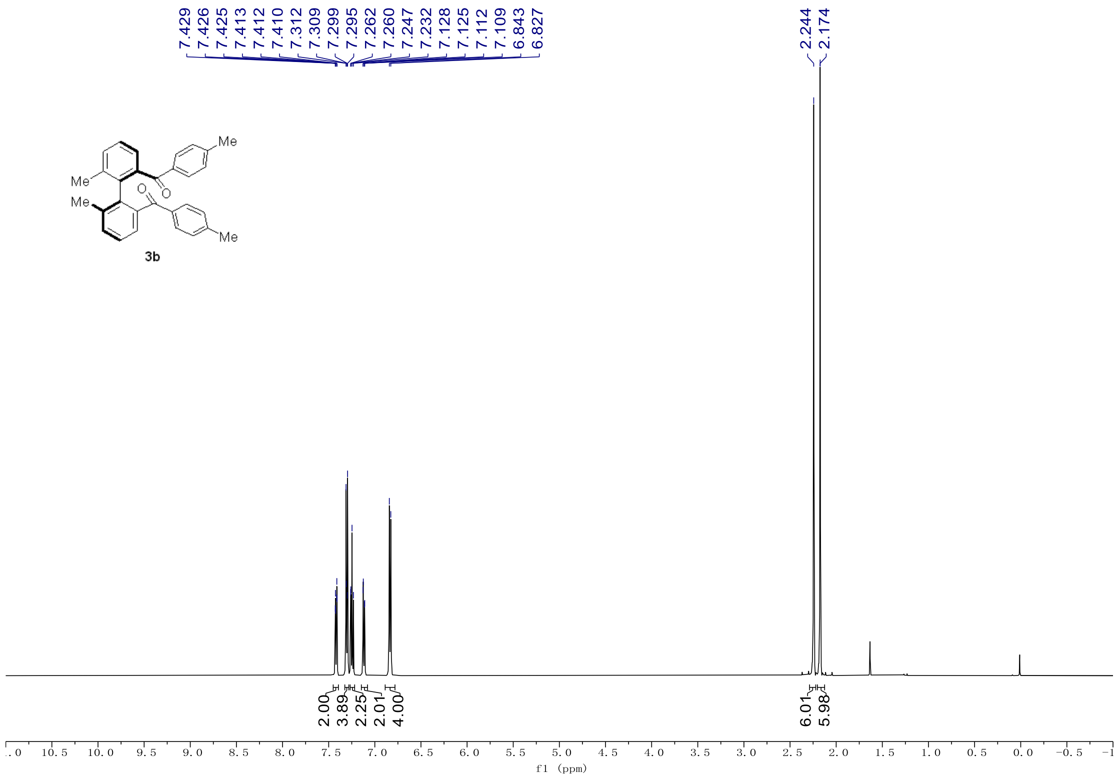
7.429
7.426
7.425
7.413
7.412
7.410
7.312
7.309
7.299
7.295
7.262
7.260
7.247
7.232
7.128
7.125
7.112
7.109
6.843
6.827
2.244
2.174
Me
O
Me
O
Me
Me
3b
2.00
3.89
2.25
2.01
4.00
6.01
5.98
f1 (ppm)

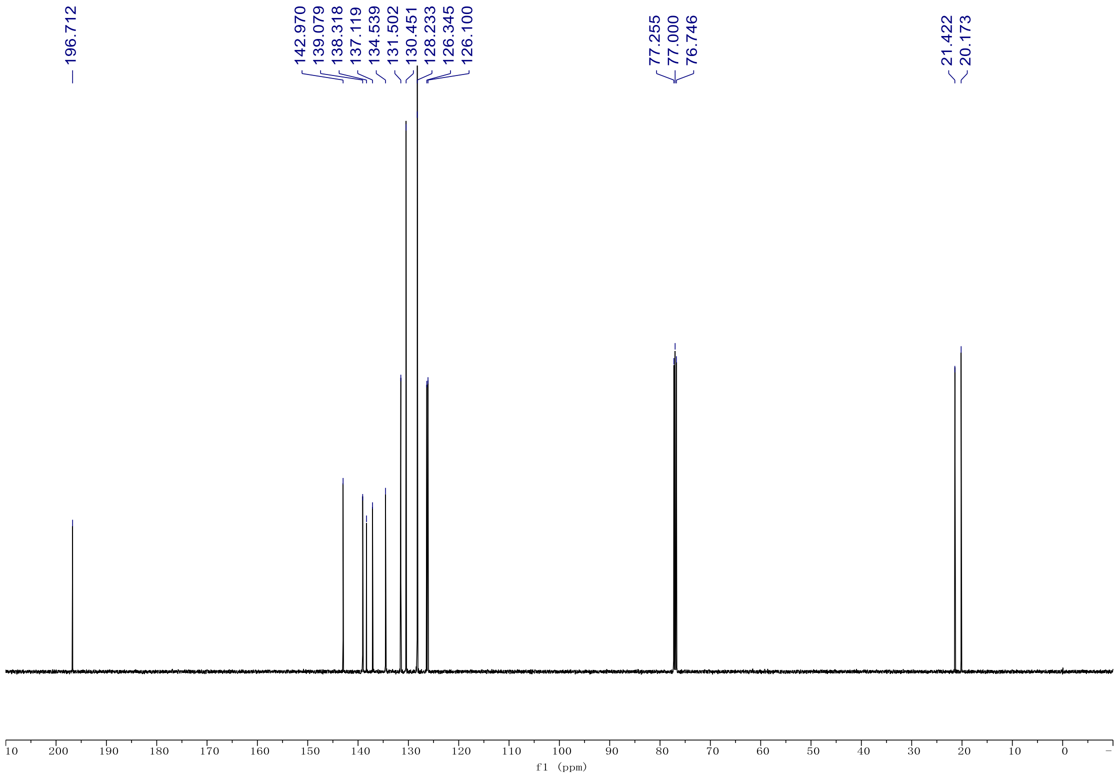
196.712
142.970
139.079
138.318
137.119
134.539
131.502
130.451
128.233
126.345
126.100
77.255
77.000
76.746
21.422
20.173
f1 (ppm)

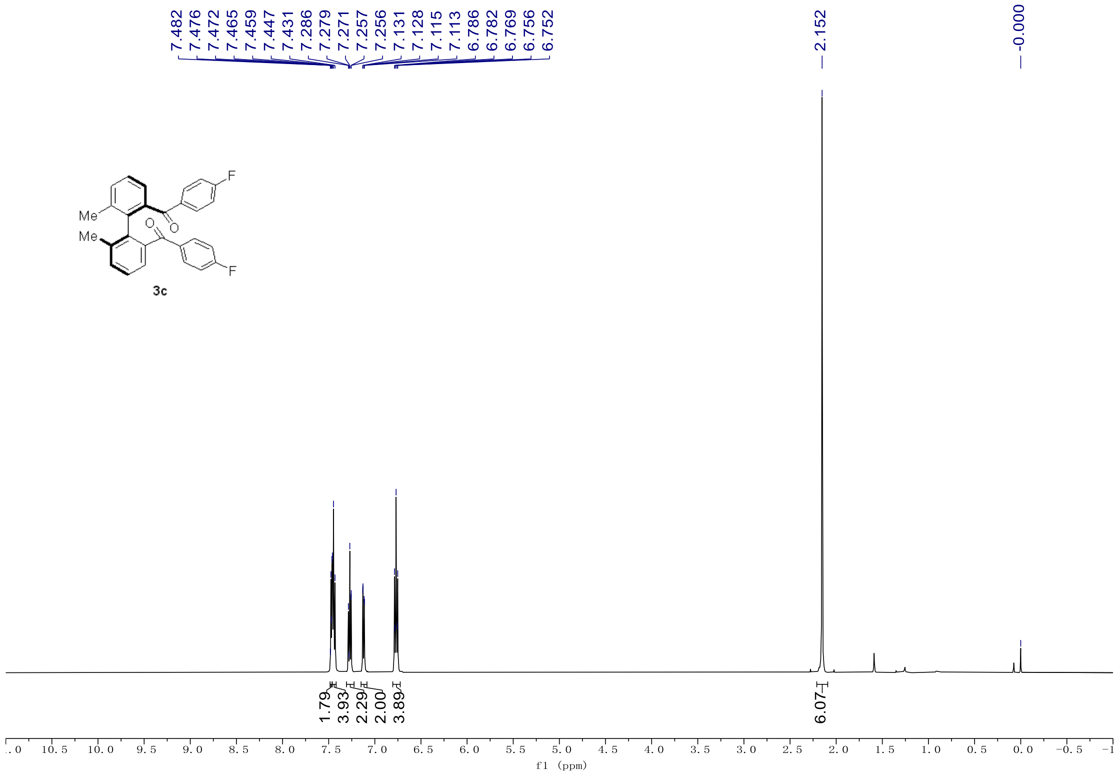
7.482
7.476
7.472
7.465
7.459
7.447
7.431
7.286
7.279
7.271
7.257
7.256
7.131
7.128
7.115
7.113
6.786
6.782
6.769
6.756
6.752
2.152
-0.000
Me
Me
O
O
F
F
3c
1.79
3.93
2.29
2.00
3.89
6.07
f1 (ppm)

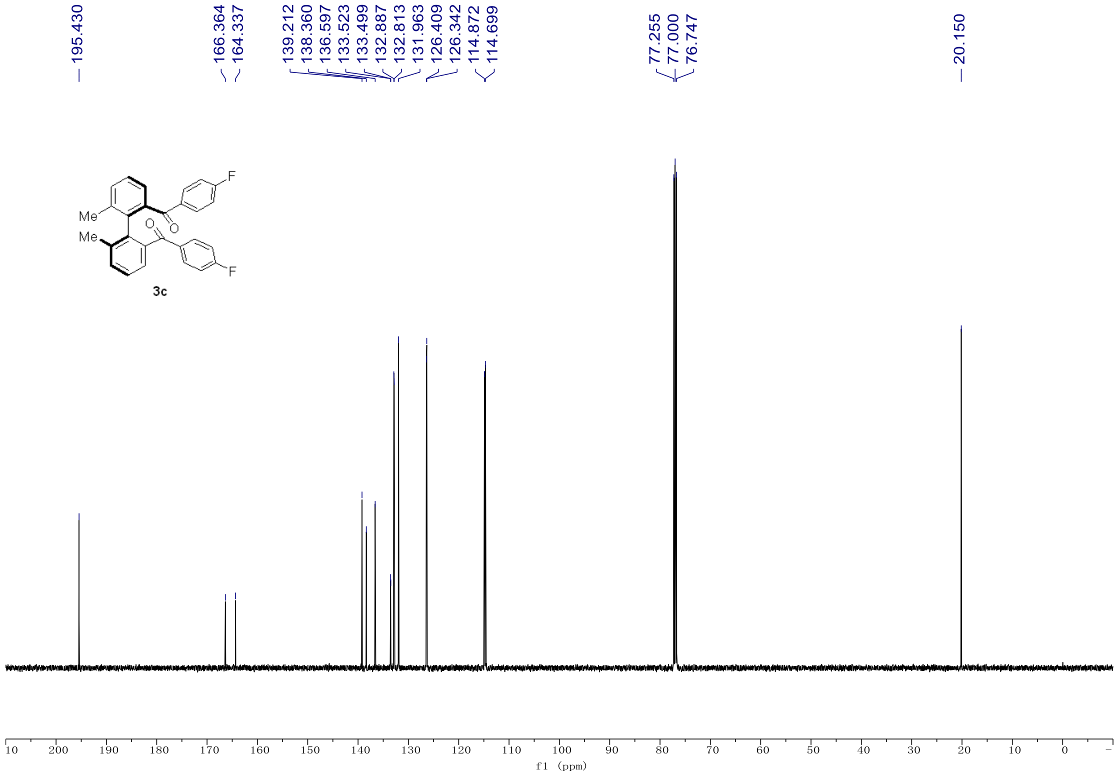
195.430
166.364
164.337
139.212
138.360
136.597
133.523
133.499
132.887
132.813
131.963
126.409
126.342
114.872
114.699
77.255
77.000
76.747
20.150
Me
Me
O
O
F
F
3c
f1 (ppm)

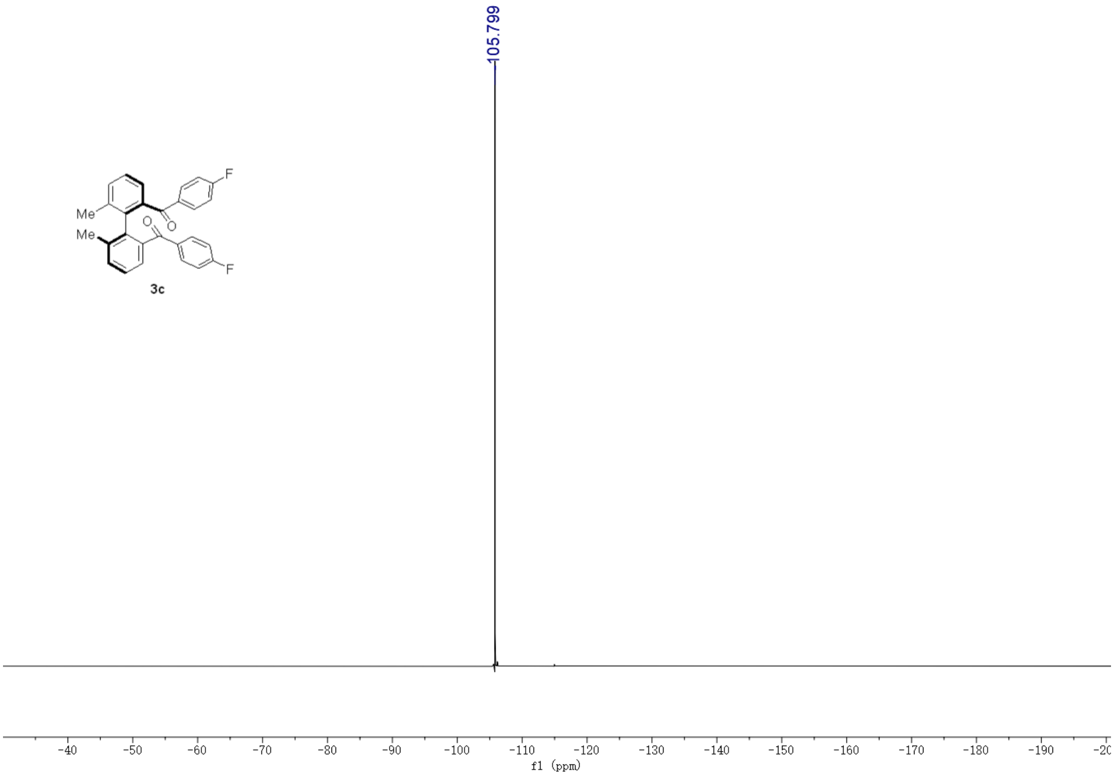
105.799
Me
Me
O
O
F
F
3c
f1 (ppm)

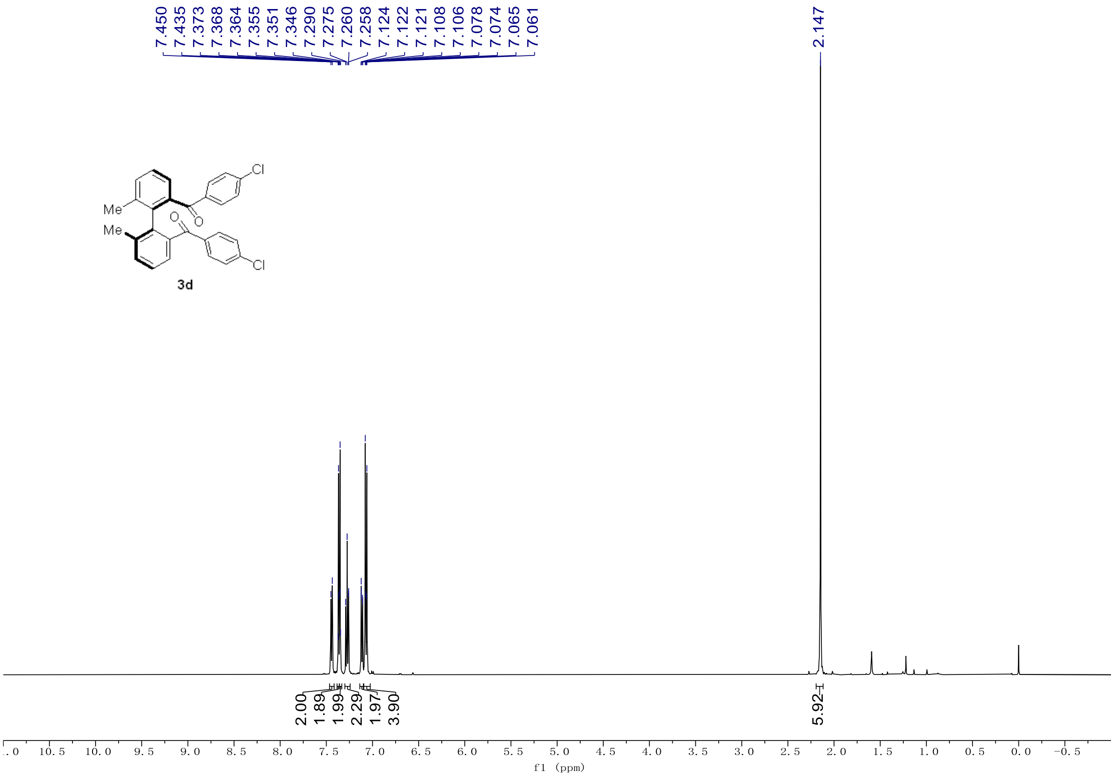
7.450
7.435
7.373
7.368
7.364
7.355
7.351
7.346
7.290
7.275
7.260
7.258
7.124
7.122
7.121
7.108
7.106
7.078
7.074
7.065
7.061
2.147
Me
Me
O
O
Cl
Cl
3d
2.00
1.89
1.99
2.29
1.97
3.90
5.92
f1 (ppm)

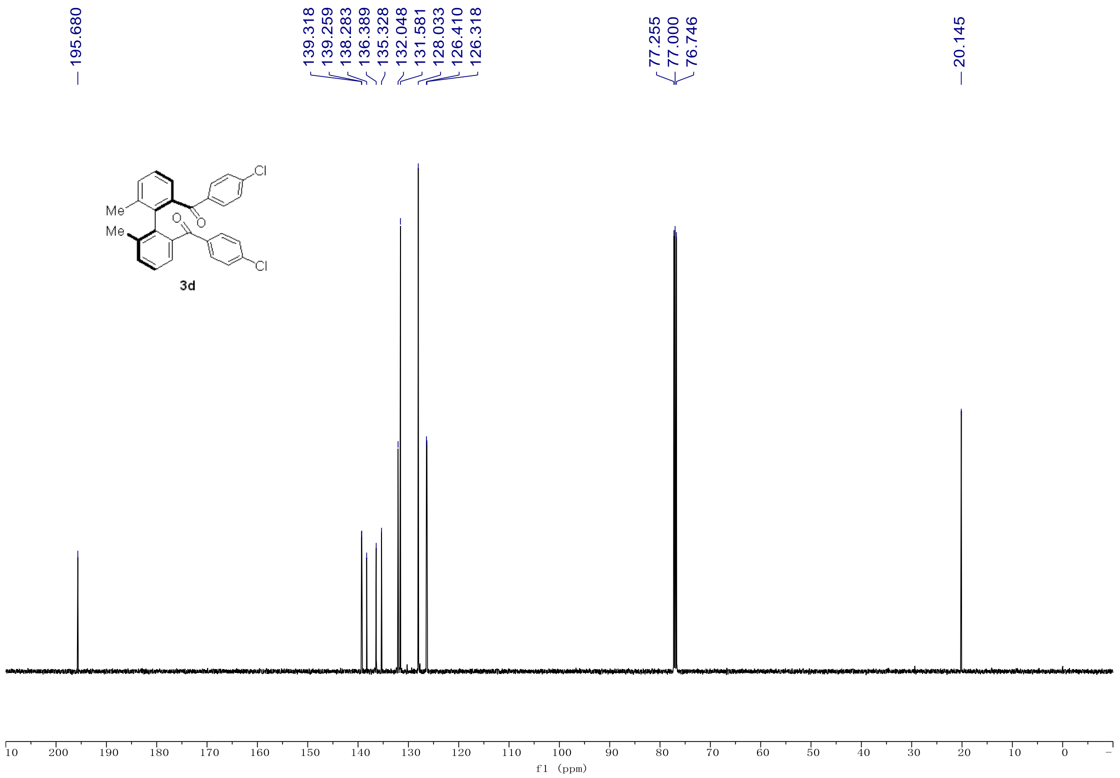
195.680
139.318
139.259
138.283
136.389
135.328
132.048
131.581
128.033
126.410
126.318
77.255
77.000
76.746
20.145
Me
Me
O
O
Cl
Cl
3d
f1 (ppm)

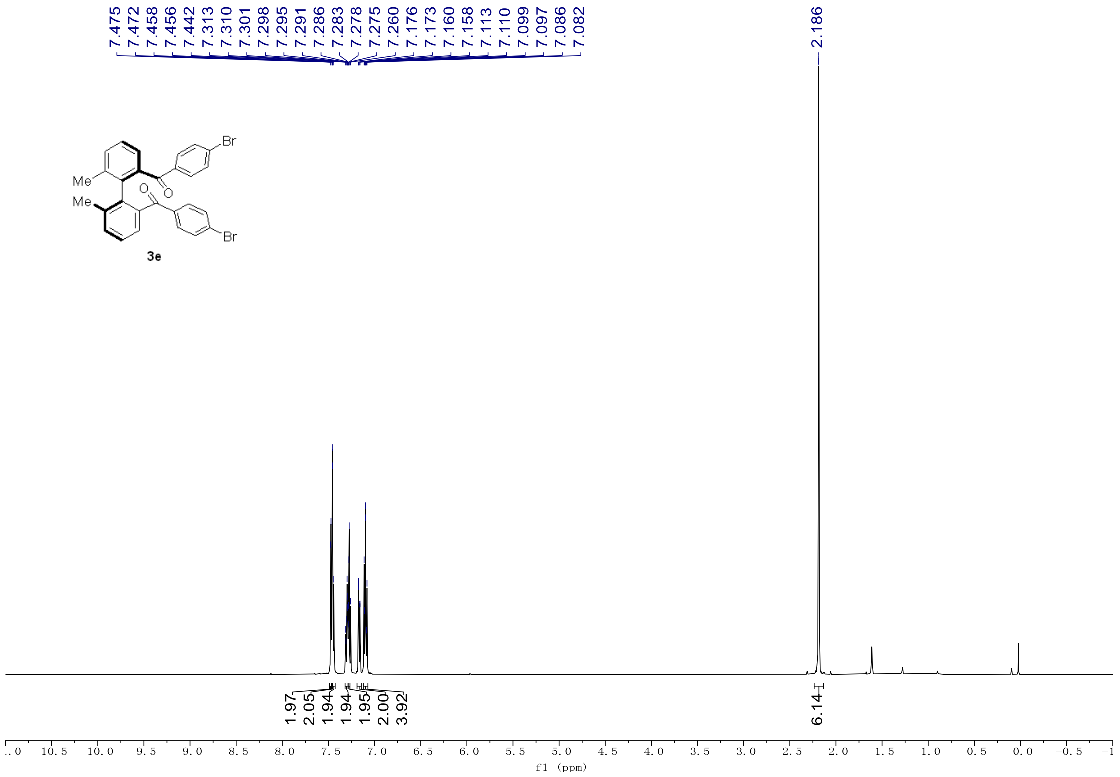
7.475
7.472
7.458
7.456
7.442
7.313
7.310
7.301
7.298
7.295
7.291
7.286
7.283
7.278
7.275
7.260
7.176
7.173
7.160
7.158
7.113
7.110
7.099
7.097
7.086
7.082
2.186
Me
Me
O
O
Br
Br
3e
1.97
2.05
1.94
1.94
1.95
2.00
3.92
6.14
f1 (ppm)

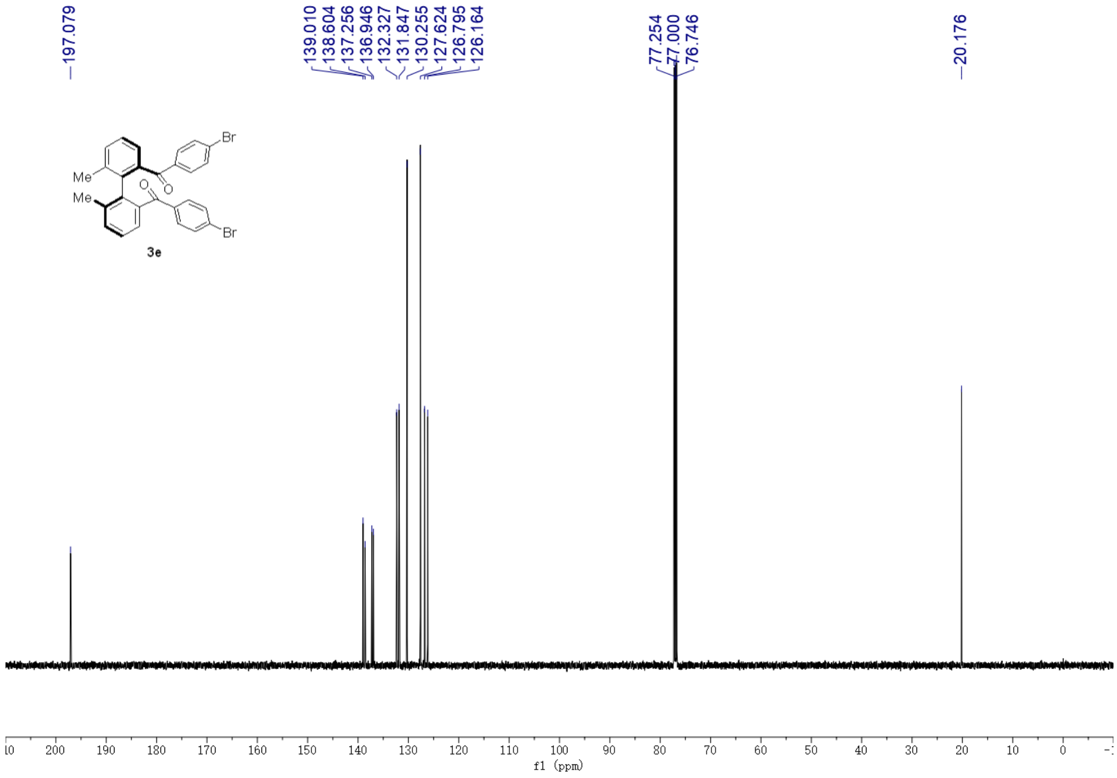
197.079
139.010
138.604
137.256
136.946
132.327
131.847
130.255
127.624
126.795
126.164
77.254
77.000
76.746
20.176
Br
Me
Me
O
O
Br
3e
f1 (ppm)

SL-5-73.1.fid

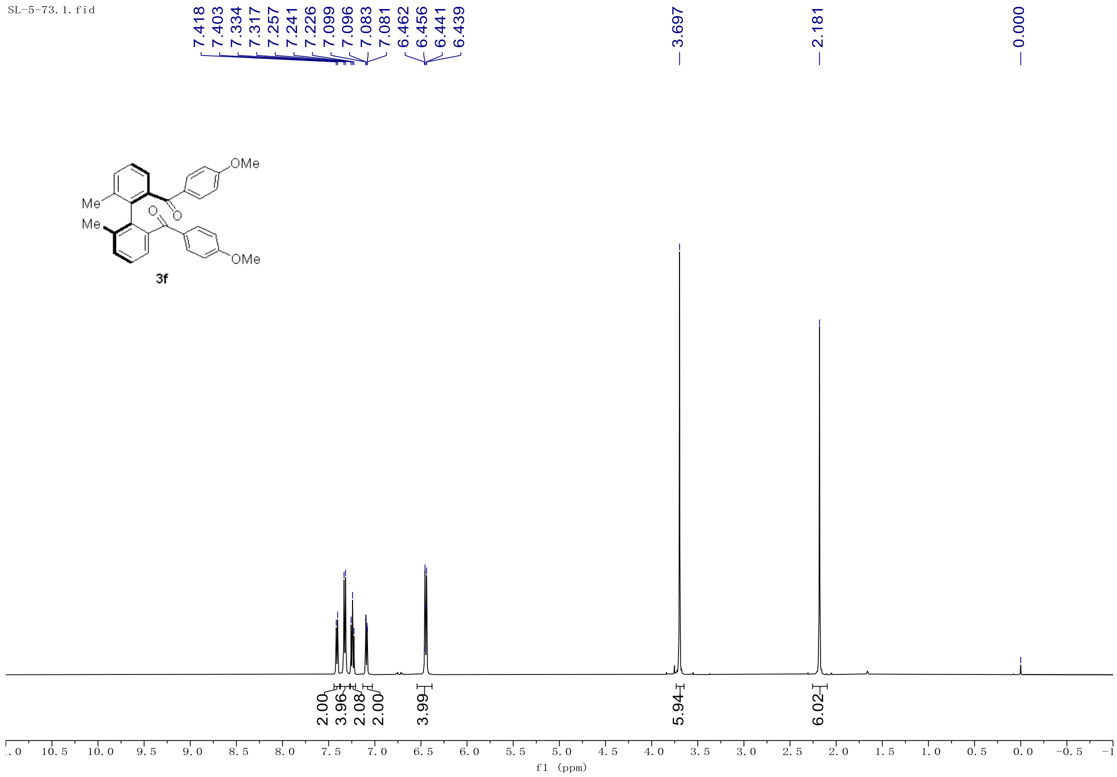
7.418
7.403
7.334
7.317
7.257
7.241
7.226
7.099
7.096
7.083
7.081
6.462
6.456
6.441
6.439
3.697
2.181
0.000
OMe
Me
Me
O
O
OMe
3f
2.00
3.96
2.08
2.00
3.99
5.94
6.02
f1 (ppm)

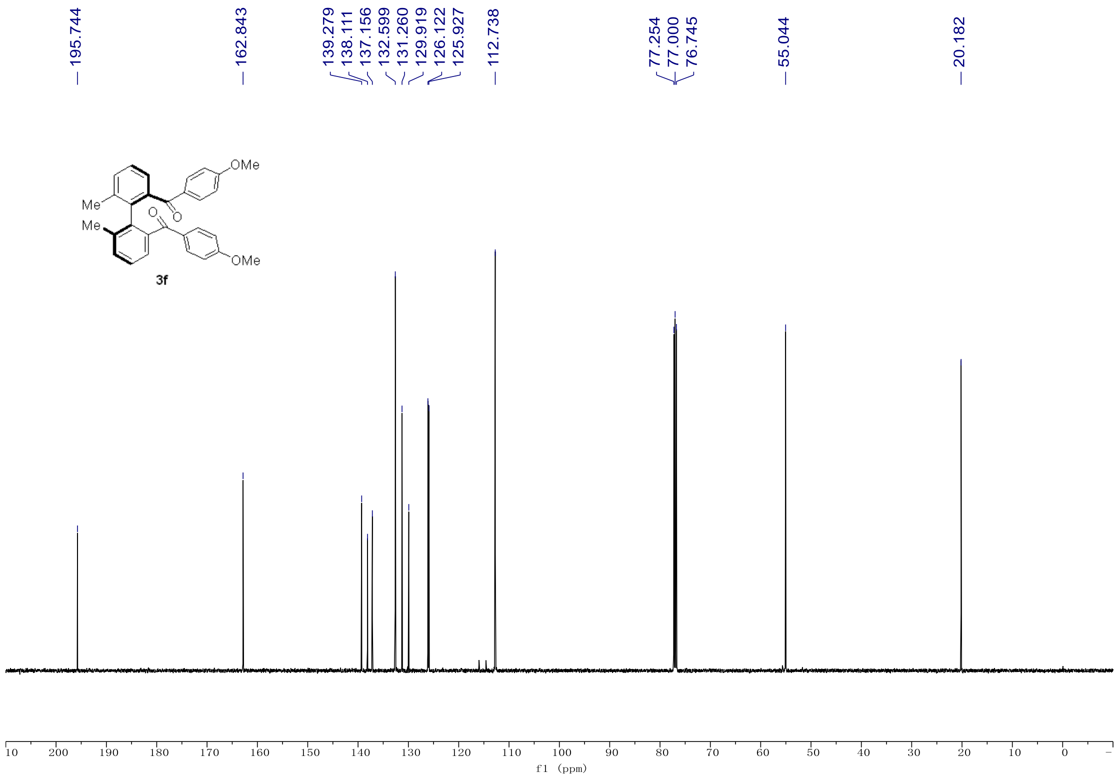
195.744
162.843
139.279
138.111
137.156
132.599
131.260
129.919
126.122
125.927
112.738
77.254
77.000
76.745
55.044
20.182
OMe
Me
Me
O
O
OMe
3f
f1 (ppm)

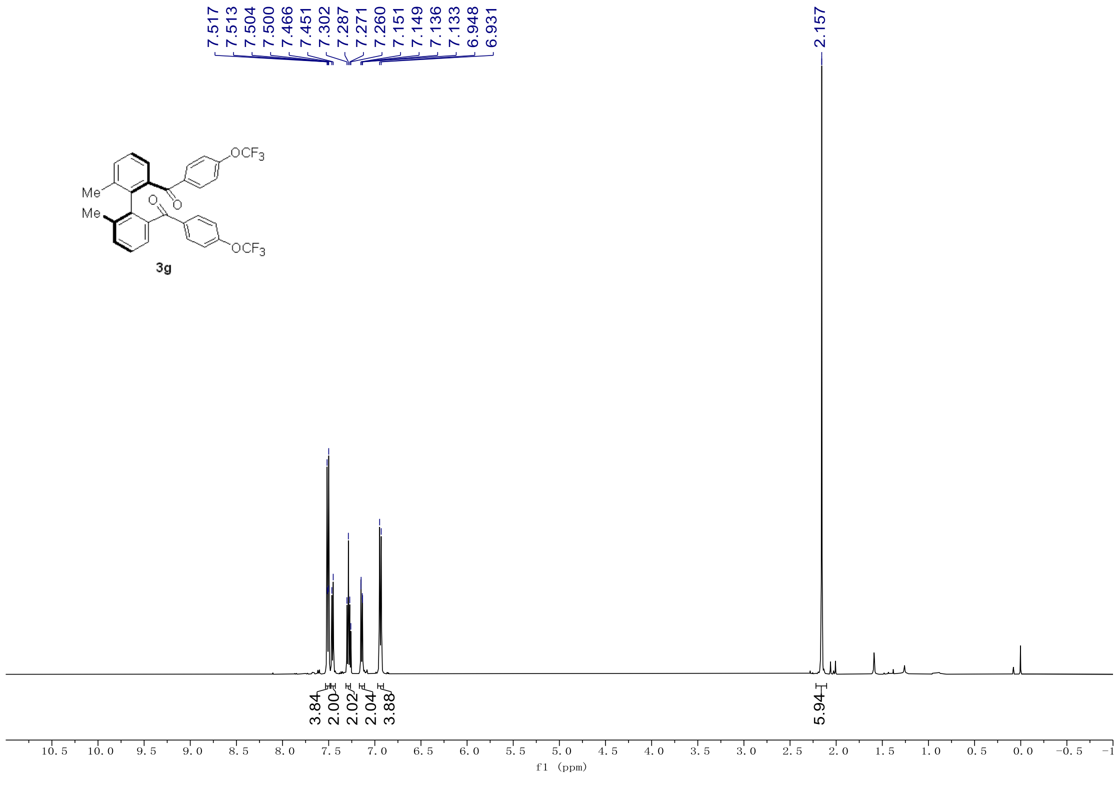
7.517
7.513
7.504
7.500
7.466
7.451
7.302
7.287
7.271
7.260
7.151
7.149
7.136
7.133
6.948
6.931
2.157
OCF3
Me
Me
O
O
OCF3
3g
3.84
2.00
2.02
2.04
3.88
5.94
f1 (ppm)

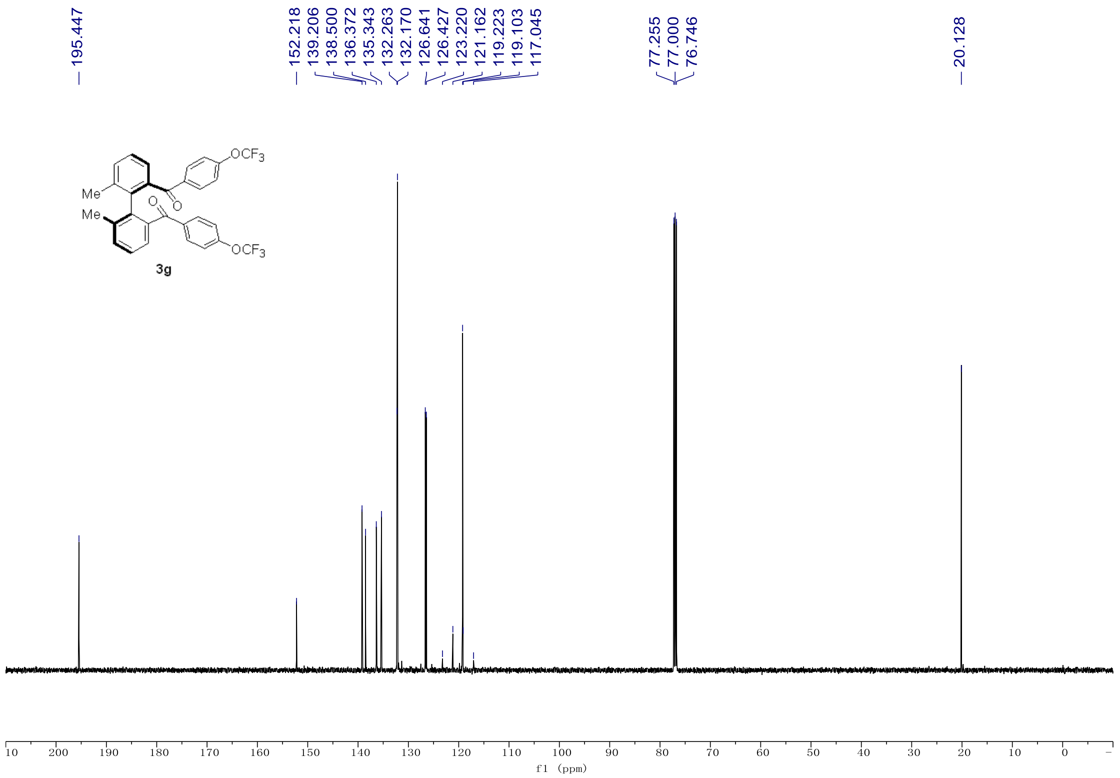
195.447
152.218
139.206
138.500
136.372
135.343
132.263
132.170
126.641
126.427
123.220
121.162
119.223
119.103
117.045
77.255
77.000
76.746
20.128
OCF3
Me
Me
O
O
OCF3
3g
f1 (ppm)

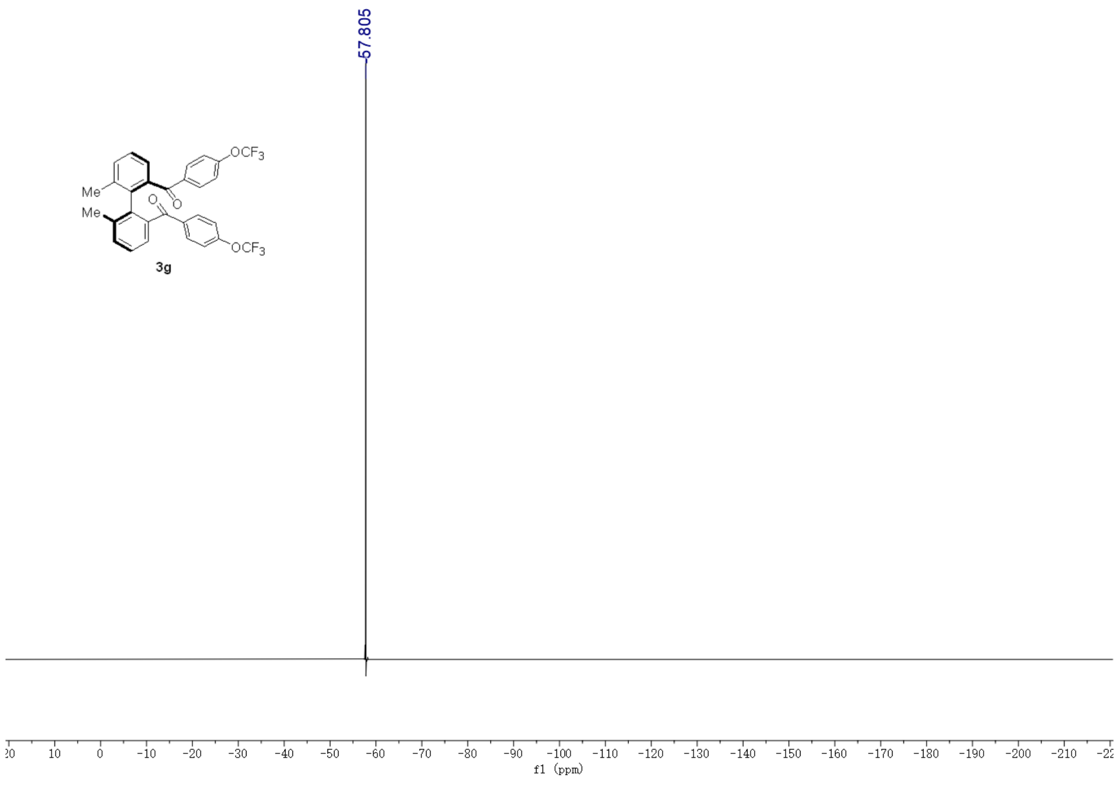
-57.805
OCF3
Me
Me
O
O
OCF3
3g
f1 (ppm)

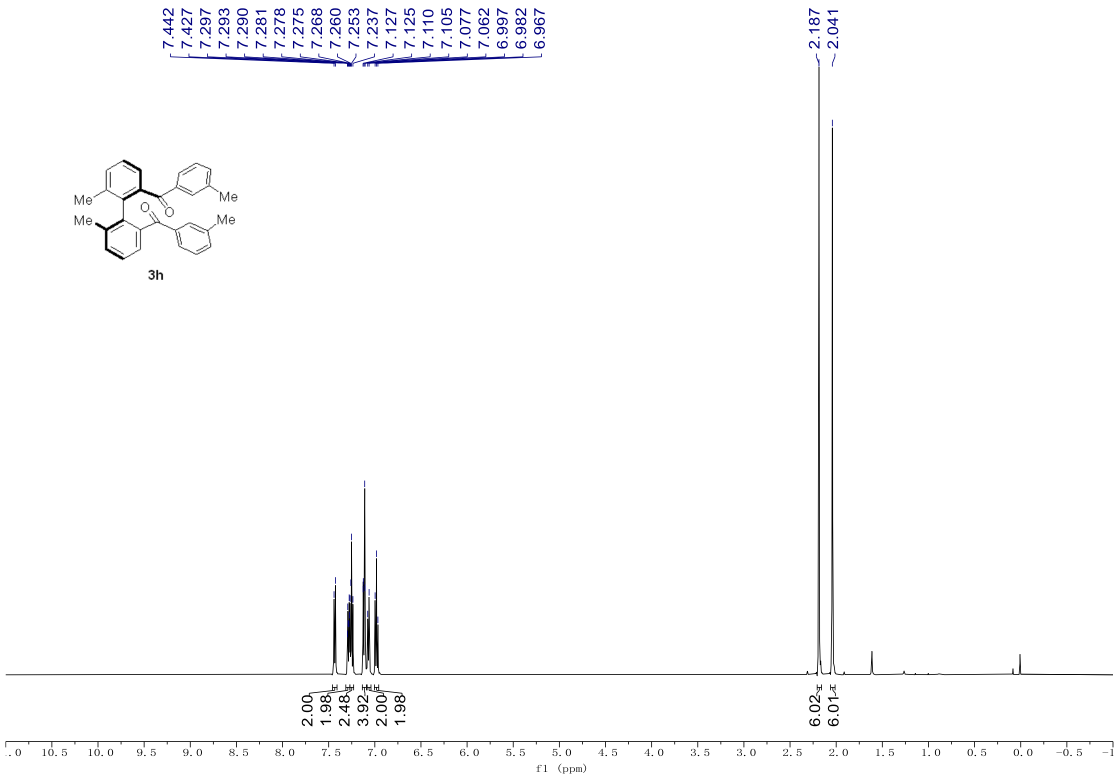
7.442
7.427
7.297
7.293
7.290
7.281
7.278
7.275
7.268
7.260
7.253
7.237
7.127
7.125
7.110
7.105
7.077
7.062
6.997
6.982
6.967
2.187
2.041
Me
Me
O
O
Me
Me
3h
2.00
1.98
2.48
3.92
2.00
1.98
6.02
6.01
f1 (ppm)

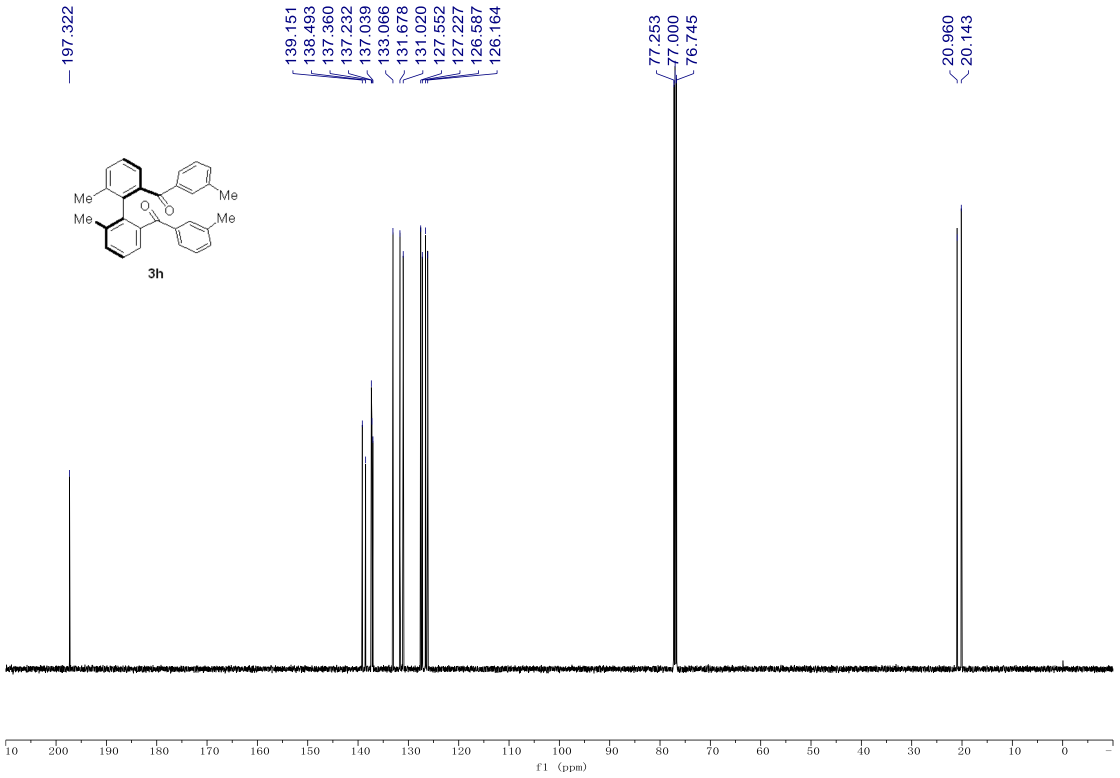
197.322
139.151
138.493
137.360
137.232
137.039
133.066
131.678
131.020
127.552
127.227
126.587
126.164
77.253
77.000
76.745
20.960
20.143
Me
Me
O
O
Me
Me
3h
f1 (ppm)

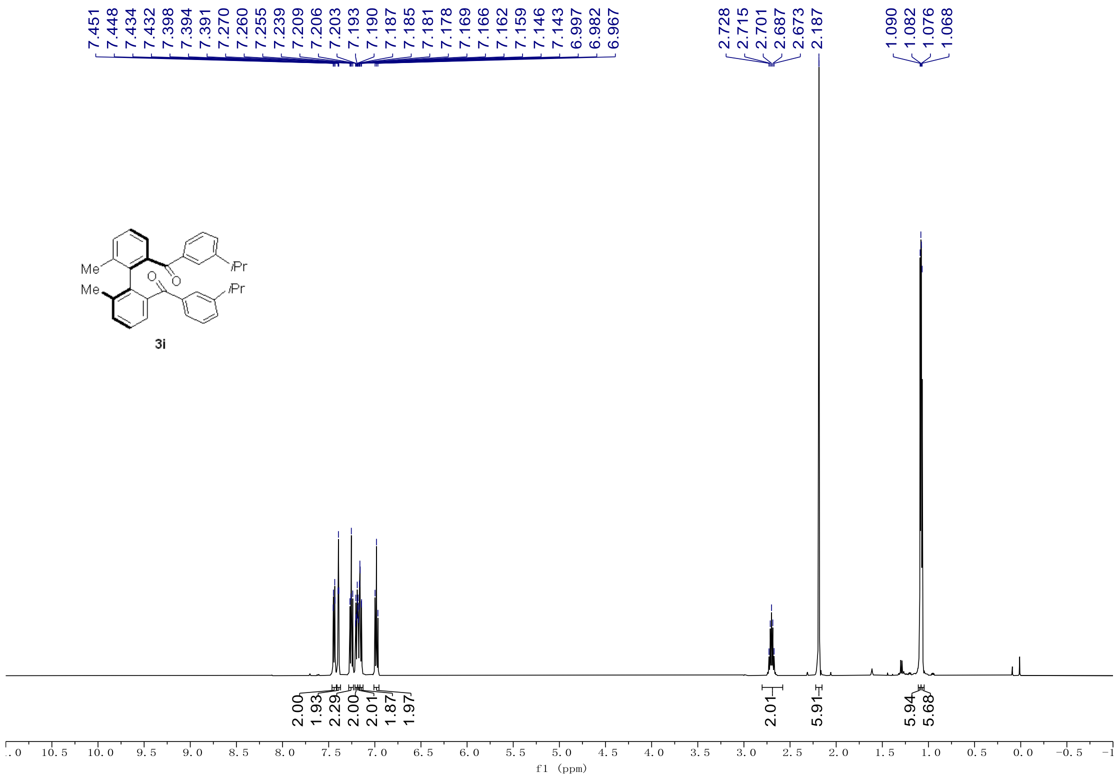

7.451 7.448 7.434 7.432 7.398 7.394 7.391 7.270 7.260 7.255 7.239 7.209 7.206 7.203 7.193 7.190 7.187 7.185 7.181 7.178 7.169 7.166 7.162 7.159 7.146 7.143 6.997 6.982 6.967
2.728 2.715 2.701 2.687 2.673 2.187
1.090 1.082 1.076 1.068
Me
Me
O
O
iPr
iPr
3i
2.00 1.93 2.29 2.00 2.01 1.87 1.97
2.01
5.91
5.94 5.68
f1 (ppm)

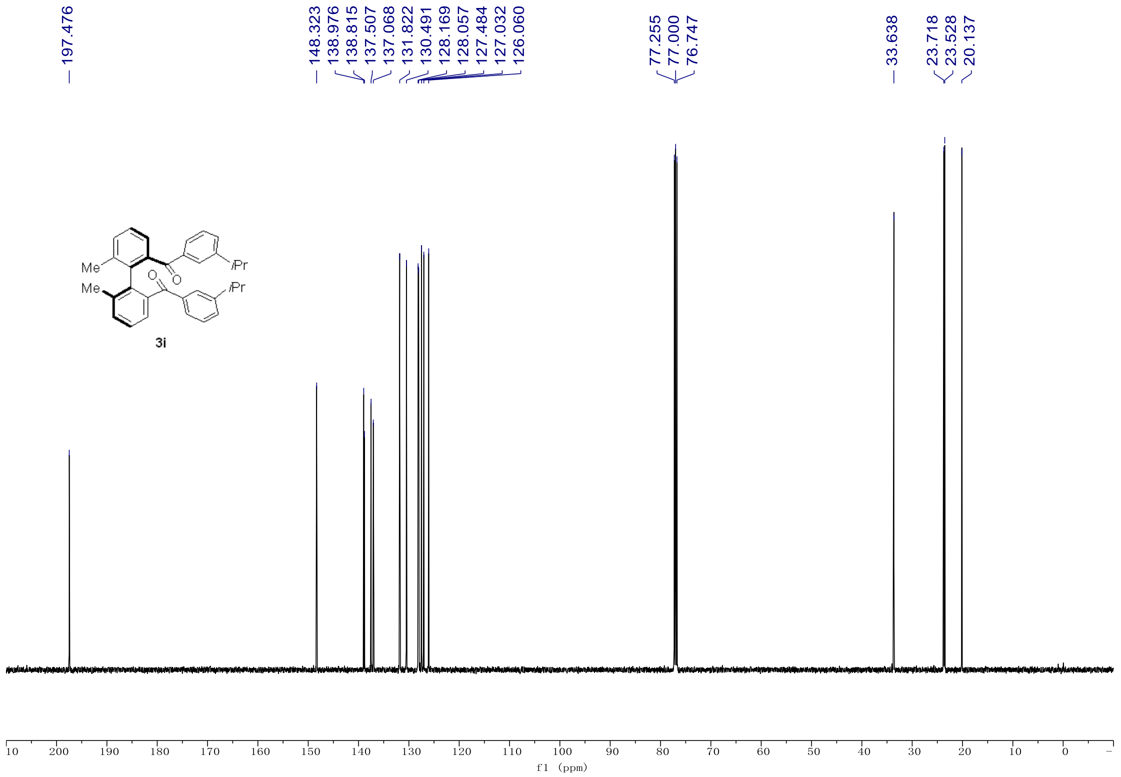

197.476
148.323 138.976 138.815 137.507 137.068 131.822 130.491 128.169 128.057 127.484 127.032 126.060
77.255 77.000 76.747
33.638
23.718 23.528 20.137
Me
Me
O
O
iPr
iPr
3i
f1 (ppm)

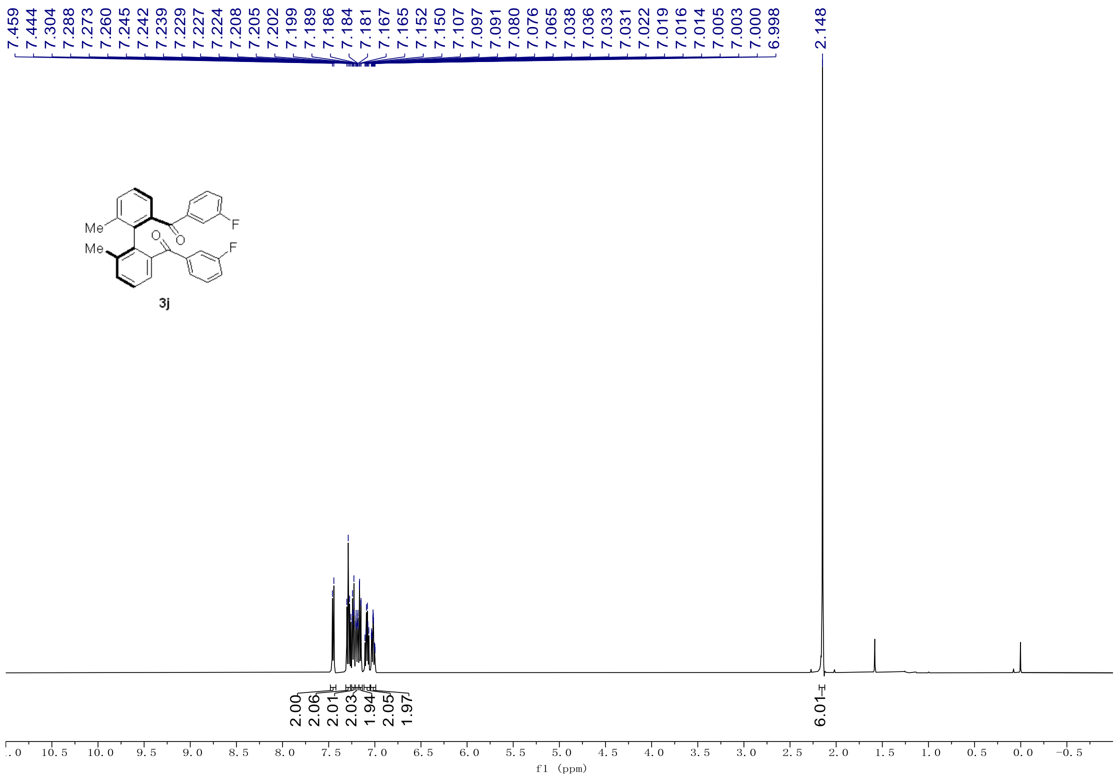
7.459 7.444 7.304 7.288 7.273 7.260 7.245 7.242 7.239 7.229 7.227 7.224 7.208 7.205 7.202 7.199 7.189 7.186 7.184 7.181 7.167 7.165 7.152 7.150 7.107 7.097 7.091 7.080 7.076 7.065 7.038 7.036 7.033 7.031 7.022 7.019 7.016 7.014 7.005 7.003 7.000 6.998
2.148
Me
Me
O
O
F
F
3j
2.00 2.06 2.01 2.03 1.94 2.05 1.97
6.01
f1 (ppm)

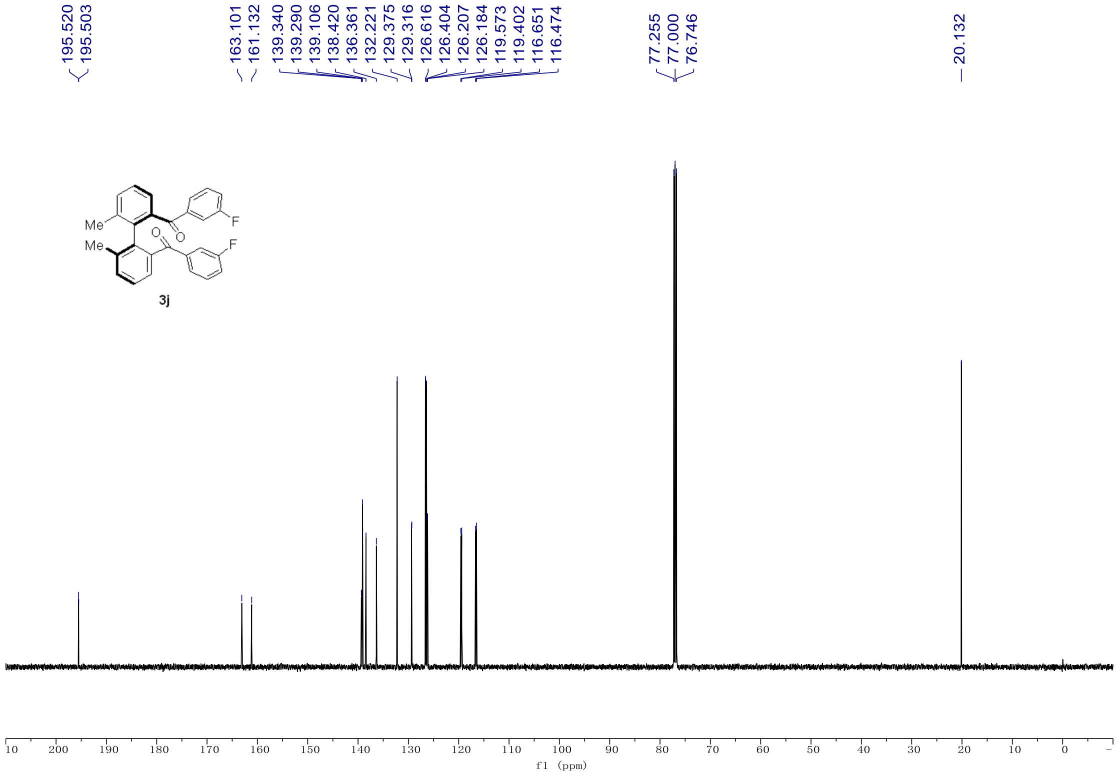
195.520 195.503
163.101 161.132
139.340 139.290 139.106 138.420 136.361 132.221 129.375 129.316 126.616 126.404 126.207 126.184 119.573 119.402 116.651 116.474
77.255 77.000 76.746
20.132
Me
Me
O
O
F
F
3j
f1 (ppm)

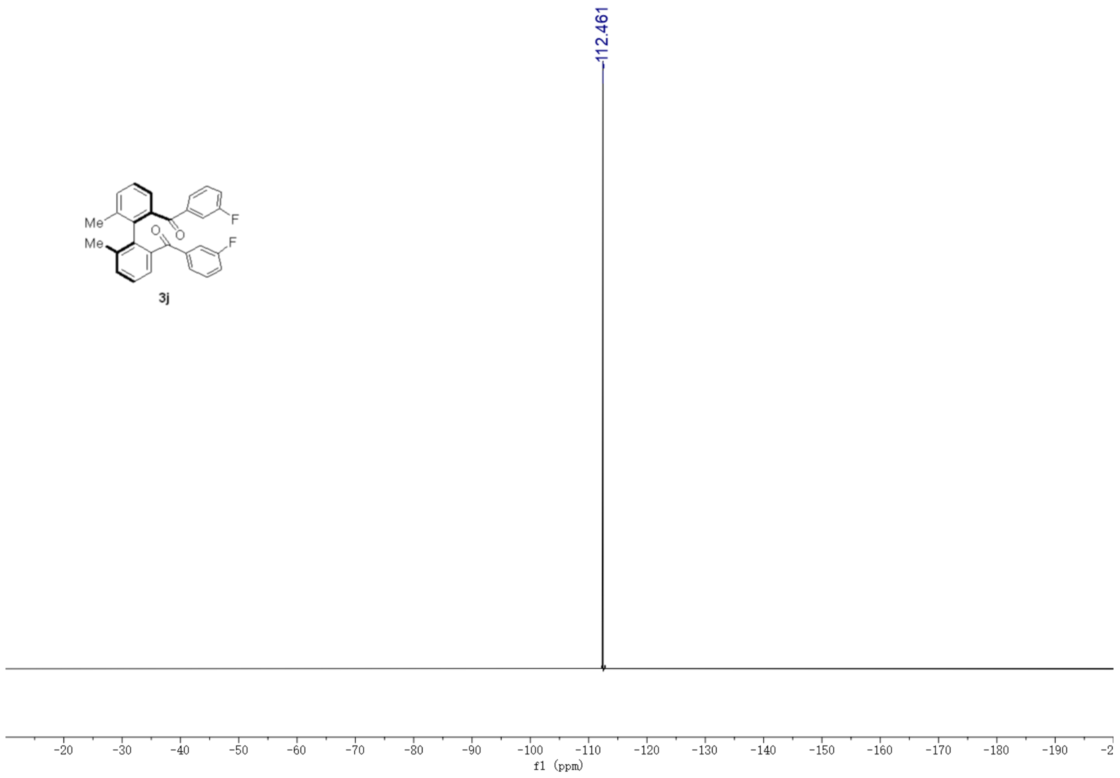

-112.461
Me
Me
O
O
F
F
3j
f1 (ppm)

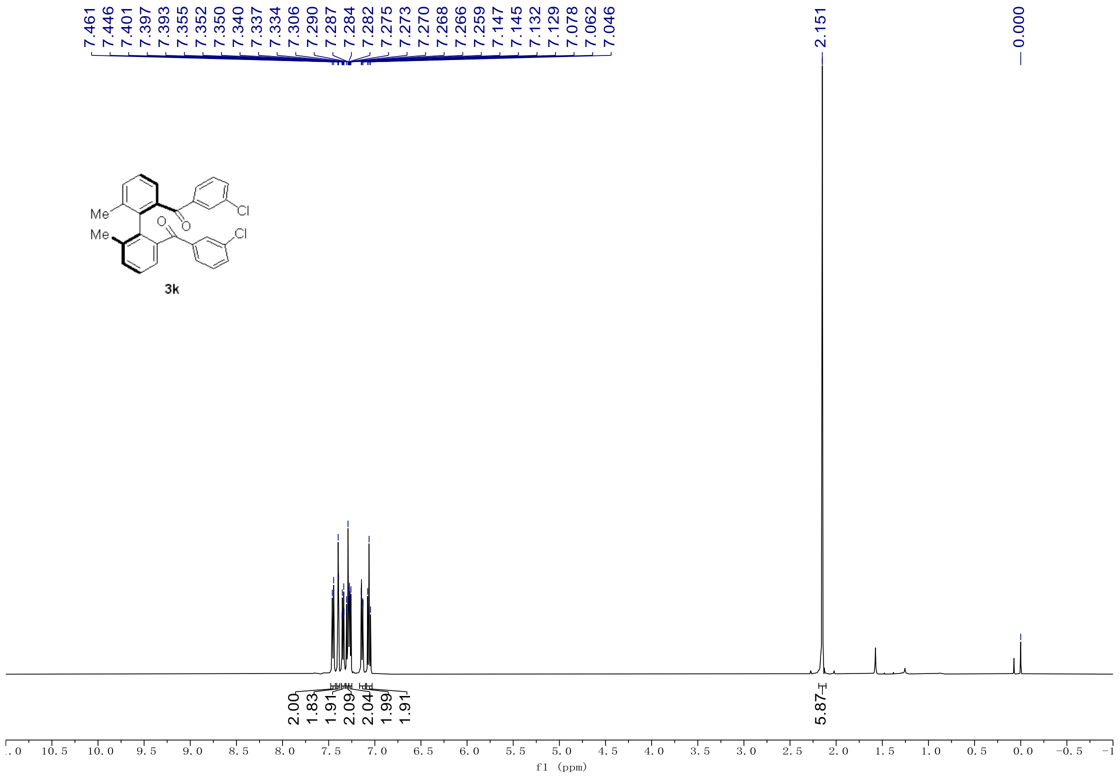

7.461
7.446
7.401
7.397
7.393
7.355
7.352
7.350
7.340
7.337
7.334
7.306
7.290
7.287
7.284
7.282
7.275
7.273
7.270
7.268
7.266
7.259
7.147
7.145
7.132
7.129
7.078
7.062
7.046
2.151
0.000
Me
Me
O
O
Cl
Cl
3k
2.00
1.83
1.91
2.09
2.04
1.99
1.91
5.87
f1 (ppm)

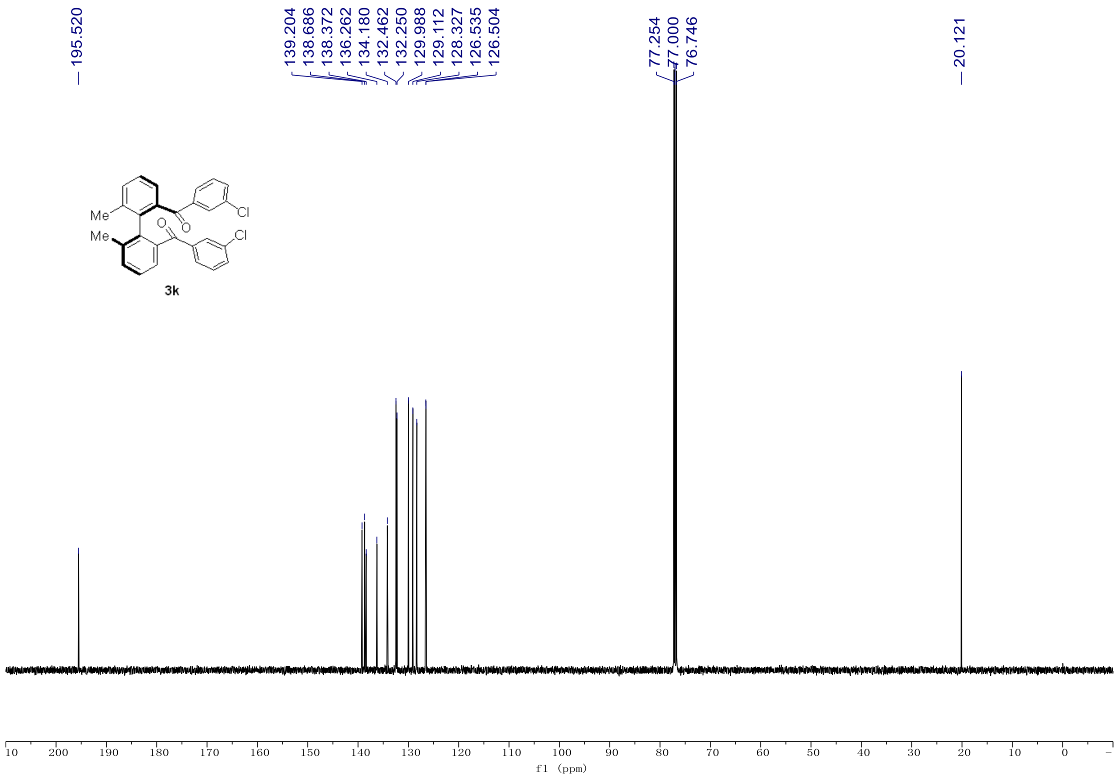

195.520
139.204
138.686
138.372
136.262
134.180
132.462
132.250
129.988
129.112
128.327
126.535
126.504
77.254
77.000
76.746
20.121
Me
Me
Cl
Cl
O
O
3k
f1 (ppm)

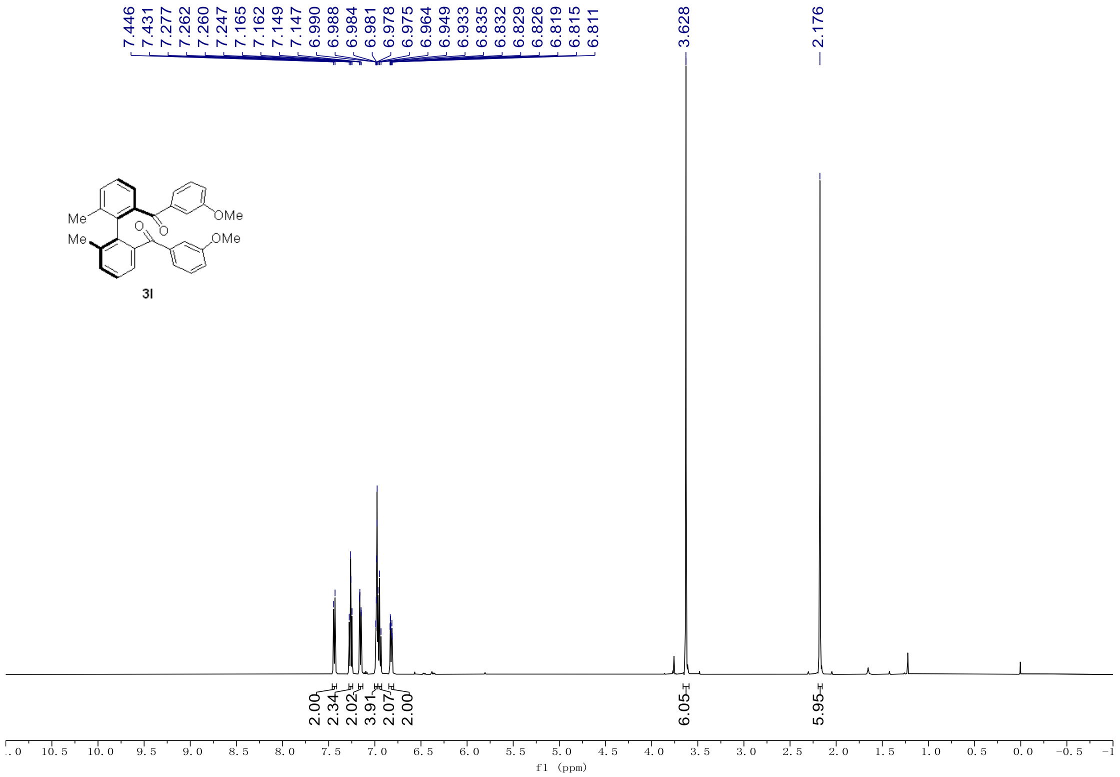

7.446
7.431
7.277
7.262
7.260
7.247
7.165
7.162
7.149
7.147
6.990
6.988
6.984
6.981
6.978
6.975
6.964
6.949
6.933
6.835
6.832
6.829
6.826
6.819
6.815
6.811
3.628
2.176
Me
Me
OMe
OMe
O
O
3l
2.00
2.34
2.02
3.91
2.07
2.00
6.05
5.95
f1 (ppm)

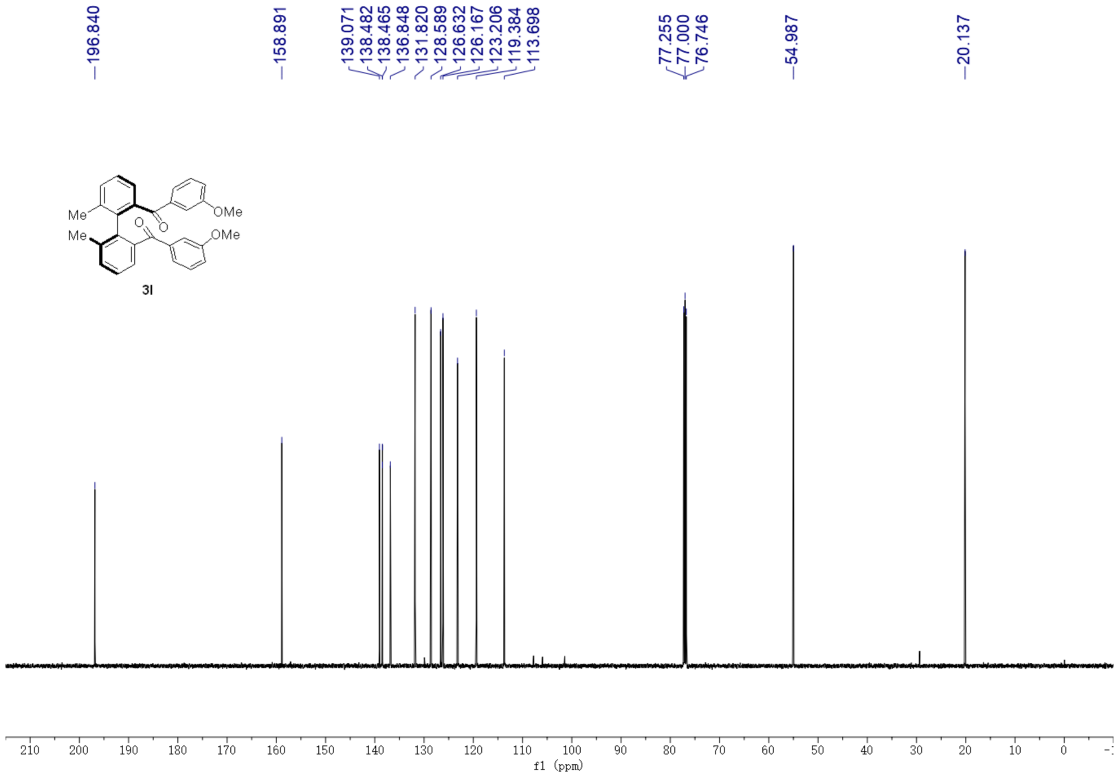
196.840
158.891
139.071
138.482
138.465
136.848
131.820
128.589
126.632
126.167
123.206
119.384
113.698
77.255
77.000
76.746
54.987
20.137
Me
OMe
Me
OMe
3l
f1 (ppm)

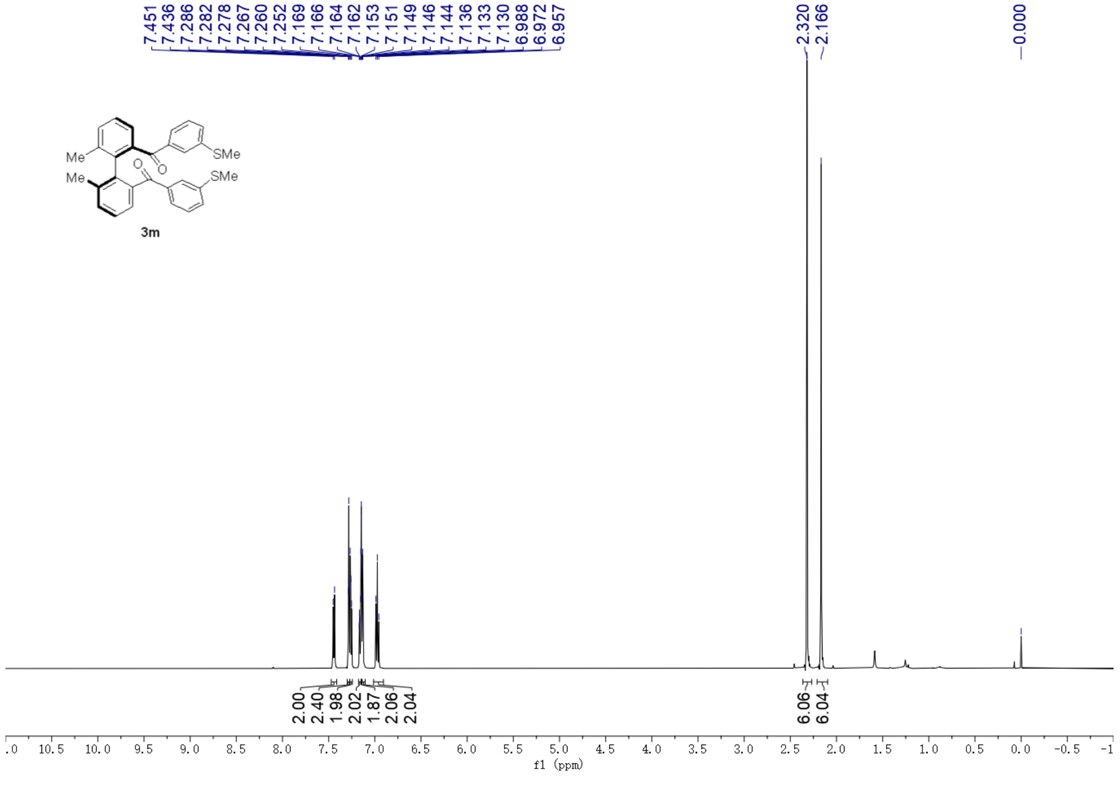
7.451
7.436
7.286
7.282
7.278
7.267
7.260
7.252
7.169
7.166
7.164
7.162
7.153
7.151
7.149
7.146
7.144
7.136
7.133
7.130
6.988
6.972
6.957
2.320
2.166
0.000
Me
SMe
Me
SMe
3m
2.00
2.40
1.98
2.02
1.87
2.06
2.04
6.06
6.04
f1 (ppm)

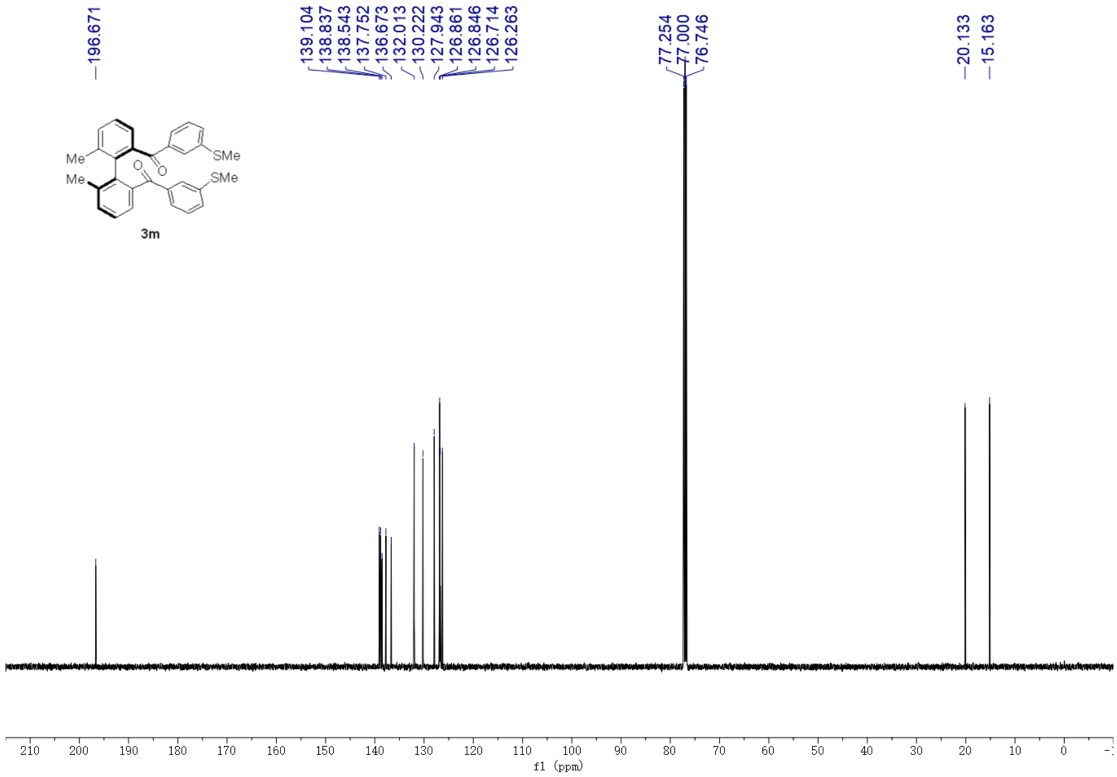
196.671
139.104
138.837
138.543
137.752
136.673
132.013
130.222
127.943
126.861
126.846
126.714
126.263
77.254
77.000
76.746
20.133
15.163
Me
Me
SMe
SMe
O
O
3m
210 200 190 180 170 160 150 140 130 120 110 100 90 80 70 60 50 40 30 20 10 0
f1 (ppm)

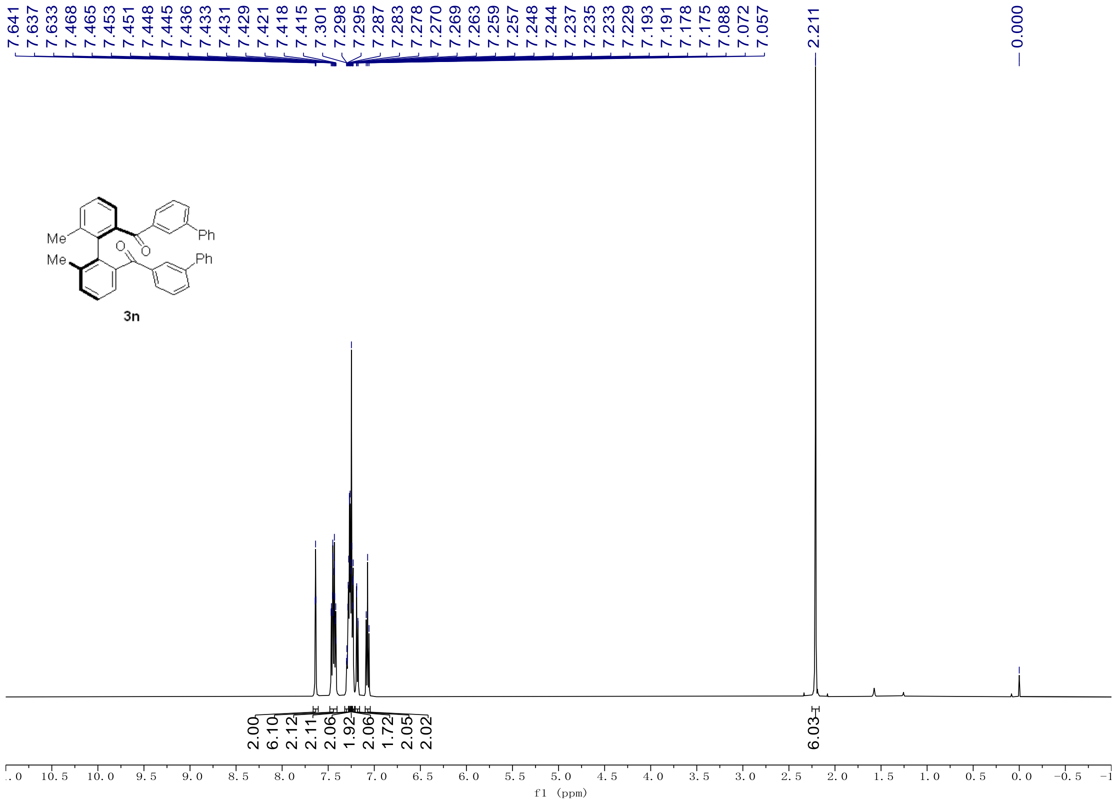
7.641
7.637
7.633
7.468
7.465
7.453
7.451
7.448
7.445
7.436
7.433
7.431
7.429
7.421
7.418
7.415
7.301
7.298
7.295
7.287
7.283
7.278
7.270
7.269
7.263
7.259
7.257
7.248
7.244
7.237
7.235
7.233
7.229
7.193
7.191
7.178
7.175
7.088
7.072
7.057
2.211
0.000
Me
Me
Ph
Ph
O
O
3n
2.00
6.10
2.12
2.11
2.06
1.92
2.06
1.72
2.05
2.02
6.03
10.5 10.0 9.5 9.0 8.5 8.0 7.5 7.0 6.5 6.0 5.5 5.0 4.5 4.0 3.5 3.0 2.5 2.0 1.5 1.0 0.5 0.0 -0.5
f1 (ppm)

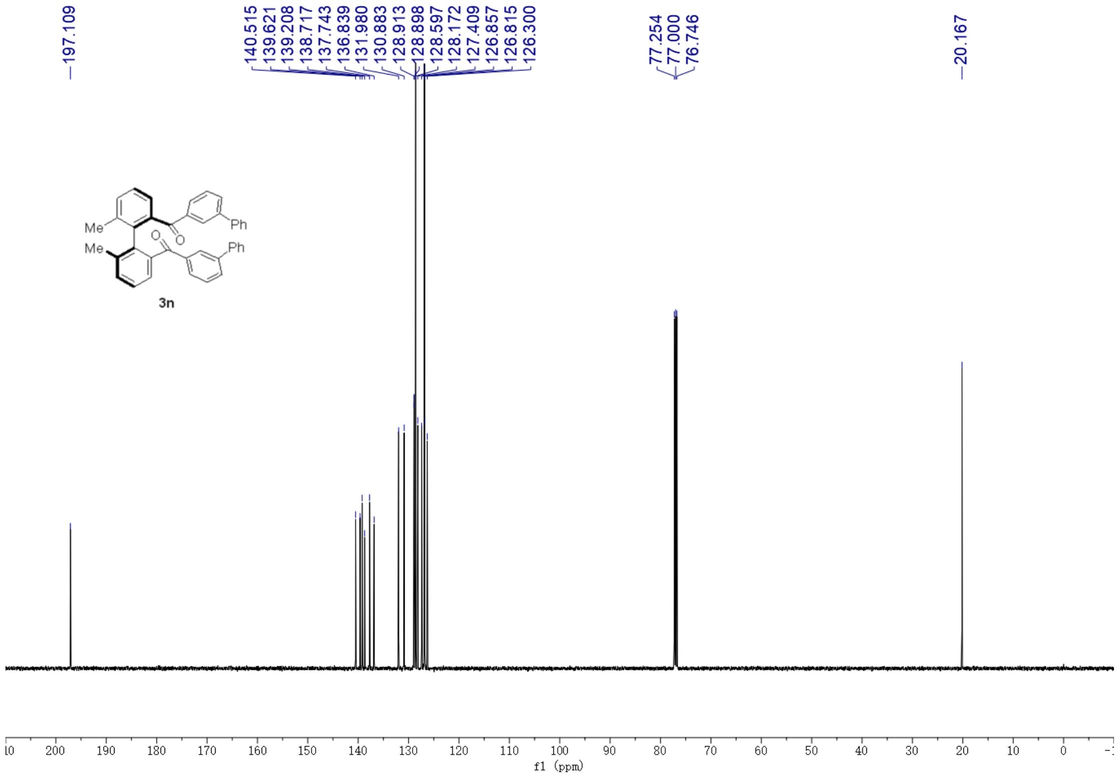

197.109
140.515
139.621
139.208
138.717
137.743
136.839
131.980
130.883
128.913
128.898
128.597
128.172
127.409
126.857
126.815
126.300
77.254
77.000
76.746
20.167
Me
Me
O
O
Ph
Ph
3n
f1 (ppm)

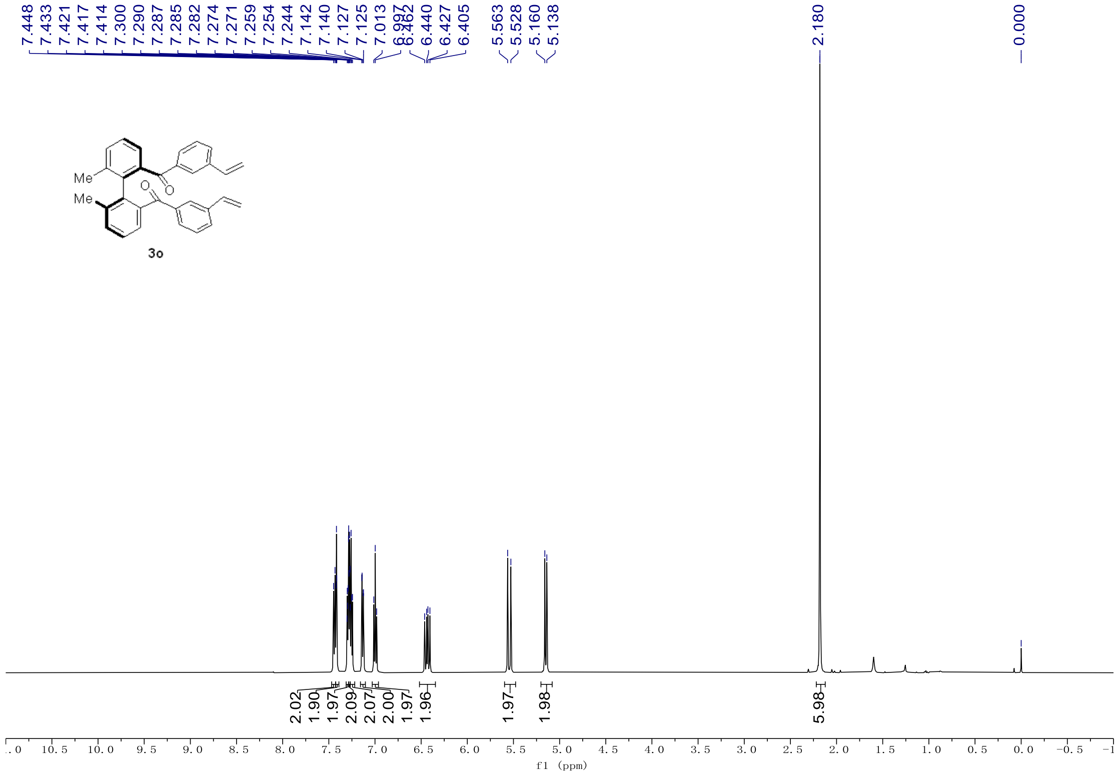

7.448
7.433
7.421
7.417
7.414
7.300
7.290
7.287
7.285
7.282
7.274
7.271
7.259
7.254
7.244
7.142
7.140
7.127
7.125
7.013
6.997
6.462
6.440
6.427
6.405
5.563
5.528
5.160
5.138
2.180
0.000
Me
Me
O
O
3o
2.02
1.90
1.97
2.09
2.07
2.00
1.97
1.96
1.97
1.98
5.98
f1 (ppm)

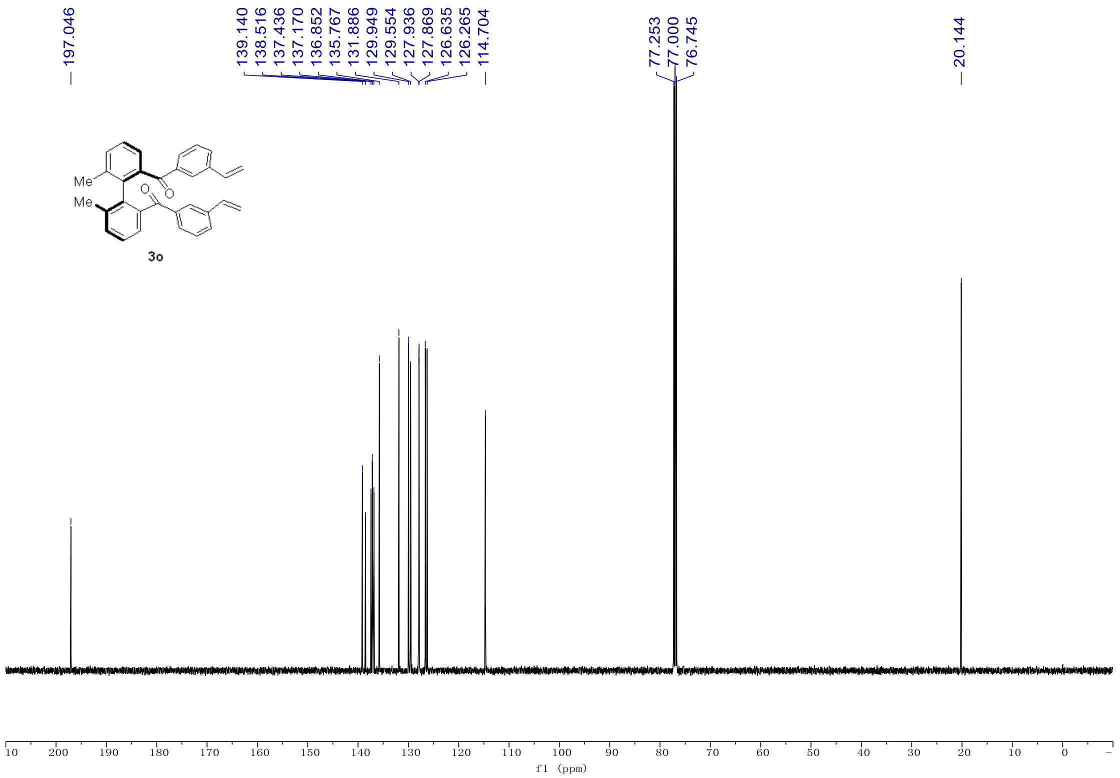

197.046
139.140
138.516
137.436
137.170
136.852
135.767
131.886
129.949
129.554
127.936
127.869
126.635
126.265
114.704
77.253
77.000
76.745
20.144
Me
Me
O
O
3o
f1 (ppm)

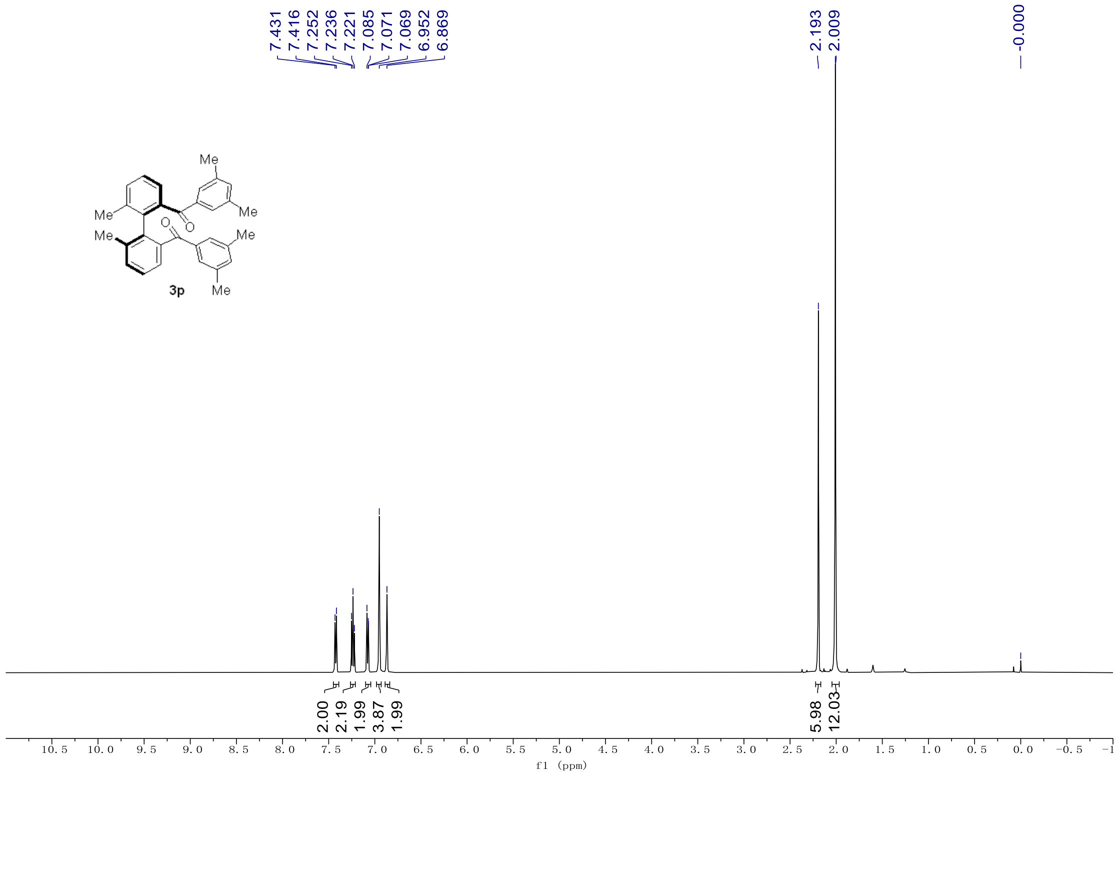

7.431
7.416
7.252
7.236
7.221
7.085
7.071
7.069
6.952
6.869
2.193
2.009
-0.000
Me
Me
Me
Me
Me
Me
O
O
3p
2.00
2.19
1.99
3.87
1.99
5.98
12.03
f1 (ppm)

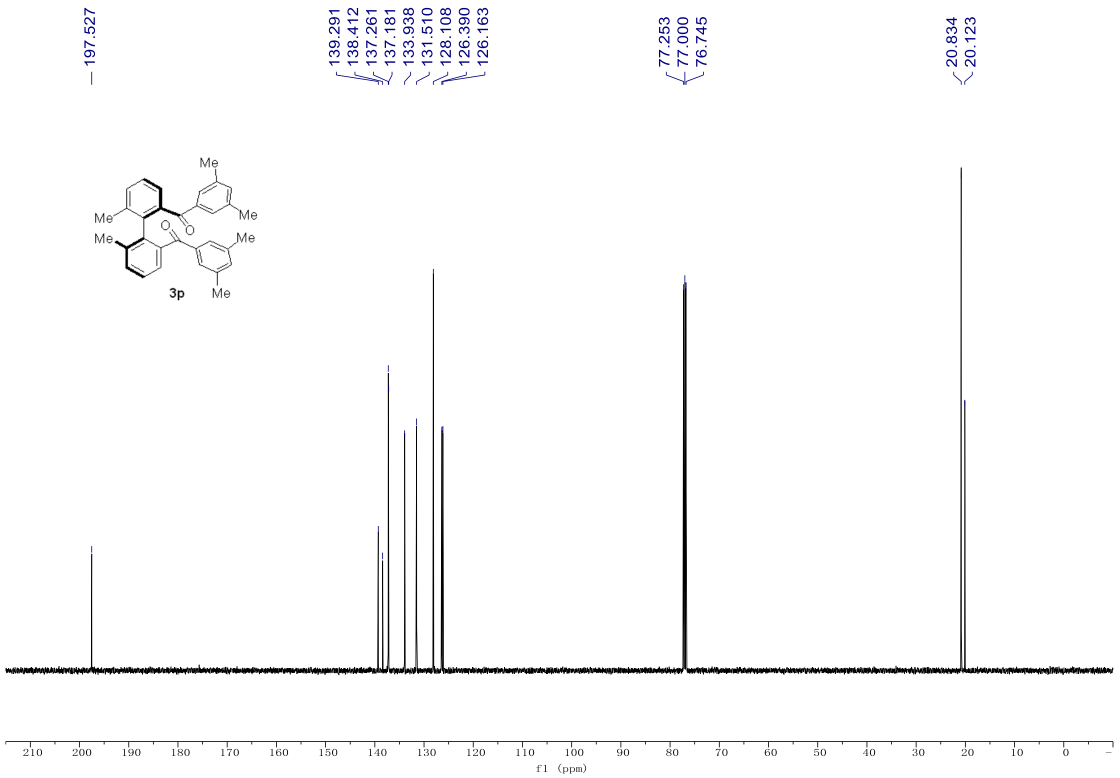
197.527
139.291
138.412
137.261
137.181
133.938
131.510
128.108
126.390
126.163
77.253
77.000
76.745
20.834
20.123
Me
Me
Me
Me
Me
Me
O
O
3p
210 200 190 180 170 160 150 140 130 120 110 100 90 80 70 60 50 40 30 20 10 0
f1 (ppm)

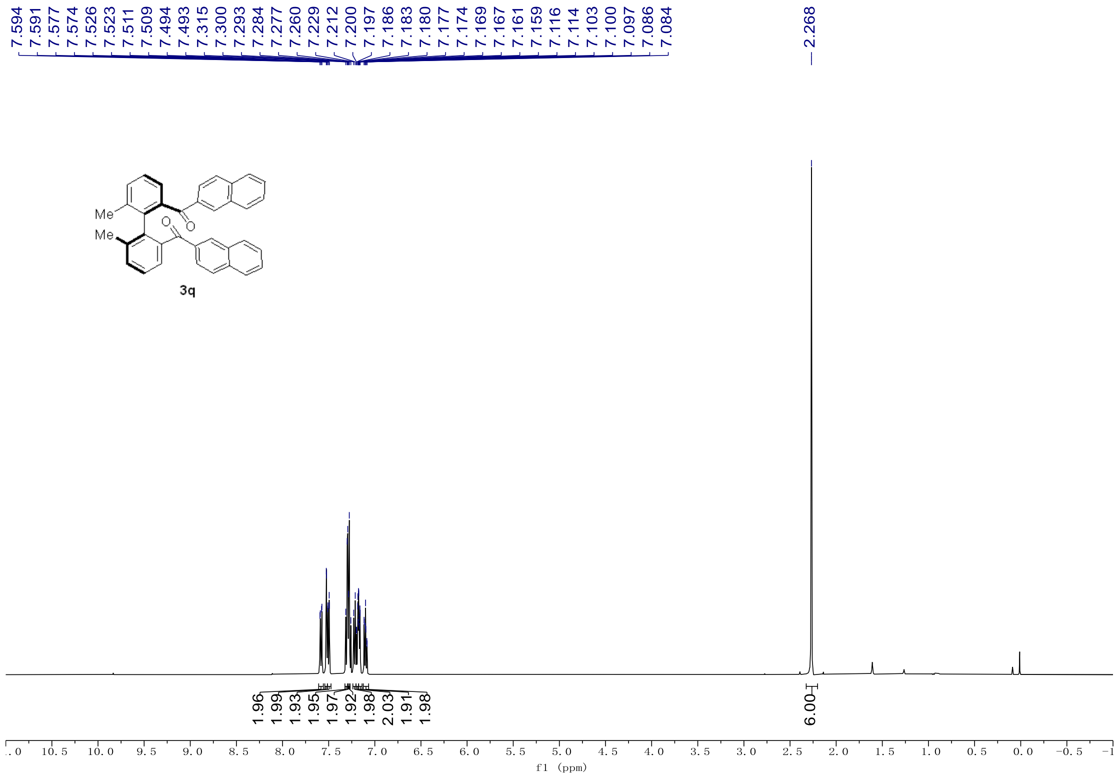
7.594
7.591
7.577
7.574
7.526
7.523
7.511
7.509
7.494
7.493
7.315
7.300
7.293
7.284
7.277
7.260
7.229
7.212
7.200
7.197
7.186
7.183
7.180
7.177
7.174
7.169
7.167
7.161
7.159
7.116
7.114
7.103
7.100
7.097
7.086
7.084
2.268
Me
Me
O
O
3q
1.96
1.99
1.93
1.95
1.97
1.92
1.98
2.03
1.91
1.98
6.00
10.5 10.0 9.5 9.0 8.5 8.0 7.5 7.0 6.5 6.0 5.5 5.0 4.5 4.0 3.5 3.0 2.5 2.0 1.5 1.0 0.5 0.0 -0.5
f1 (ppm)

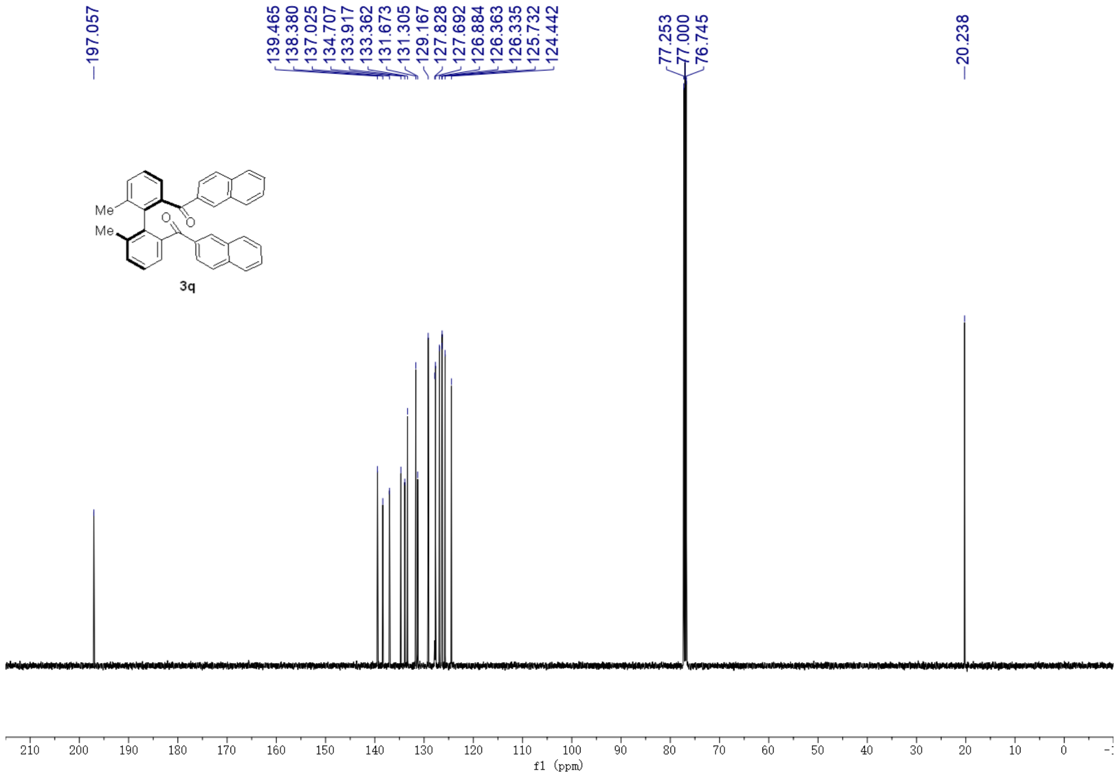
197.057
139.465
138.380
137.025
134.707
133.917
133.362
131.673
131.305
129.167
127.828
127.692
126.884
126.363
126.335
125.732
124.442
77.253
77.000
76.745
20.238
Me
Me
3q
210 200 190 180 170 160 150 140 130 120 110 100 90 80 70 60 50 40 30 20 10 0
f1 (ppm)

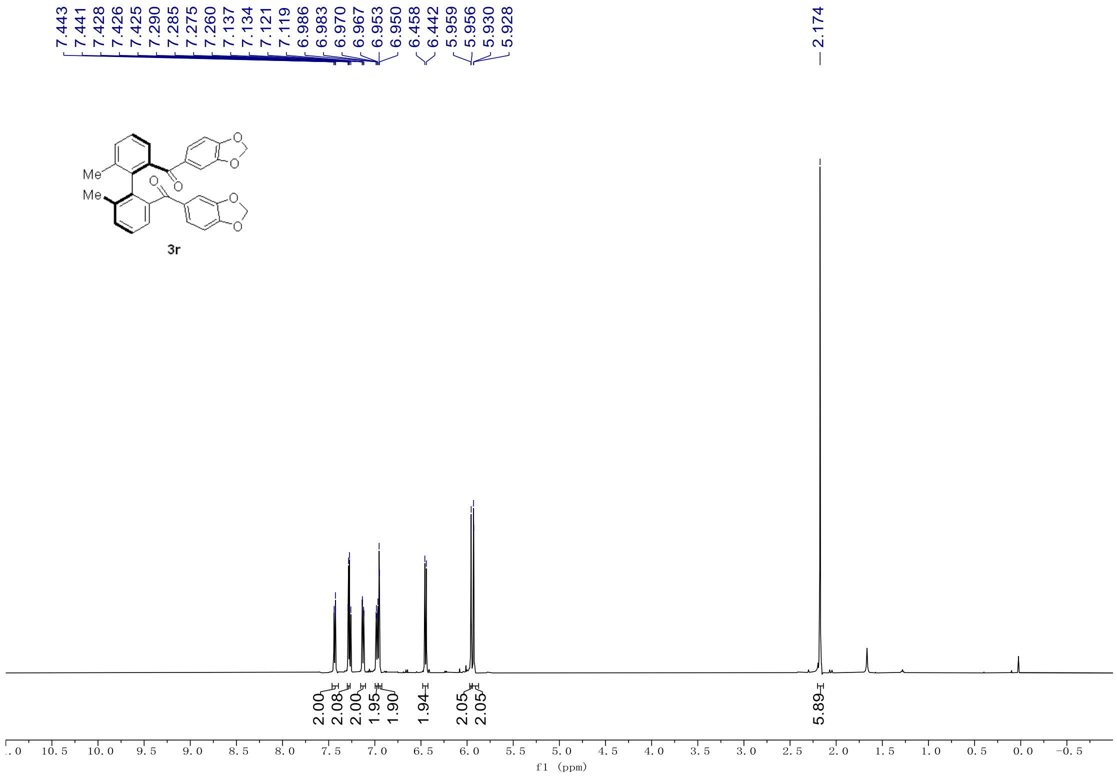
7.443
7.441
7.428
7.426
7.425
7.290
7.285
7.275
7.260
7.137
7.134
7.121
7.119
6.986
6.983
6.970
6.967
6.953
6.950
6.458
6.442
5.959
5.956
5.930
5.928
2.174
Me
Me
3r
2.00
2.08
2.00
1.95
1.90
1.94
2.05
2.05
5.89
10.5 10.0 9.5 9.0 8.5 8.0 7.5 7.0 6.5 6.0 5.5 5.0 4.5 4.0 3.5 3.0 2.5 2.0 1.5 1.0 0.5 0.0 -0.5
f1 (ppm)

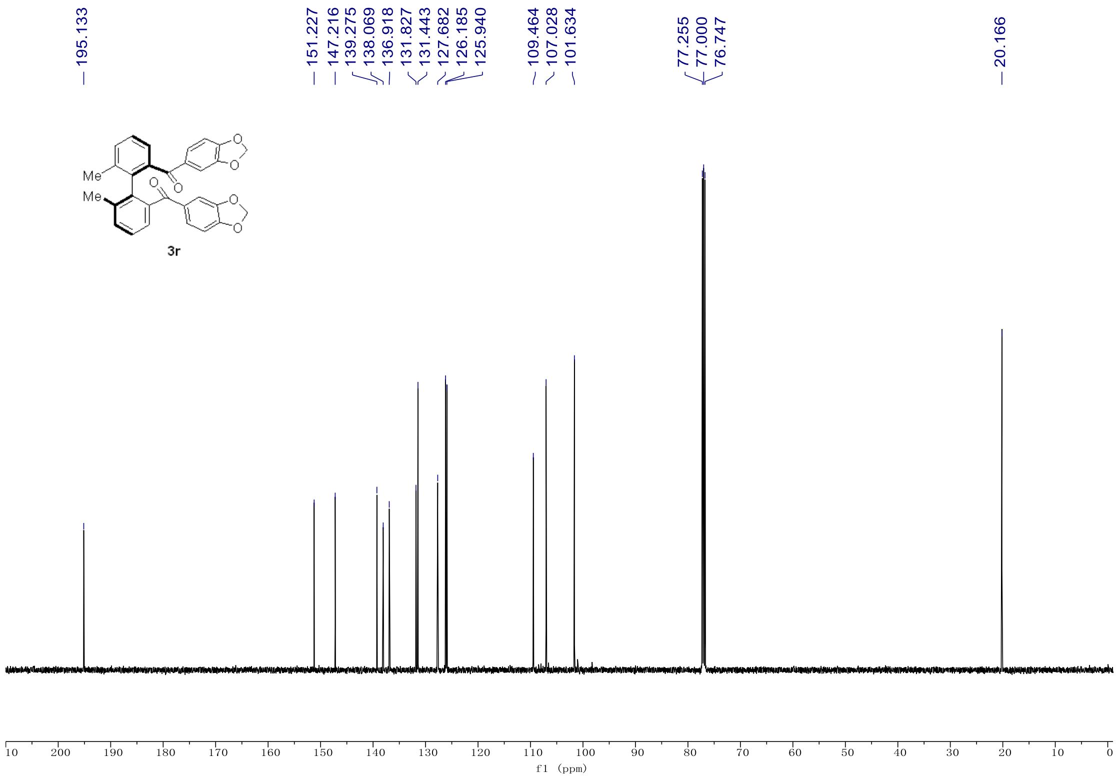
195.133
151.227
147.216
139.275
138.069
136.918
131.827
131.443
127.682
126.185
125.940
109.464
107.028
101.634
77.255
77.000
76.747
20.166
Me
Me
O
O
O
O
O
O
3r
f1 (ppm)

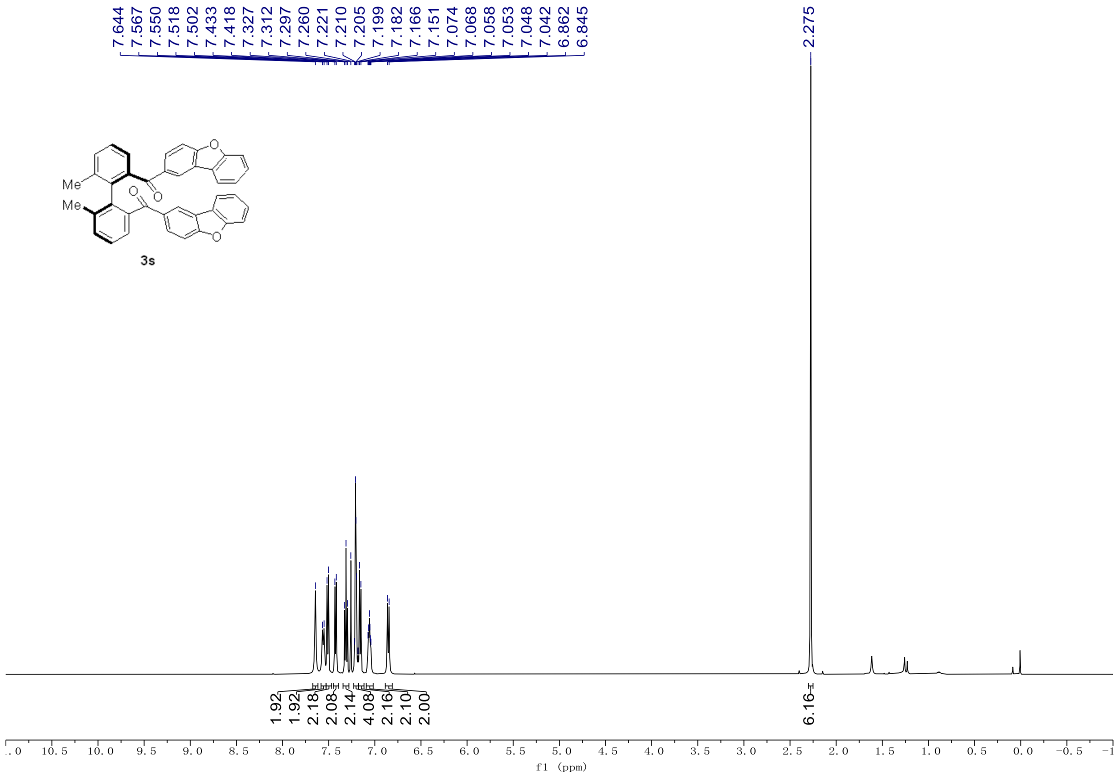
7.644
7.567
7.550
7.518
7.502
7.433
7.418
7.327
7.312
7.297
7.260
7.221
7.210
7.205
7.199
7.182
7.166
7.151
7.074
7.068
7.058
7.053
7.048
7.042
6.862
6.845
2.275
Me
Me
O
O
O
O
3s
1.92
1.92
2.18
2.08
2.14
4.08
2.16
2.10
2.00
6.16
f1 (ppm)

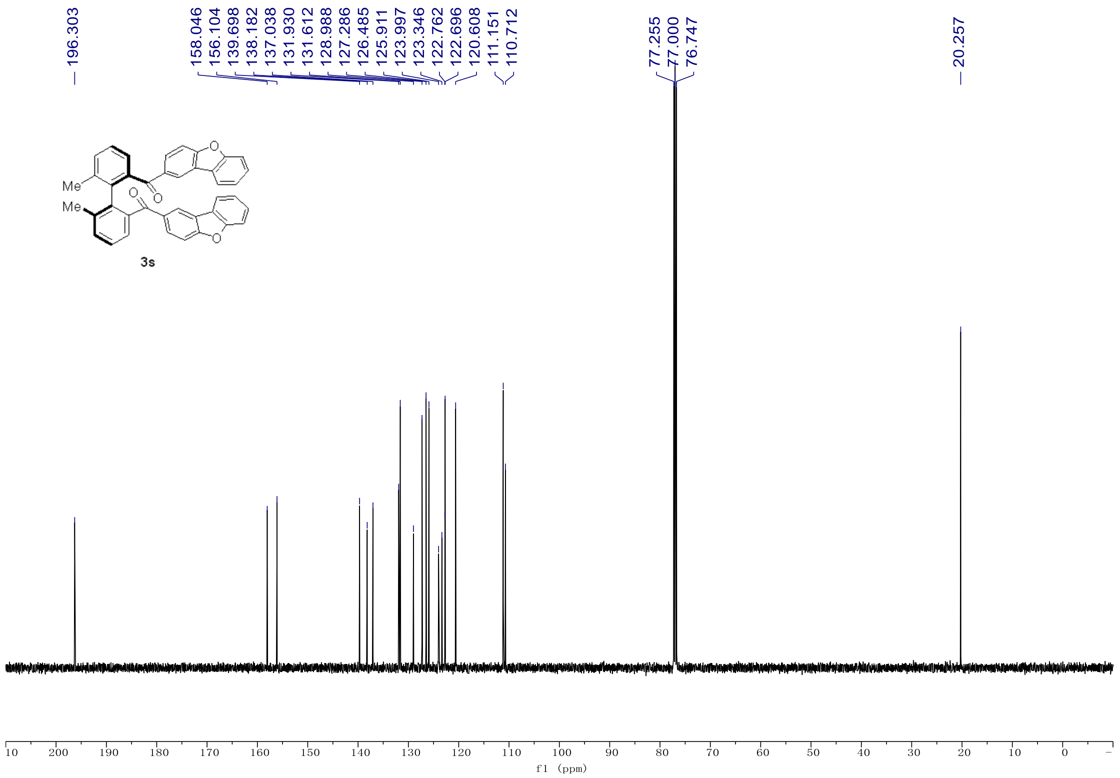
196.303
158.046
156.104
139.698
138.182
137.038
131.930
131.612
128.988
127.286
126.485
125.911
123.997
123.346
122.762
122.696
120.608
111.151
110.712
77.255
77.000
76.747
20.257
Me
Me
O
O
O
O
3s
f1 (ppm)

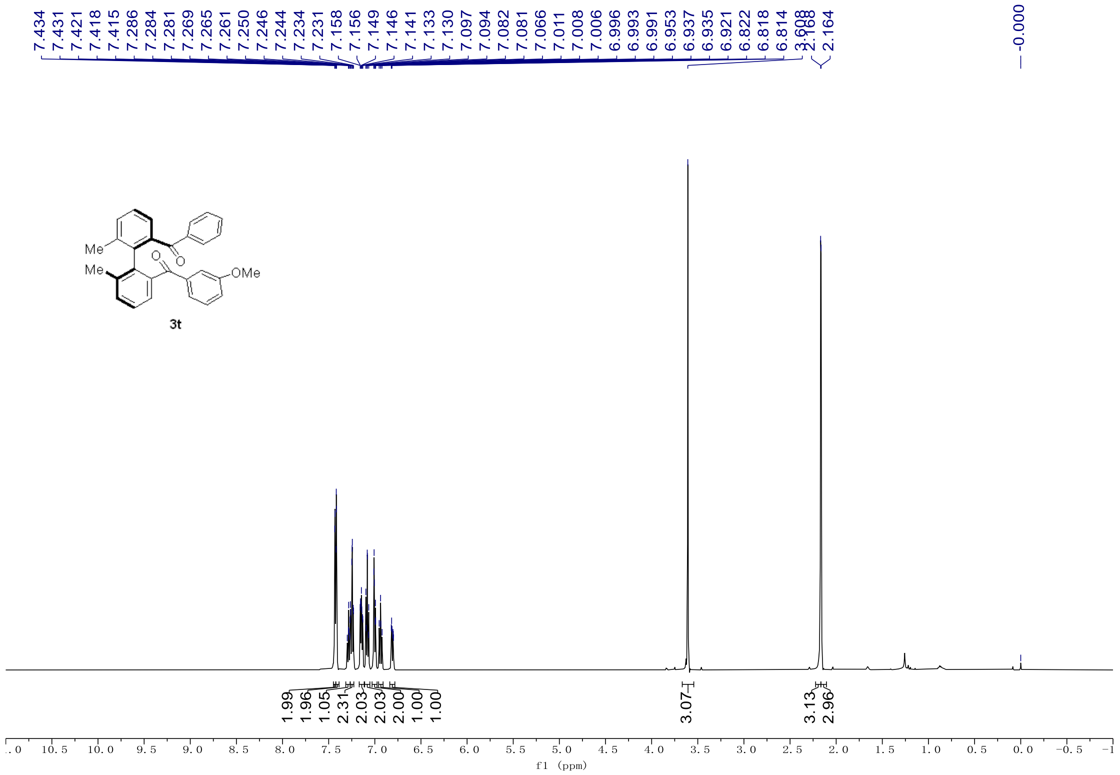
7.434
7.431
7.421
7.418
7.415
7.286
7.284
7.281
7.269
7.265
7.261
7.250
7.246
7.244
7.234
7.231
7.158
7.156
7.149
7.146
7.141
7.133
7.130
7.097
7.094
7.082
7.081
7.066
7.011
7.008
7.006
6.996
6.993
6.991
6.953
6.937
6.935
6.921
6.822
6.818
6.814
3.608
2.168
2.164
-0.000
Me
Me
O
O
OMe
3t
1.99
1.96
1.05
2.31
2.03
2.03
2.00
1.00
1.00
3.07
3.13
2.96
f1 (ppm)

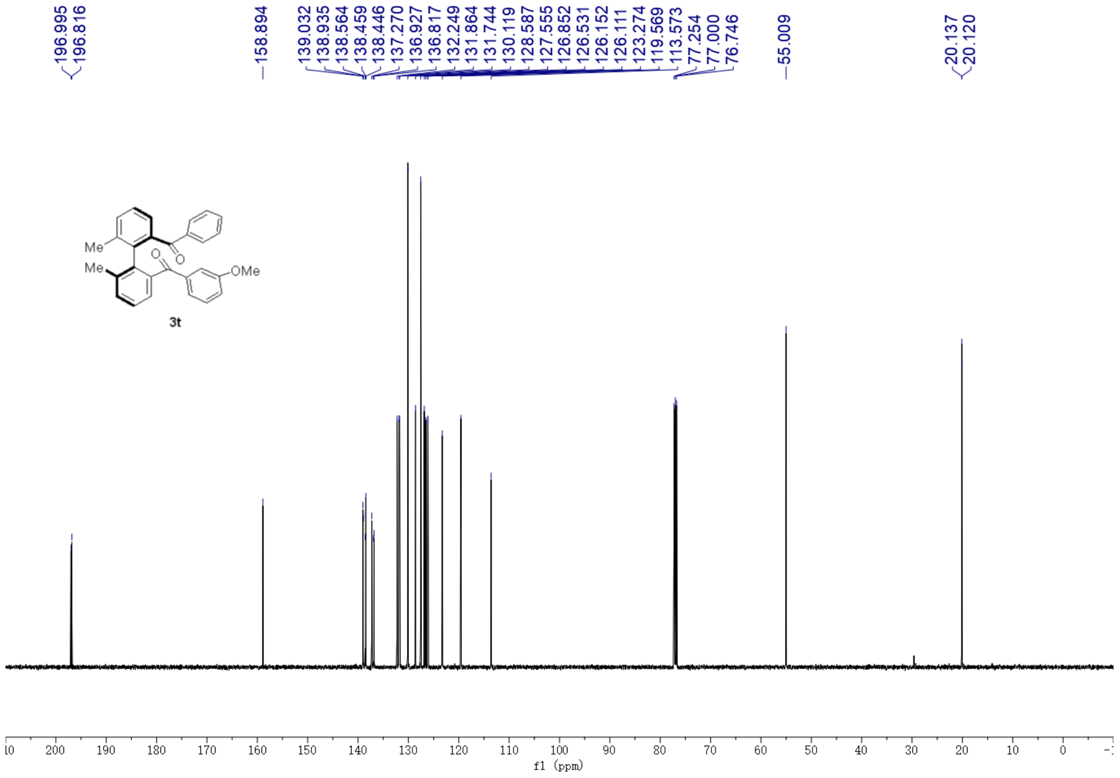
196.995
196.816
158.894
139.032
138.935
138.564
138.459
138.446
137.270
136.927
136.817
132.249
131.864
131.744
130.119
128.587
127.555
126.852
126.531
126.152
126.111
123.274
119.569
113.573
77.254
77.000
76.746
55.009
20.137
20.120
Me
Me
O
O
OMe
3t
f1 (ppm)

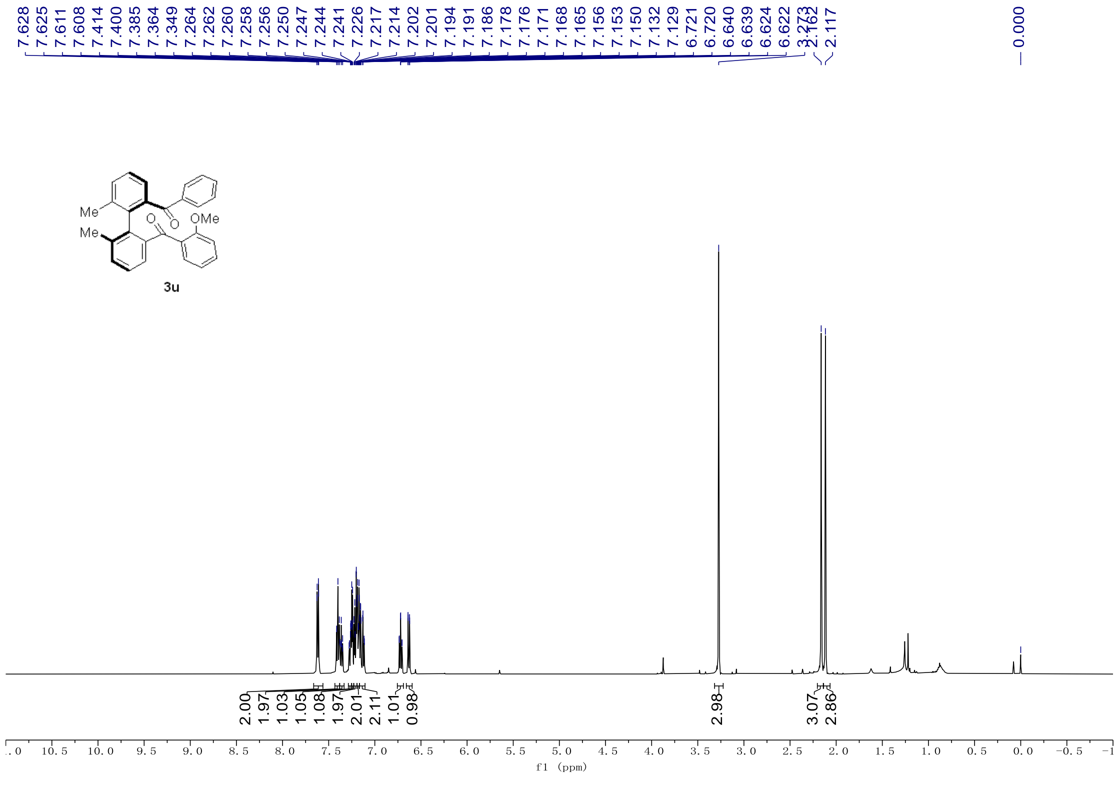
7.628
7.625
7.611
7.608
7.414
7.400
7.385
7.364
7.349
7.264
7.262
7.260
7.258
7.256
7.250
7.247
7.244
7.241
7.226
7.217
7.214
7.202
7.201
7.194
7.191
7.186
7.178
7.176
7.171
7.168
7.165
7.156
7.153
7.150
7.132
7.129
6.721
6.720
6.640
6.639
6.624
6.622
3.273
2.162
2.117
0.000
Me
Me
O
O
OMe
3u
2.00
1.97
1.03
1.05
1.08
1.97
2.01
2.11
1.01
0.98
2.98
3.07
2.86
f1 (ppm)

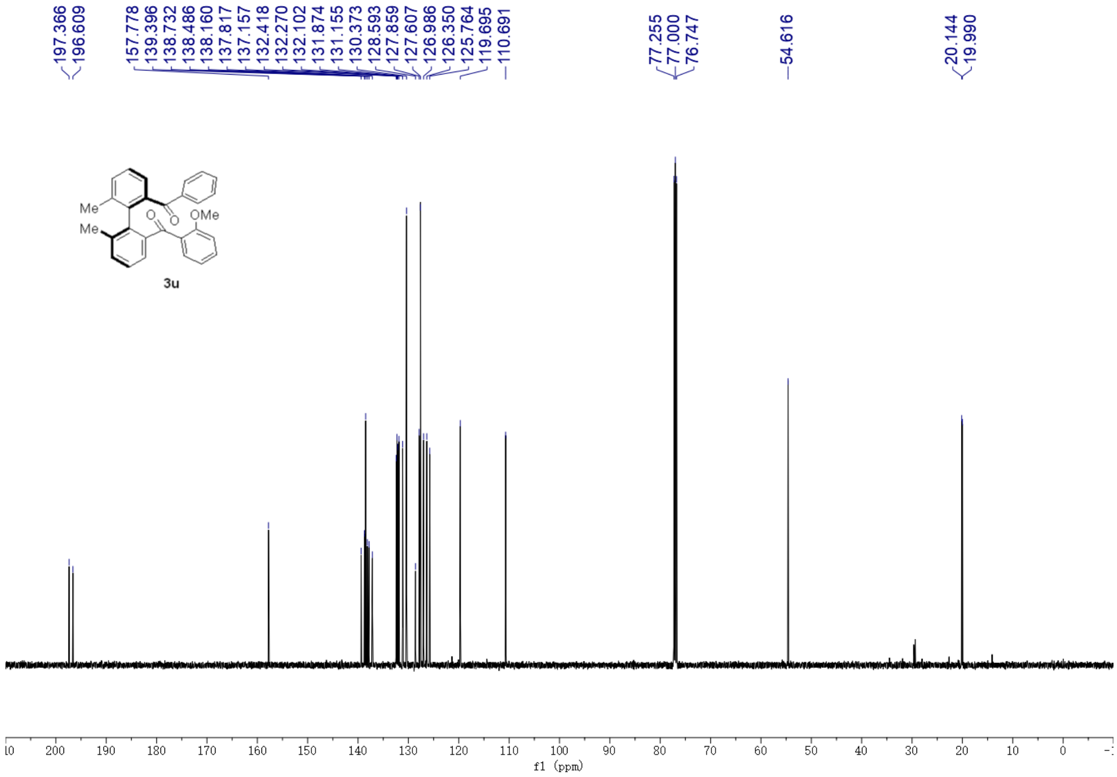
197.366
196.609
157.778
139.396
138.732
138.486
138.160
137.817
137.157
132.418
132.270
132.102
131.874
131.155
130.373
128.593
127.859
127.607
126.986
126.350
125.764
119.695
110.691
77.255
77.000
76.747
54.616
20.144
19.990
Me
Me
OMe
O
O
3u
f1 (ppm)

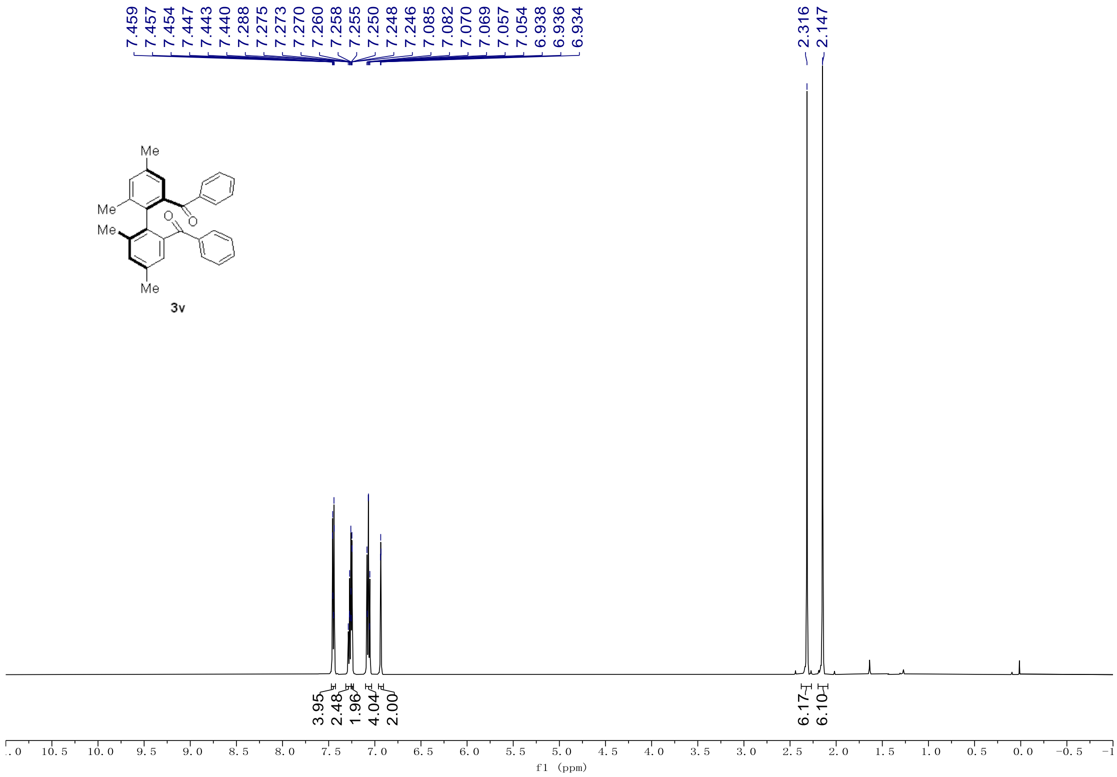
7.459
7.457
7.454
7.447
7.443
7.440
7.288
7.275
7.273
7.270
7.260
7.258
7.255
7.250
7.248
7.246
7.085
7.082
7.070
7.069
7.057
7.054
6.938
6.936
6.934
2.316
2.147
Me
Me
Me
Me
O
O
3v
3.95
2.48
1.96
4.04
2.00
6.17
6.10
f1 (ppm)

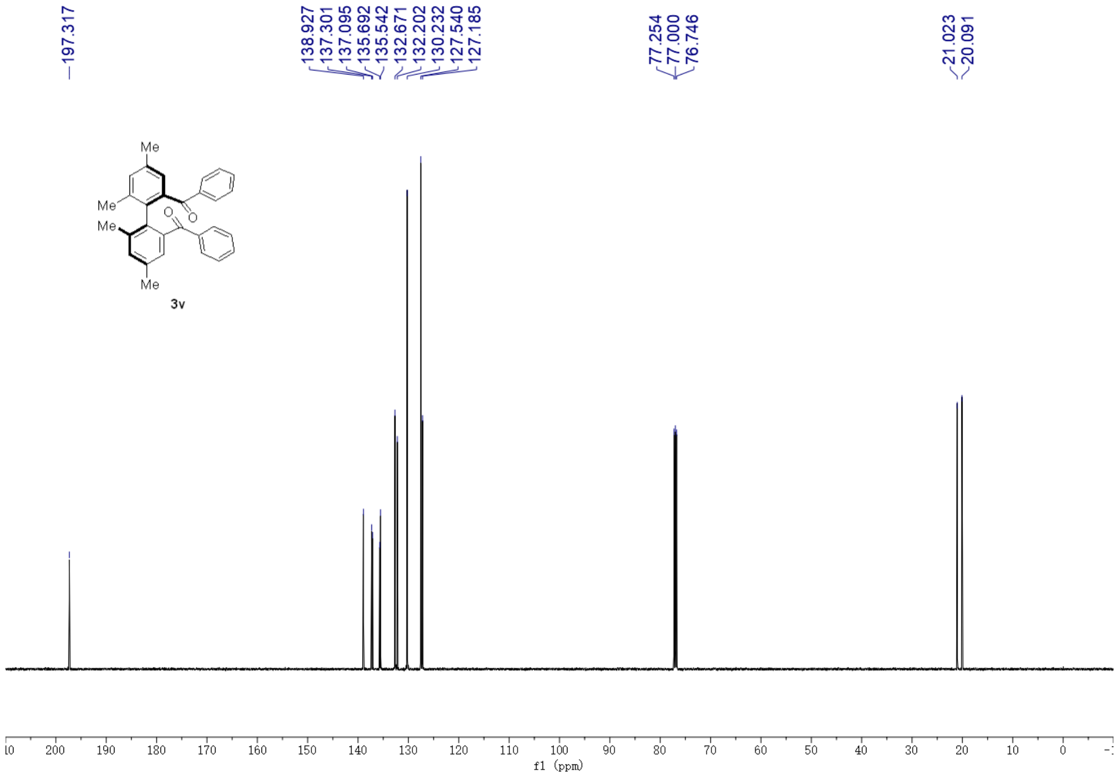
197.317
138.927
137.301
137.095
135.692
135.542
132.671
132.202
130.232
127.540
127.185
77.254
77.000
76.746
21.023
20.091
Me
Me
Me
Me
O
O
3v
f1 (ppm)

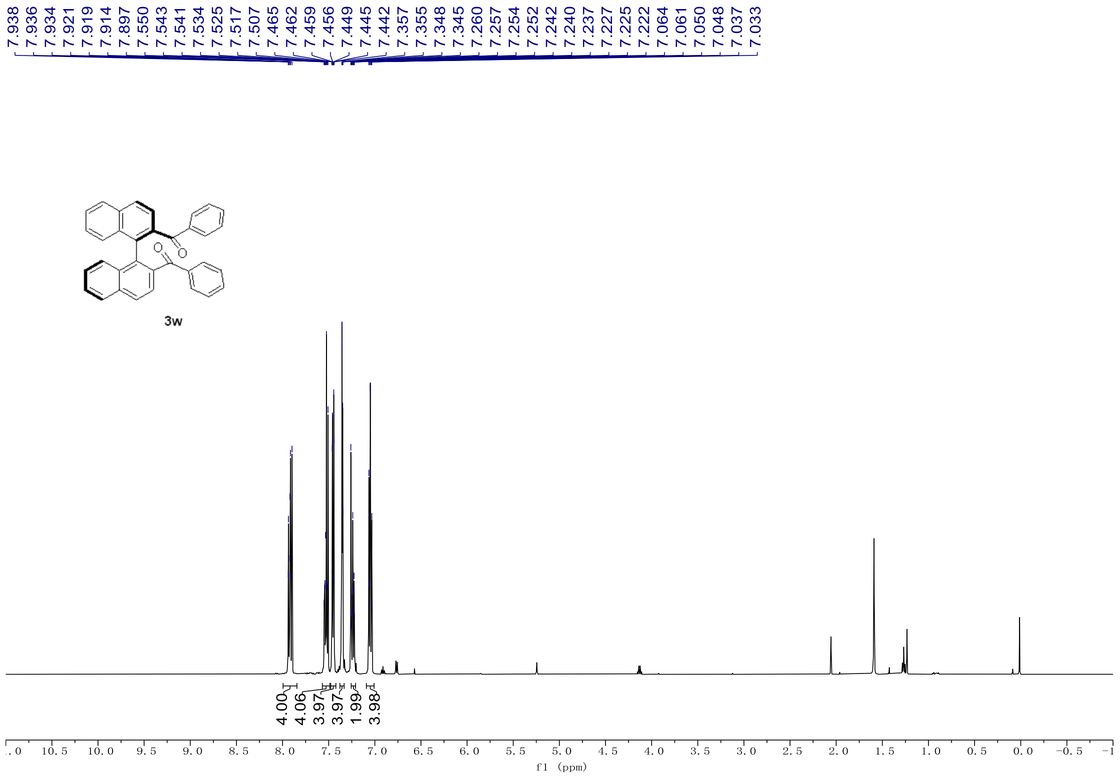
7.938
7.936
7.934
7.921
7.919
7.914
7.897
7.550
7.543
7.541
7.534
7.525
7.517
7.507
7.465
7.462
7.459
7.456
7.449
7.445
7.442
7.357
7.355
7.348
7.345
7.260
7.257
7.254
7.252
7.242
7.240
7.237
7.227
7.225
7.222
7.064
7.061
7.050
7.048
7.037
7.033
O
O
3w
4.00
4.06
3.97
3.97
1.99
3.98
f1 (ppm)

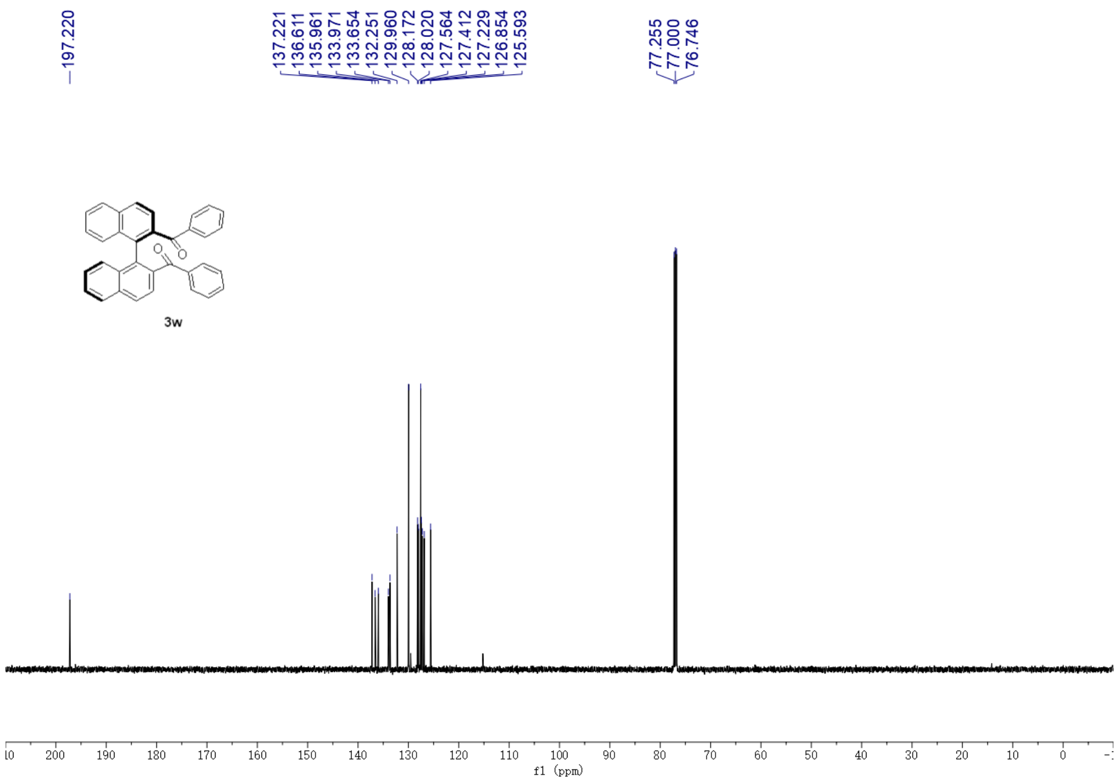
197.220
137.221
136.611
135.961
133.971
133.654
132.251
129.960
128.172
128.020
127.564
127.412
127.229
126.854
125.593
77.255
77.000
76.746
O
O
3w
200 190 180 170 160 150 140 130 120 110 100 90 80 70 60 50 40 30 20 10 0
f1 (ppm)

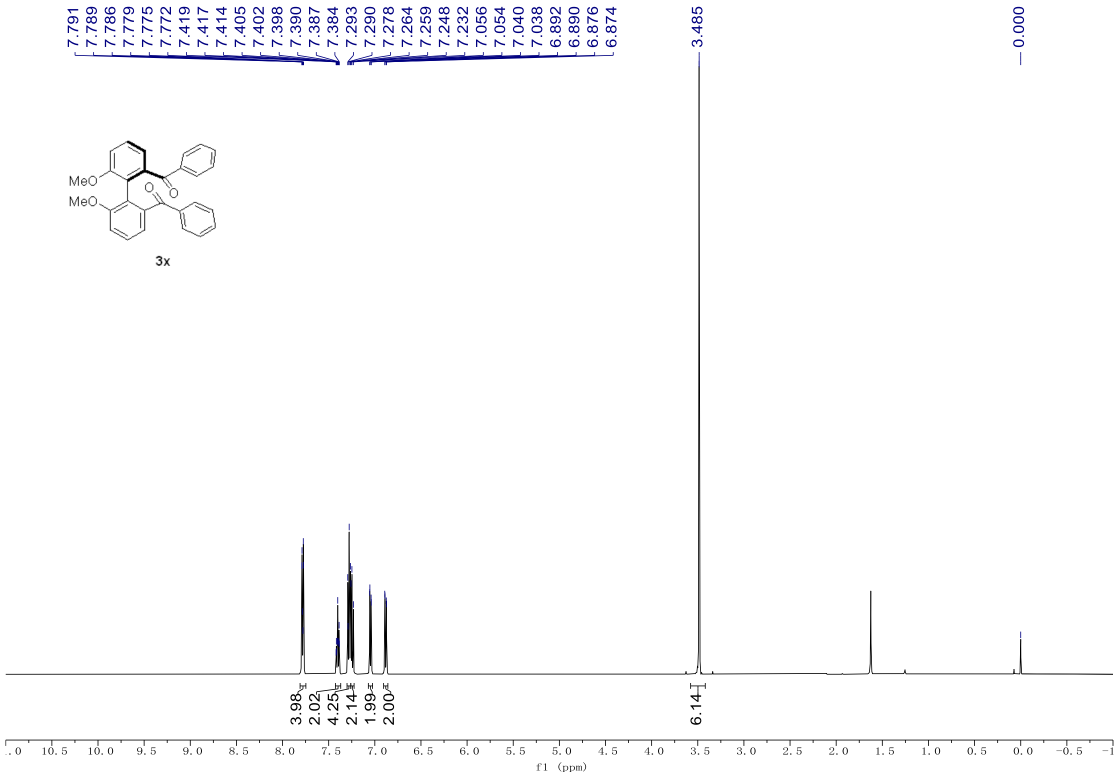
7.791
7.789
7.786
7.779
7.775
7.772
7.419
7.417
7.414
7.405
7.402
7.398
7.390
7.387
7.384
7.293
7.290
7.278
7.264
7.259
7.248
7.232
7.056
7.054
7.040
7.038
6.892
6.890
6.876
6.874
3.485
0.000
MeO
MeO
O
O
3x
3.98
2.02
4.25
2.14
1.99
2.00
6.14
10.5 10.0 9.5 9.0 8.5 8.0 7.5 7.0 6.5 6.0 5.5 5.0 4.5 4.0 3.5 3.0 2.5 2.0 1.5 1.0 0.5 0.0 -0.5
f1 (ppm)

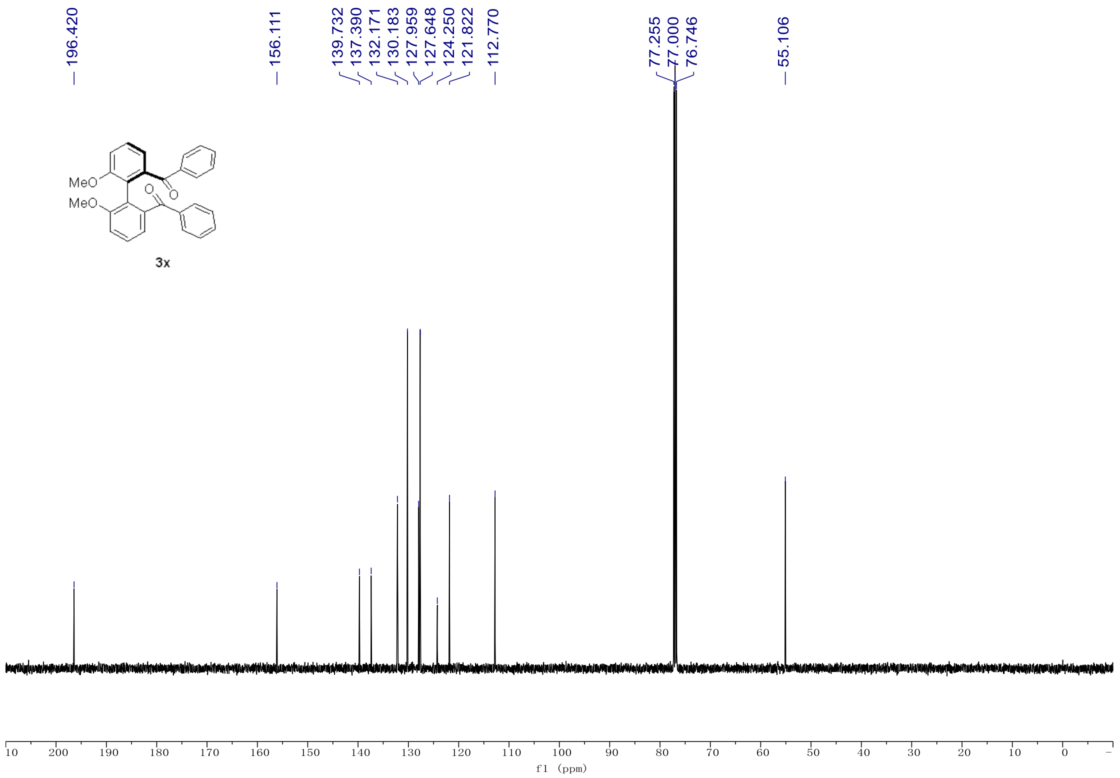
196.420
156.111
139.732
137.390
132.171
130.183
127.959
127.648
124.250
121.822
112.770
77.255
77.000
76.746
55.106
MeO
MeO
O
O
3x
f1 (ppm)

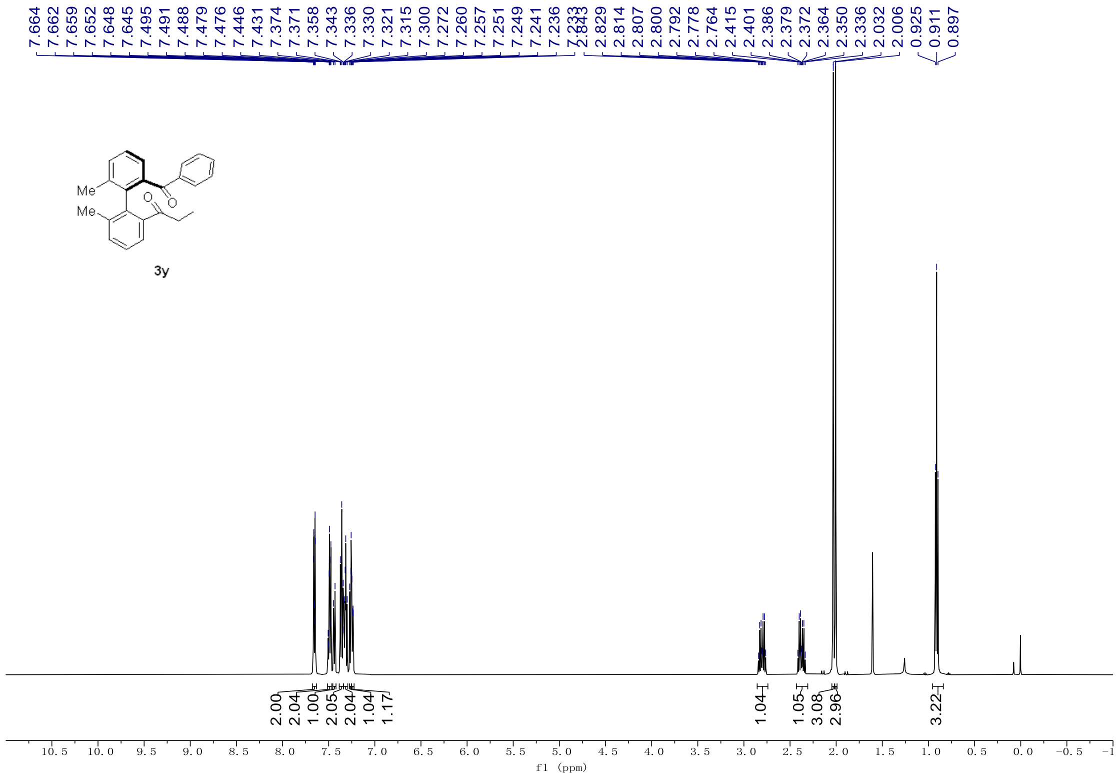
7.664
7.662
7.659
7.652
7.648
7.645
7.495
7.491
7.488
7.479
7.476
7.446
7.431
7.374
7.371
7.358
7.343
7.336
7.330
7.321
7.315
7.300
7.272
7.260
7.257
7.251
7.249
7.241
7.236
7.233
2.843
2.829
2.814
2.807
2.800
2.792
2.778
2.764
2.415
2.401
2.386
2.379
2.372
2.364
2.350
2.336
2.032
2.006
0.925
0.911
0.897
Me
Me
O
O
3y
2.00
2.04
1.00
2.05
2.04
1.04
1.17
1.04
1.05
3.08
2.96
3.22
f1 (ppm)

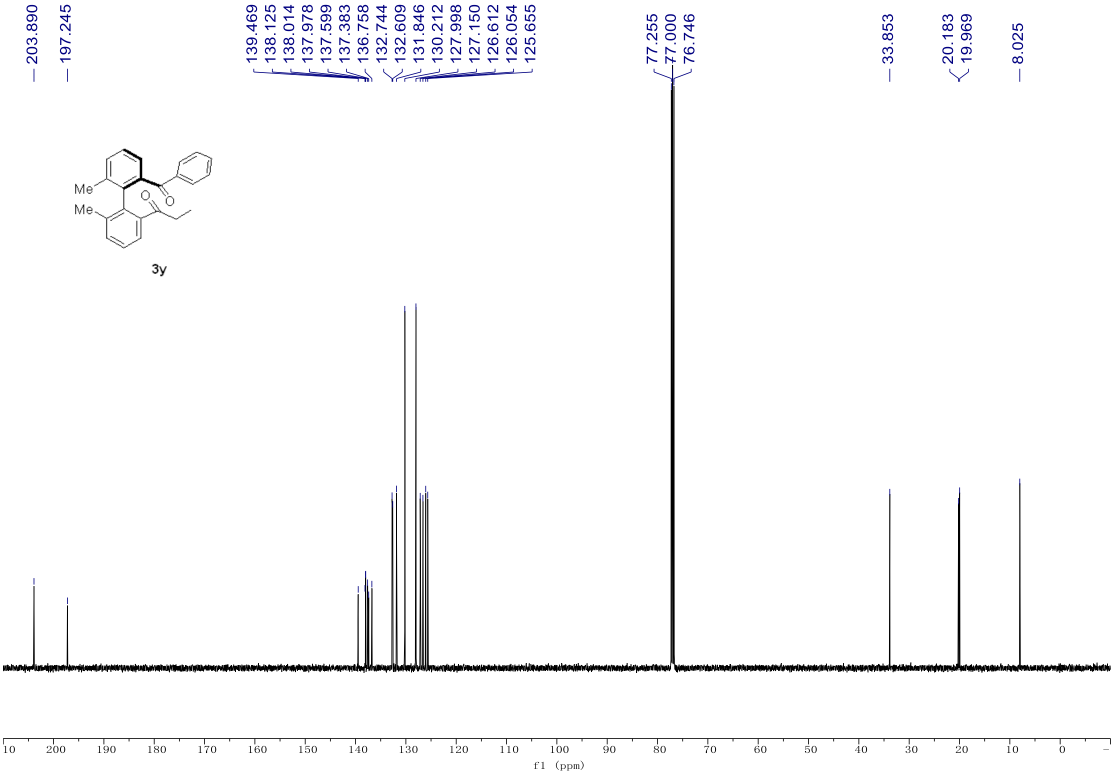
203.890
197.245
139.469
138.125
138.014
137.978
137.599
137.383
136.758
132.744
132.609
131.846
130.212
127.998
127.150
126.612
126.054
125.655
77.255
77.000
76.746
33.853
20.183
19.969
8.025
Me
Me
O
O
3y
f1 (ppm)

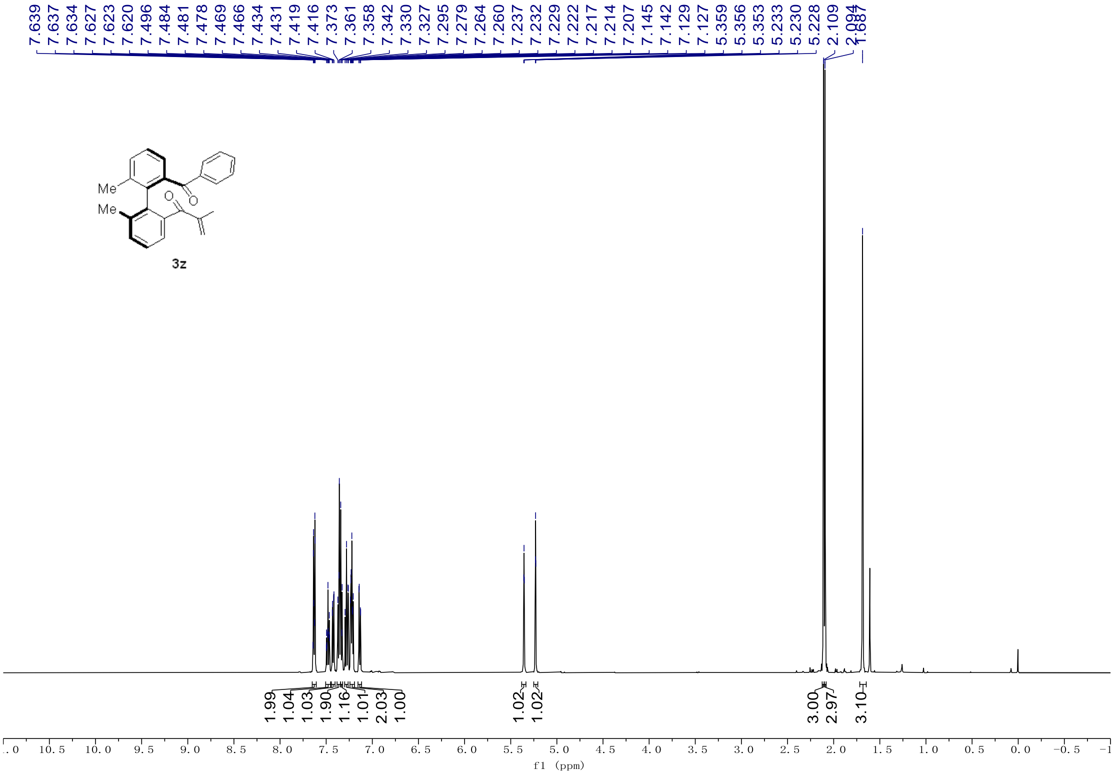
7.639
7.637
7.634
7.627
7.623
7.620
7.496
7.484
7.481
7.478
7.469
7.466
7.434
7.431
7.419
7.416
7.373
7.361
7.358
7.342
7.330
7.327
7.295
7.279
7.264
7.260
7.237
7.232
7.229
7.222
7.217
7.214
7.207
7.145
7.142
7.129
7.127
5.359
5.356
5.353
5.233
5.230
5.228
2.109
2.094
1.687
Me
Me
O
O
3z
1.99
1.04
1.03
1.90
1.16
1.01
2.03
1.00
1.02
1.02
3.00
2.97
3.10
f1 (ppm)

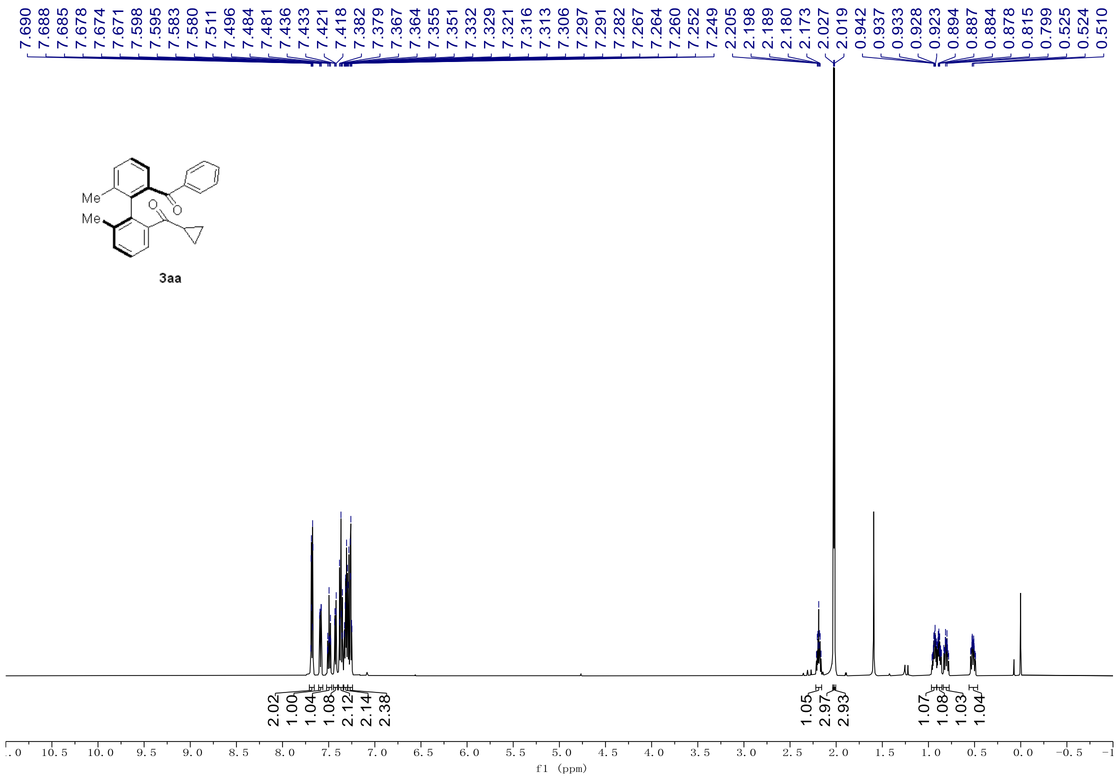
7.690 7.688 7.685 7.678 7.674 7.671 7.598 7.595 7.583 7.580 7.511 7.496 7.484 7.481 7.436 7.433 7.421 7.418 7.382 7.379 7.367 7.364 7.355 7.351 7.332 7.329 7.321 7.316 7.313 7.306 7.297 7.291 7.282 7.267 7.264 7.260 7.252 7.249 2.205 2.198 2.189 2.180 2.173 2.027 2.019 0.942 0.937 0.933 0.928 0.923 0.894 0.887 0.884 0.878 0.815 0.799 0.525 0.524 0.510
Me
Me
O
O
3aa
2.02 1.00 1.04 1.08 2.12 2.14 2.38
1.05 2.97 2.93
1.07 1.08 1.03 1.04
f1 (ppm)

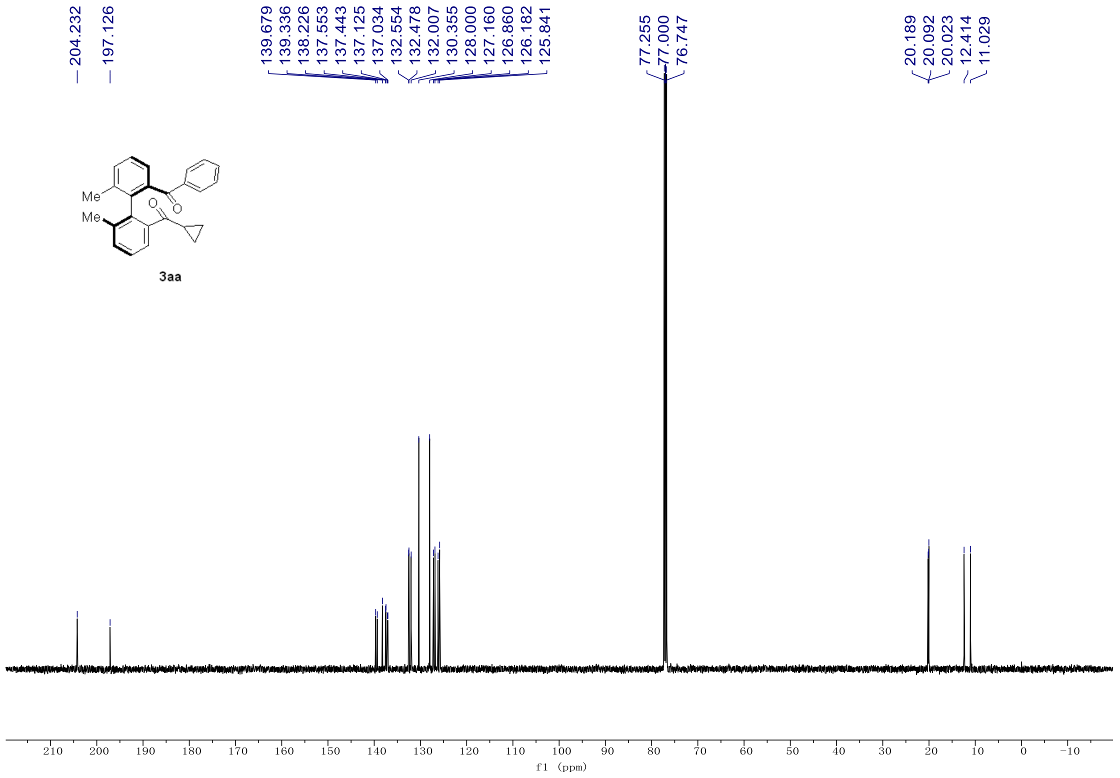
204.232
197.126
139.679 139.336 138.226 137.553 137.443 137.125 137.034 132.554 132.478 132.007 130.355 128.000 127.160 126.860 126.182 125.841
77.255 77.000 76.747
20.189 20.092 20.023 12.414 11.029
Me
Me
O
O
3aa
f1 (ppm)

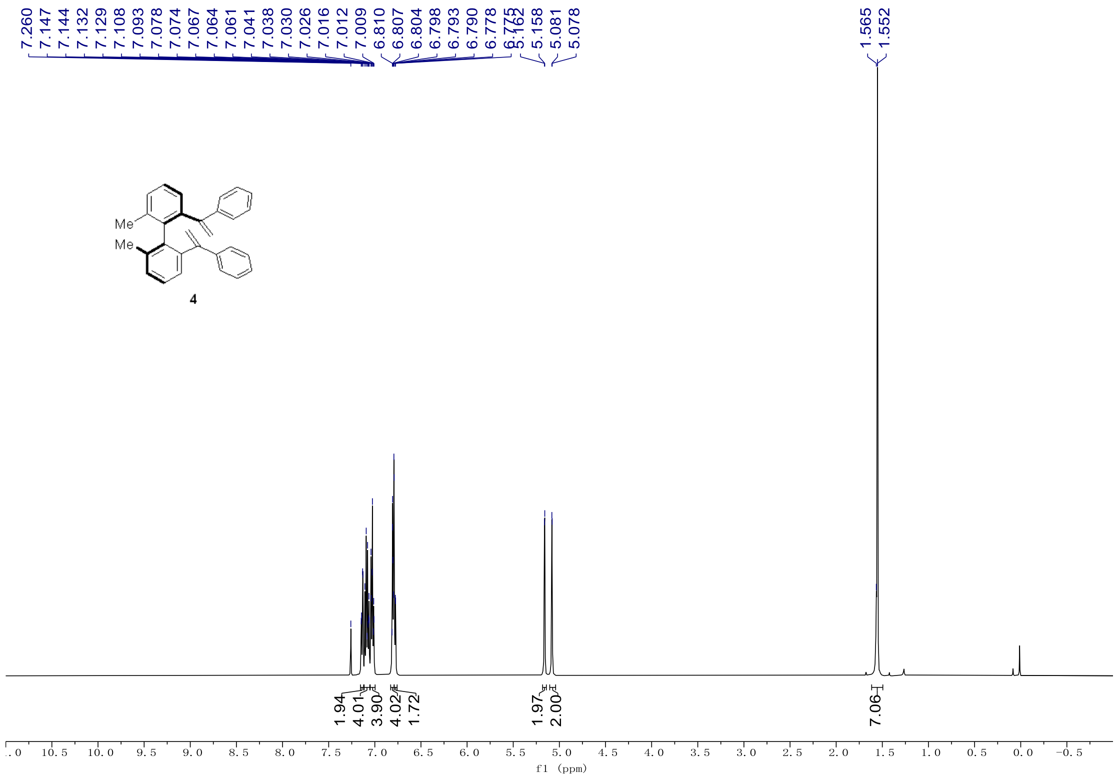

7.260
7.147
7.144
7.132
7.129
7.108
7.093
7.078
7.074
7.067
7.064
7.061
7.041
7.038
7.030
7.026
7.016
7.012
7.009
6.810
6.807
6.804
6.798
6.793
6.790
6.778
6.775
5.162
5.158
5.081
5.078
1.565
1.552
Me
Me
4
1.94
4.01
3.90
4.02
1.72
1.97
2.00
7.06
f1 (ppm)

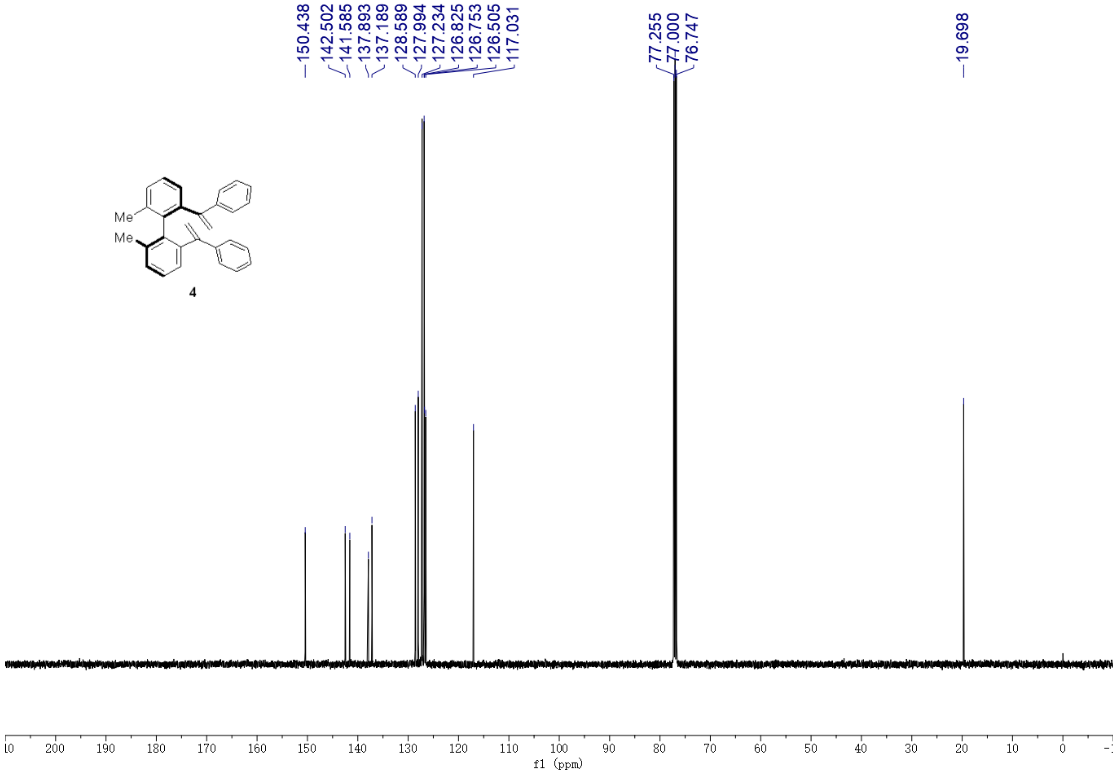

150.438
142.502
141.585
137.893
137.189
128.589
127.994
127.234
126.825
126.753
126.505
117.031
77.255
77.000
76.747
19.698
Me
Me
4
f1 (ppm)

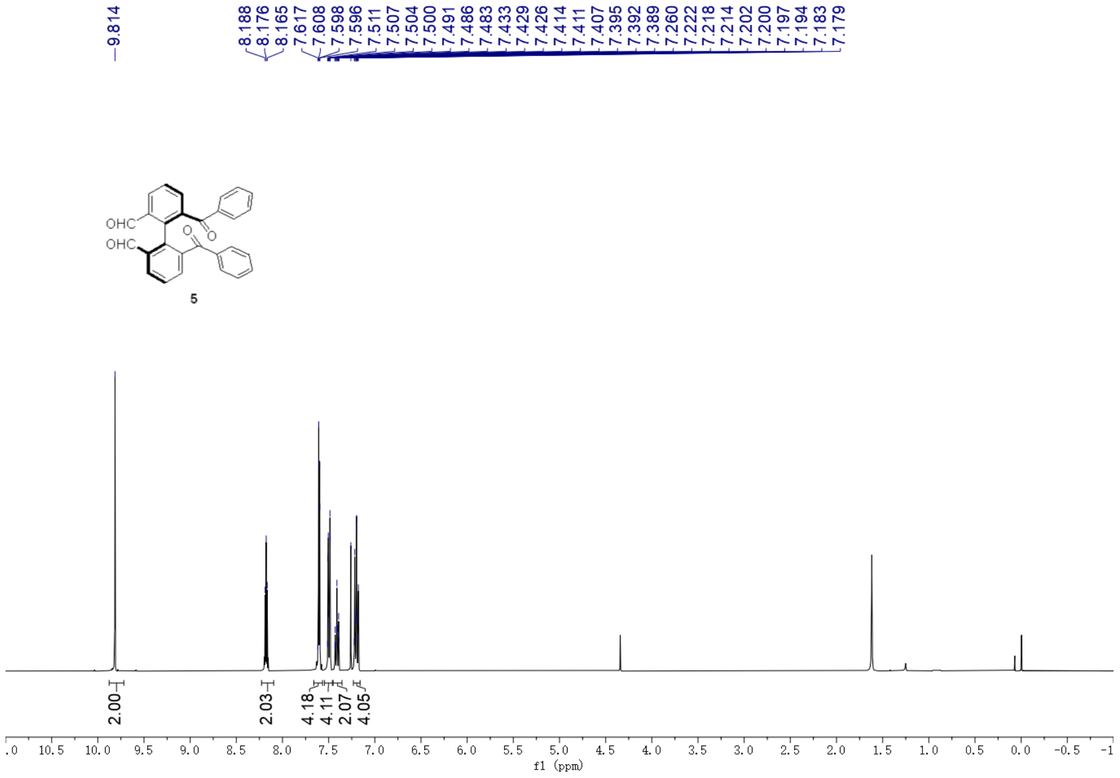

9.814
8.188
8.176
8.165
7.617
7.608
7.598
7.596
7.511
7.507
7.504
7.500
7.491
7.486
7.483
7.433
7.429
7.426
7.414
7.411
7.407
7.395
7.392
7.389
7.260
7.222
7.218
7.214
7.202
7.200
7.197
7.194
7.183
7.179
OHC
OHC
O
O
5
2.00
2.03
4.18
4.11
2.07
4.05
f1 (ppm)

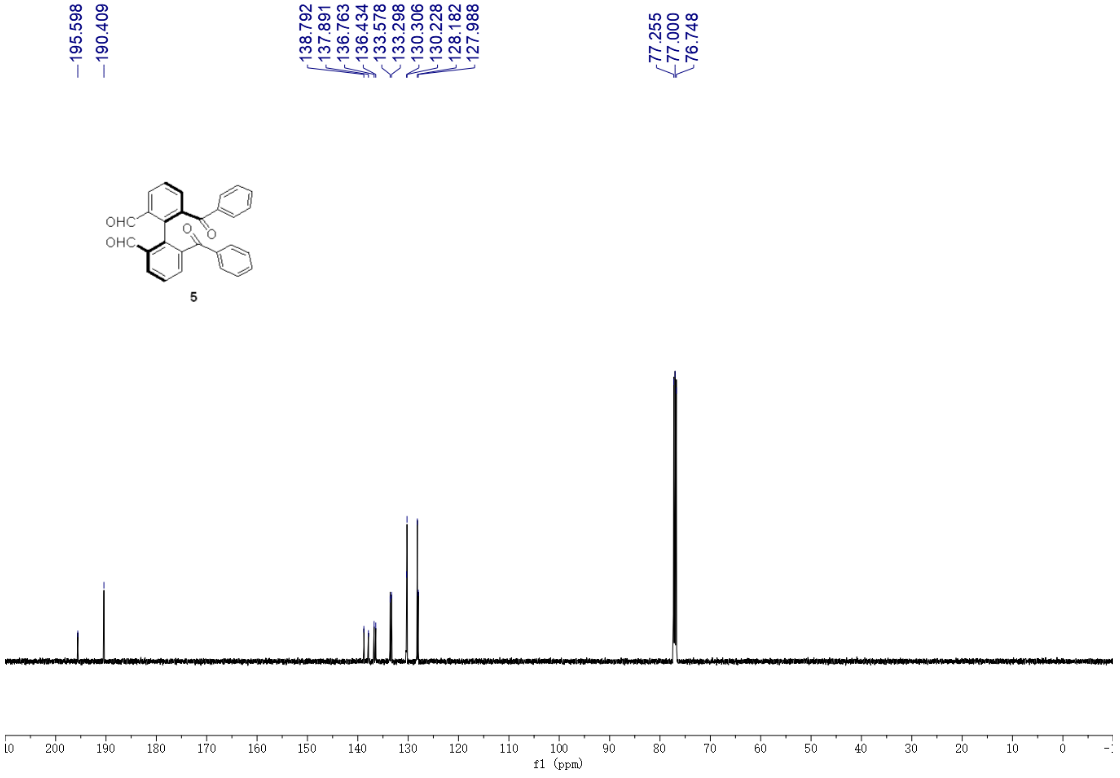

195.598
190.409
138.792
137.891
136.763
136.434
133.578
133.298
130.306
130.228
128.182
127.988
77.255
77.000
76.748
OHC
OHC
O
O
5
f1 (ppm)

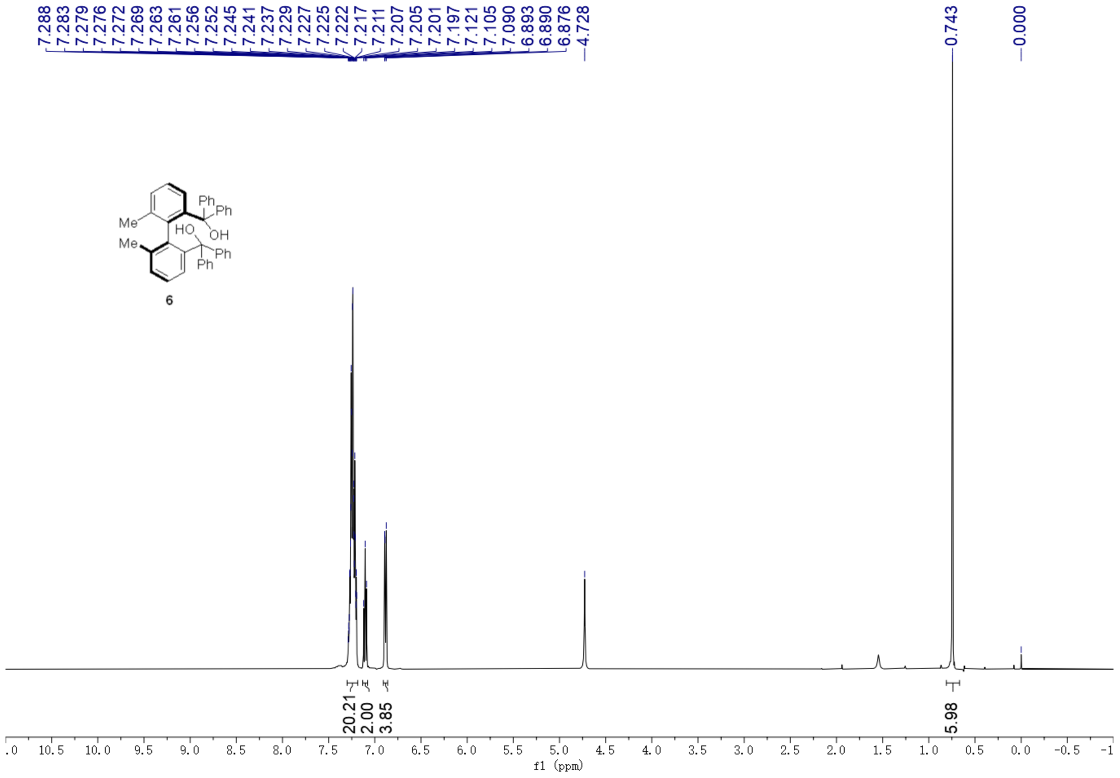
7.288
7.283
7.279
7.276
7.272
7.269
7.263
7.261
7.256
7.252
7.245
7.241
7.237
7.229
7.227
7.225
7.222
7.217
7.211
7.207
7.205
7.201
7.197
7.121
7.105
7.090
6.893
6.890
6.876
4.728
0.743
0.000
Me
Me
Ph
Ph
HO
OH
Ph
Ph
6
20.21
2.00
3.85
5.98
f1 (ppm)

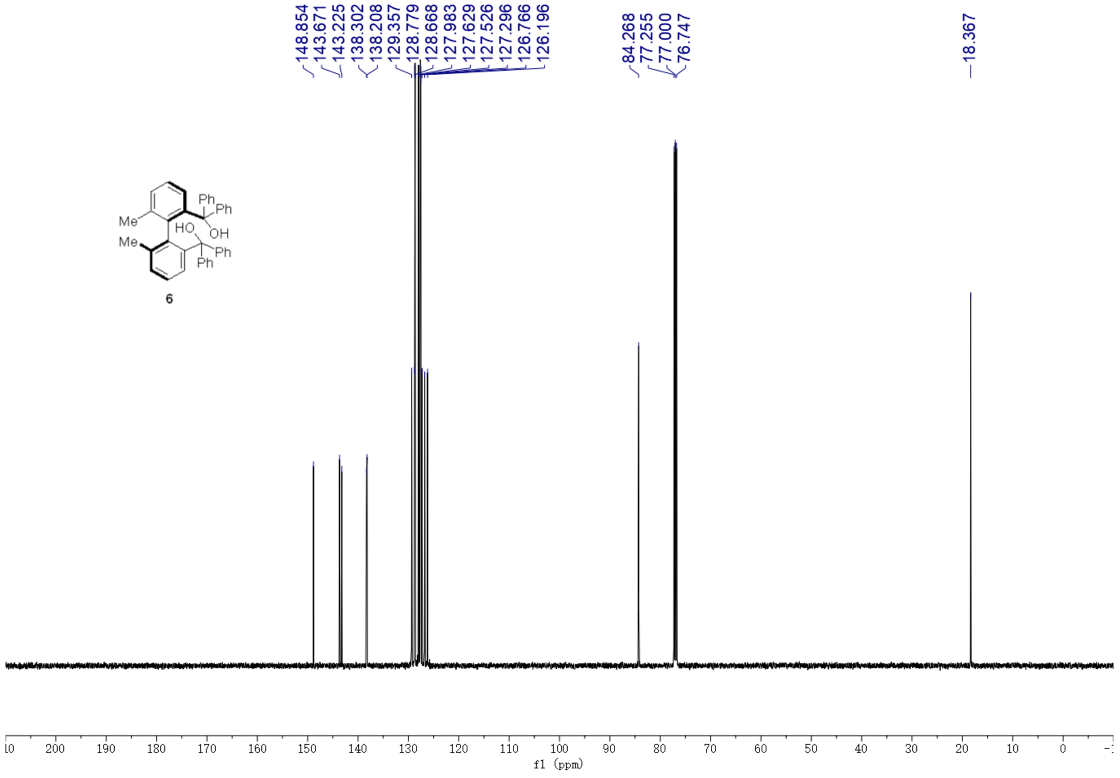
148.854
143.671
143.225
138.302
138.208
129.357
128.779
128.668
127.983
127.629
127.526
127.296
126.766
126.196
84.268
77.255
77.000
76.747
18.367
Me
Me
Ph
Ph
HO
OH
Ph
Ph
6
f1 (ppm)

Copies of HPLC Traces

<Chromatogram>

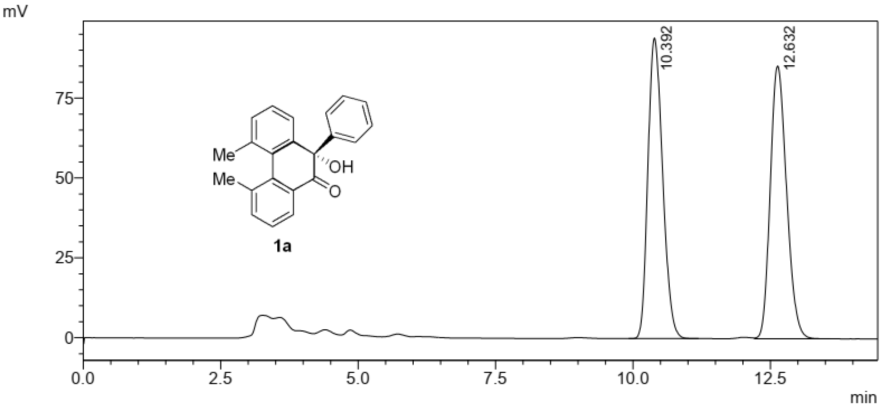

<Peak Table>

Detector A Channel 1 254nm

Peak#	Ret. Time	Area	Height	Conc.	Unit	Mark	Name
1	10.392	1754130	94046	49.825			
2	12.632	1766461	85386	50.175			
Total		3520591	179432				

<Chromatogram>

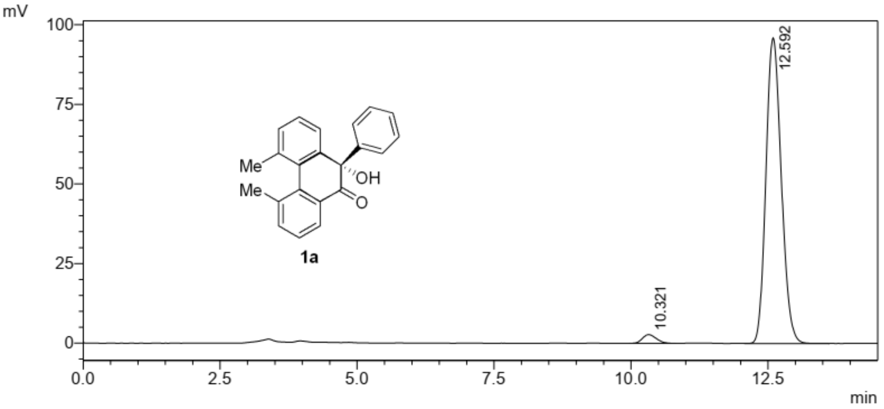

<Peak Table>

Detector A Channel 1 254nm

Peak#	Ret. Time	Area	Height	Conc.	Unit	Mark	Name
1	10.321	48969	2756	2.549			
2	12.592	1872157	95967	97.451			
Total		1921126	98724				

<Chromatogram>

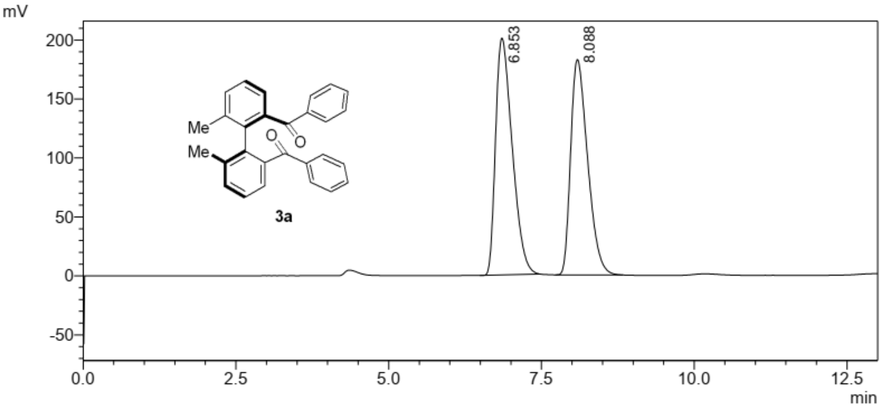

<Peak Table>

Detector A Channel 1 254nm

Peak#	Ret. Time	Area	Height	Conc.	Unit	Mark	Name
1	6.853	3803104	200752	51.830			
2	8.088	3534565	182606	48.170			
Total		7337669	383358				

<Chromatogram>

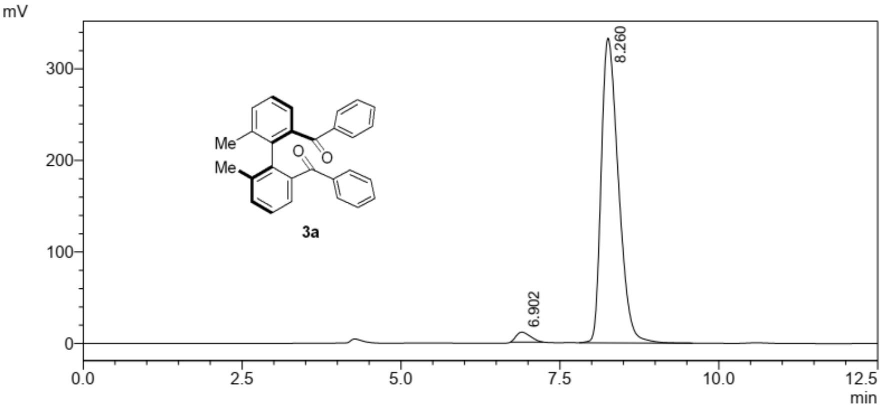

<Peak Table>

Detector A Channel 1 254nm

Peak#	Ret. Time	Area	Height	Conc.	Unit	Mark	Name
1	6.902	167066	10815	2.601			
2	8.260	6256364	332719	97.399			
Total		6423430	343534				

<Chromatogram>

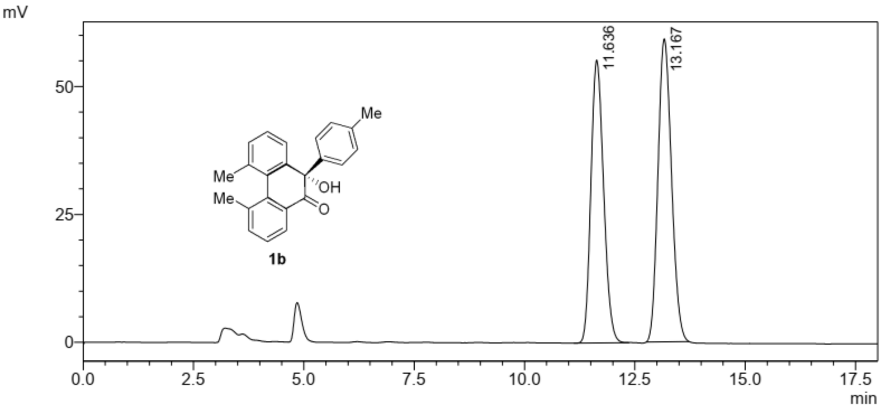

<Peak Table>

Detector A Channel 1 254nm

Peak#	Ret. Time	Area	Height	Conc.	Unit	Mark	Name
1	11.636	1105765	55245	46.703			
2	13.167	1261906	59198	53.297			
Total		2367671	114442				

<Chromatogram>

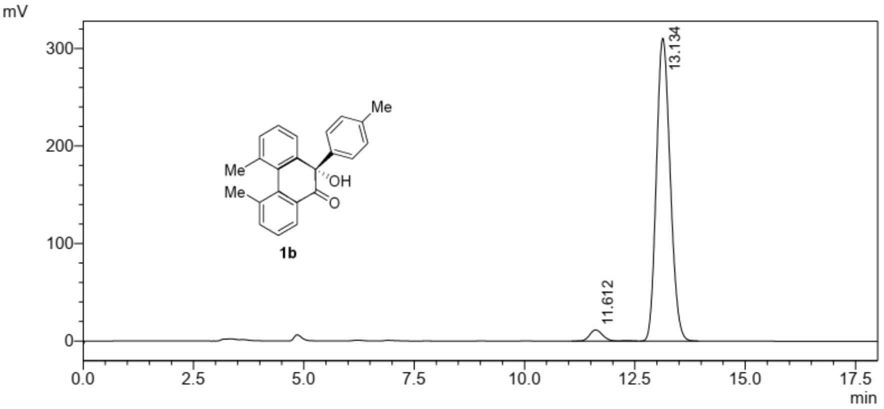

<Peak Table>

Detector A Channel 1 254nm

Peak#	Ret. Time	Area	Height	Conc.	Unit	Mark	Name
1	11.612	240825	11496	3.444			
2	13.134	6752127	310440	96.556			
Total		6992953	321936				

<Chromatogram>

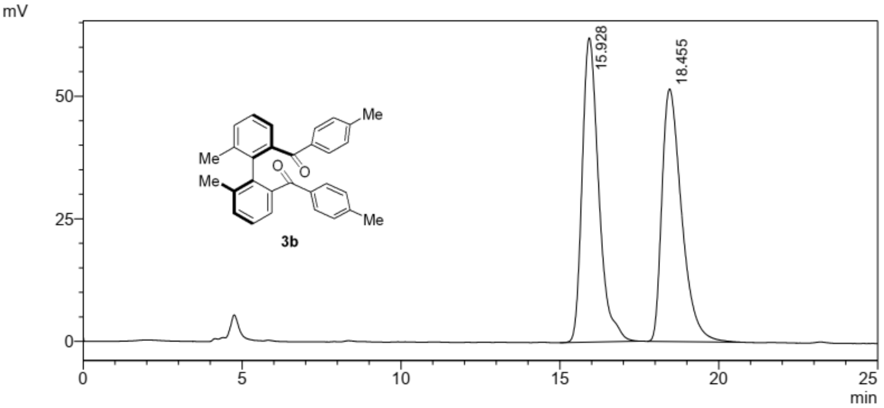

<Peak Table>

Detector A Channel 1 254nm

Peak#	Ret. Time	Area	Height	Conc.	Unit	Mark	Name
1	15.928	2252486	62062	50.354			
2	18.455	2220809	51504	49.646			
Total		4473296	113566				

<Chromatogram>

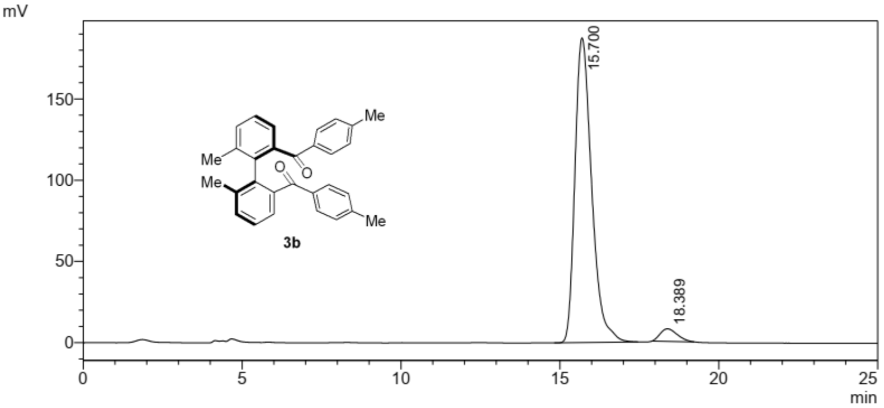

<Peak Table>

Detector A Channel 1 254nm

Peak#	Ret. Time	Area	Height	Conc.	Unit	Mark	Name
1	15.700	6866008	187479	95.961			
2	18.389	289000	7717	4.039			
Total		7155008	195195				

<Chromatogram>

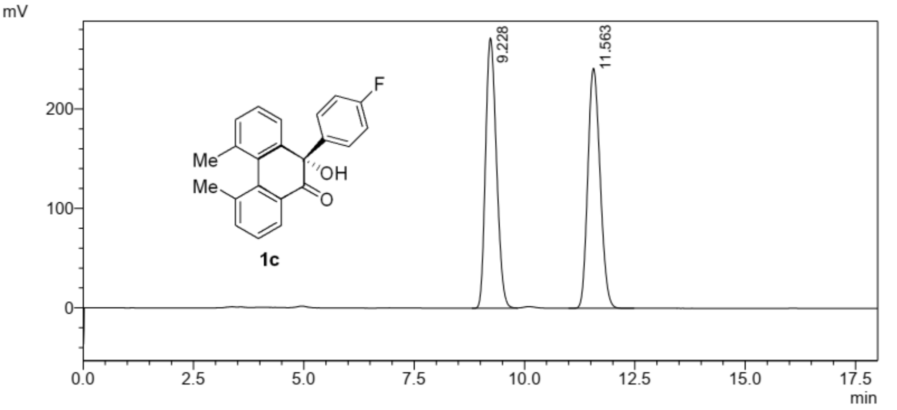

<Peak Table>

Detector A Channel 1 254nm

Peak#	Ret. Time	Area	Height	Conc.	Unit	Mark	Name
1	9.228	4668822	271447	50.000			
2	11.563	4668787	241110	50.000			
Total		9337609	512557				

<Chromatogram>

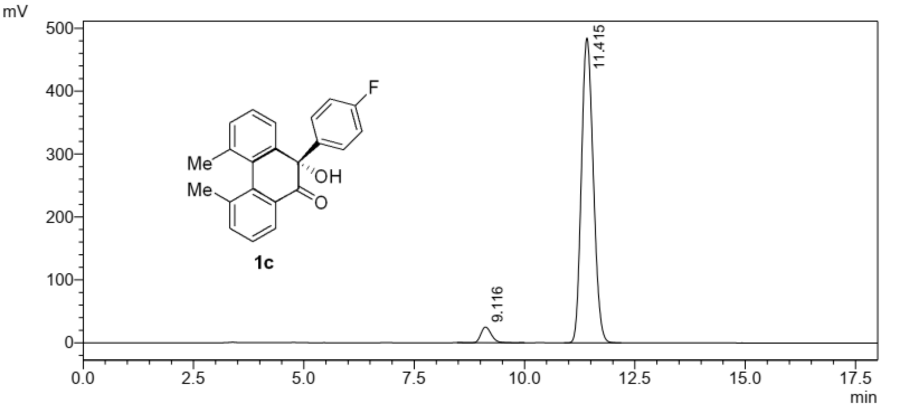

<Peak Table>

Detector A Channel 1 254nm

Peak#	Ret. Time	Area	Height	Conc.	Unit	Mark	Name
1	9.116	398965	24760	4.218			
2	11.415	9059023	484174	95.782			
Total		9457988	508934				

<Chromatogram>

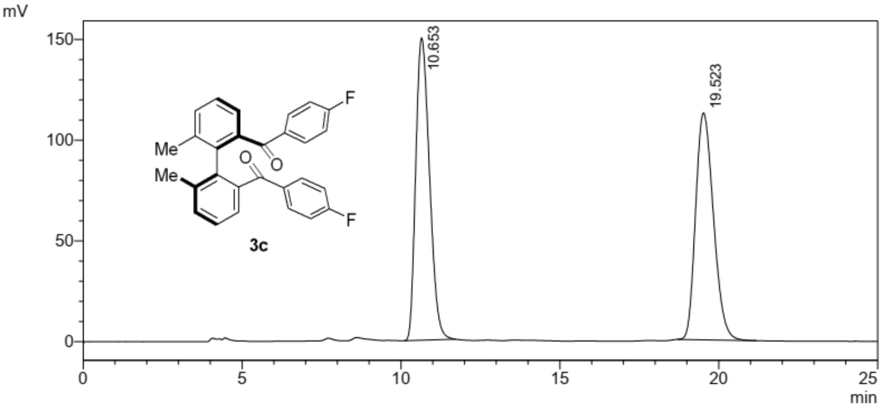

<Peak Table>

Detector A Channel 1 254nm

Peak#	Ret. Time	Area	Height	Conc.	Unit	Mark	Name
1	10.653	4509207	149993	50.649			
2	19.523	4393664	112539	49.351			
Total		8902871	262533				

<Chromatogram>

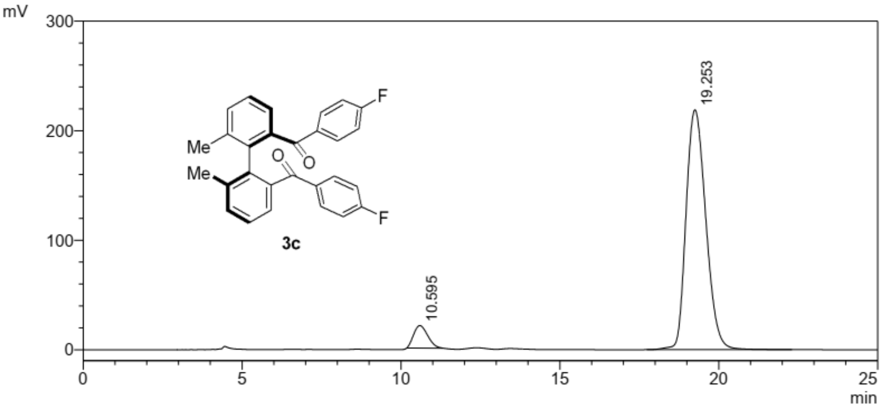

<Peak Table>

Detector A Channel 1 254nm

Peak#	Ret. Time	Area	Height	Conc.	Unit	Mark	Name
1	10.595	629564	20524	6.227			
2	19.253	9480863	219017	93.773			
Total		10110427	239541				

<Chromatogram>

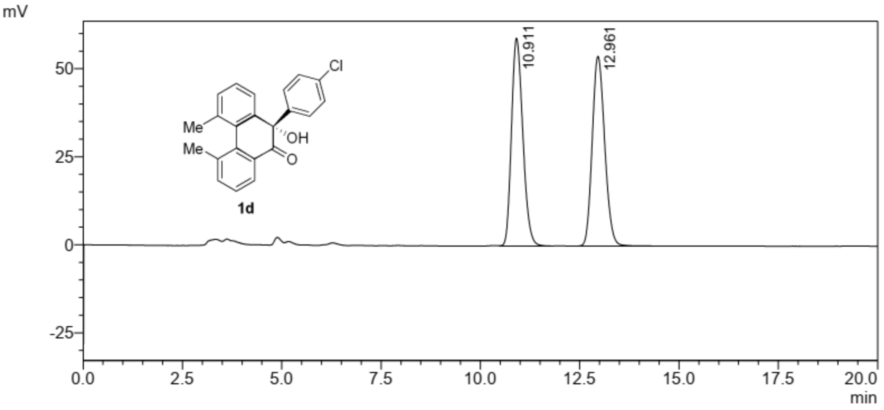

<Peak Table>

Detector A Channel 1 254nm

Peak#	Ret. Time	Area	Height	Conc.	Unit	Mark	Name
1	10.911	1172430	59004	49.886			
2	12.961	1177766	53905	50.114			
Total		2350196	112909				

<Chromatogram>

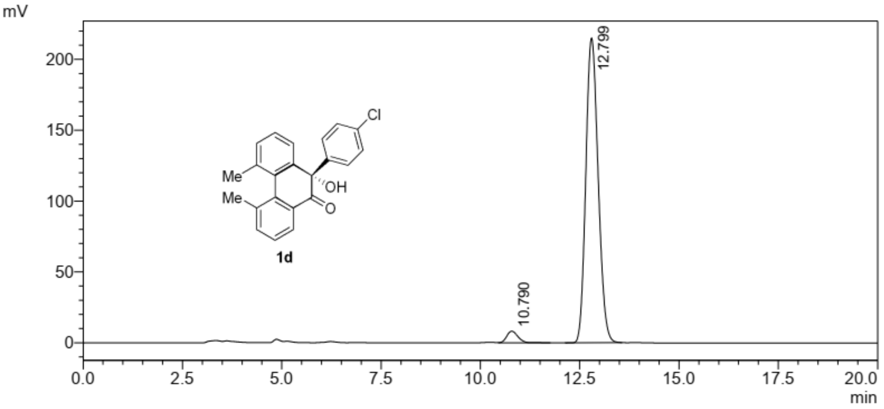

<Peak Table>

Detector A Channel 1 254nm

Peak#	Ret. Time	Area	Height	Conc.	Unit	Mark	Name
1	10.790	162224	8227	3.376			
2	12.799	4642815	215025	96.624			
Total		4805039	223252				

<Chromatogram>

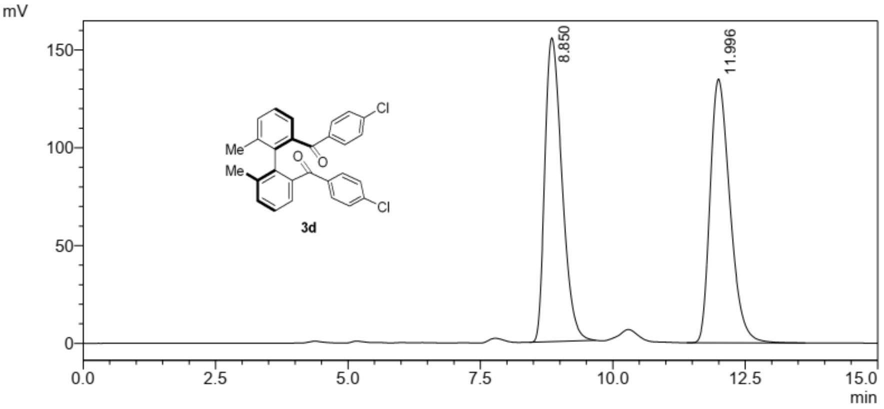

<Peak Table>

Detector A Channel 1 254nm

Peak#	Ret. Time	Area	Height	Conc.	Unit	Mark	Name
1	8.850	3493031	155297	49.861			
2	11.996	3512483	134849	50.139			
Total		7005514	290146				

<Chromatogram>

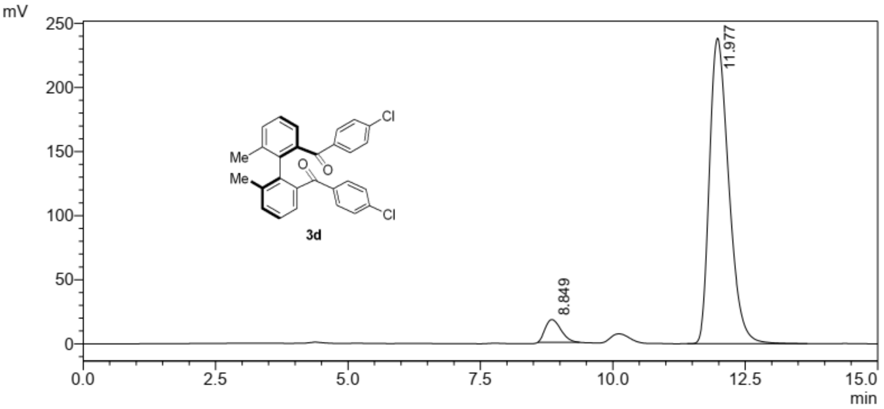

<Peak Table>

Detector A Channel 1 254nm

Peak#	Ret. Time	Area	Height	Conc.	Unit	Mark	Name
1	8.849	365378	17694	5.700			
2	11.977	6044466	238353	94.300			
Total		6409844	256047				

<Chromatogram>

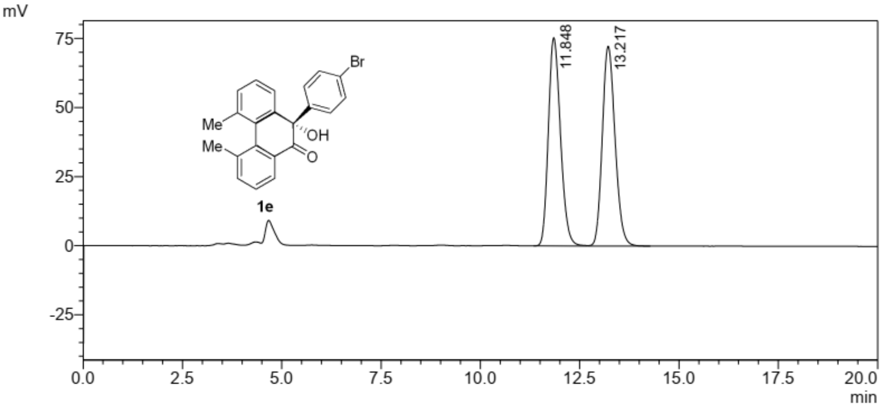

<Peak Table>

Detector A Channel 1 254nm

Peak#	Ret. Time	Area	Height	Conc.	Unit	Mark	Name
1	11.848	1600900	75307	49.779			
2	13.217	1615134	72252	50.221			
Total		3216034	147559				

<Chromatogram>

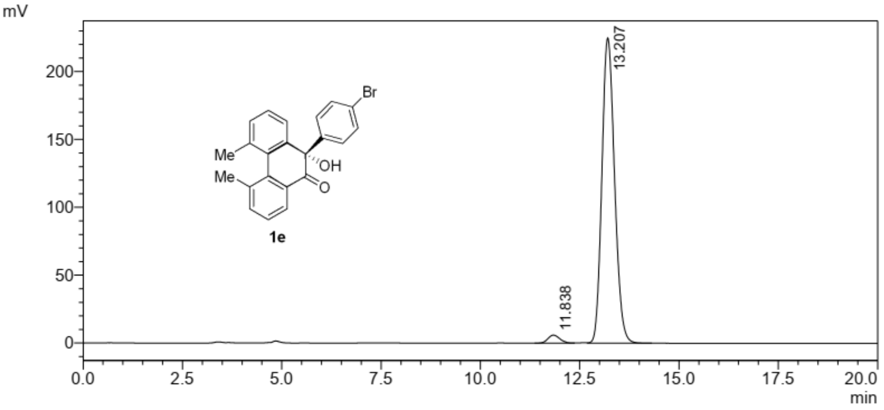

<Peak Table>

Detector A Channel 1 254nm

Peak#	Ret. Time	Area	Height	Conc.	Unit	Mark	Name
1	11.838	125548	5938	2.450			
2	13.207	4998206	224875	97.550			
Total		5123754	230813				

<Chromatogram>

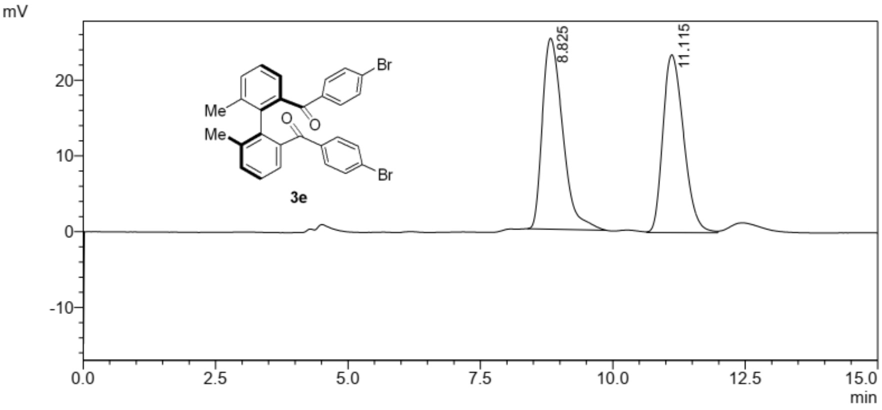

<Peak Table>

Detector A Channel 1 254nm

Peak#	Ret. Time	Area	Height	Conc.	Unit	Mark	Name
1	8.825	671956	25191	51.163			
2	11.115	641417	23470	48.837			
Total		1313374	48660				

<Chromatogram>

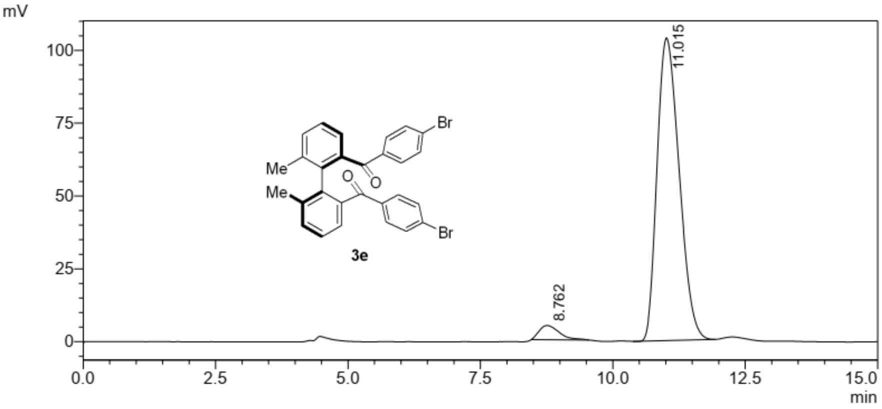

<Peak Table>

Detector A Channel 1 254nm

Peak#	Ret. Time	Area	Height	Conc.	Unit	Mark	Name
1	8.762	130854	4925	4.155			
2	11.015	3018602	103875	95.845			
Total		3149456	108800				

<Chromatogram>

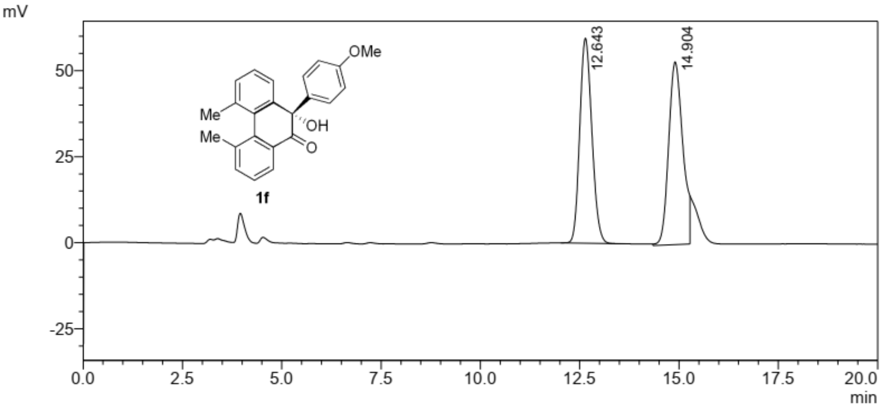

<Peak Table>

Detector A Channel 1 254nm

Peak#	Ret. Time	Area	Height	Conc.	Unit	Mark	Name
1	12.643	1262361	59555	49.007			
2	14.904	1313513	53106	50.993			
Total		2575874	112661				

<Chromatogram>

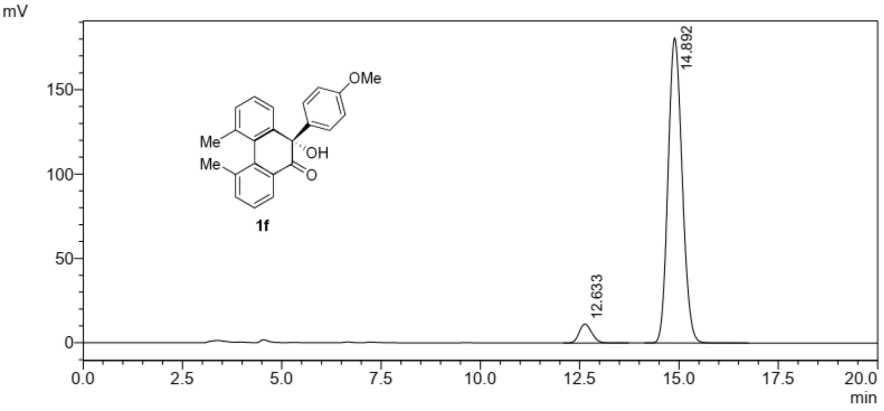

<Peak Table>

Detector A Channel 1 254nm

Peak#	Ret. Time	Area	Height	Conc.	Unit	Mark	Name
1	12.633	250777	11273	5.314			
2	14.892	4468186	180765	94.686			
Total		4718963	192038				

<Chromatogram>

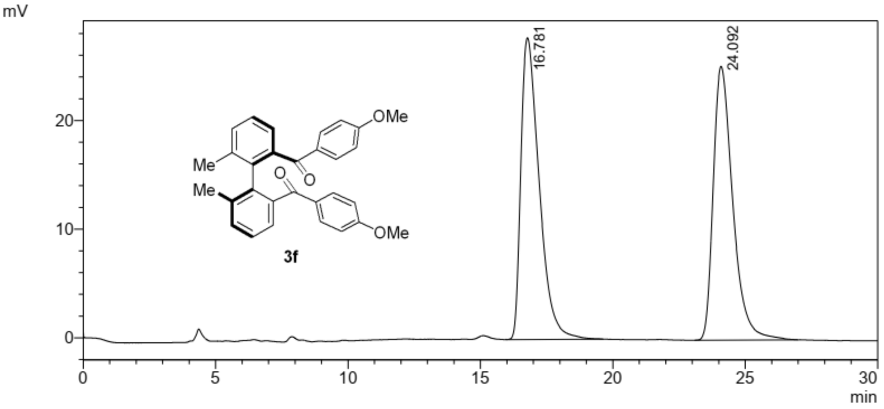

<Peak Table>

Detector A Channel 1 254nm

Peak#	Ret. Time	Area	Height	Conc.	Unit	Mark	Name
1	16.781	1337192	27758	50.841			
2	24.092	1292967	25203	49.159			
Total		2630159	52961				

<Chromatogram>

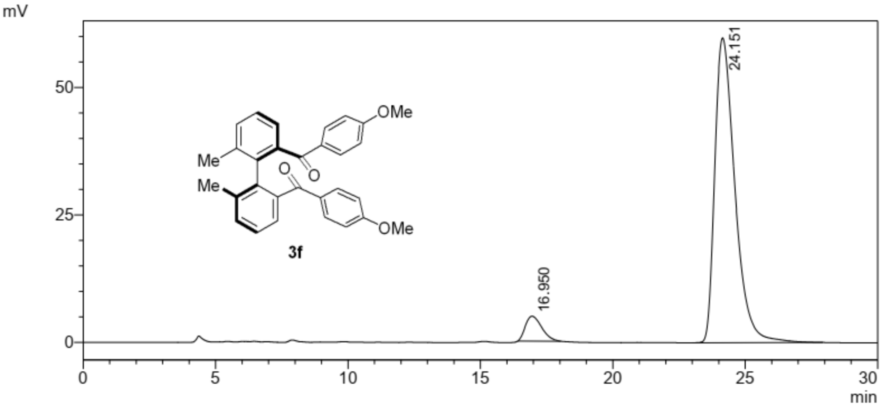

<Peak Table>

Detector A Channel 1 254nm

Peak#	Ret. Time	Area	Height	Conc.	Unit	Mark	Name
1	16.950	209817	4884	6.222			
2	24.151	3162426	59804	93.778			
Total		3372244	64688				

<Chromatogram>

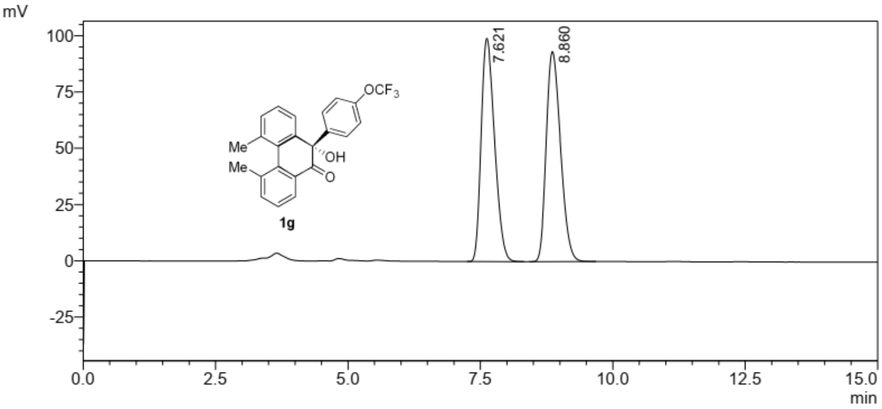

<Peak Table>

Detector A Channel 1 254nm

Peak#	Ret. Time	Area	Height	Conc.	Unit	Mark	Name
1	7.621	1746858	99200	49.944			
2	8.860	1750757	93304	50.056			
Total		3497615	192505				

<Chromatogram>

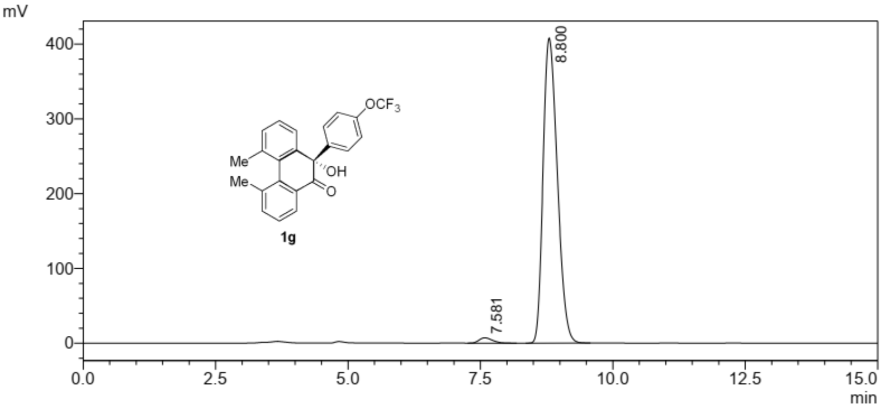

<Peak Table>

Detector A Channel 1 254nm

Peak#	Ret. Time	Area	Height	Conc.	Unit	Mark	Name
1	7.581	126385	7188	1.631			
2	8.800	7622506	407888	98.369			
Total		7748891	415076				

<Chromatogram>

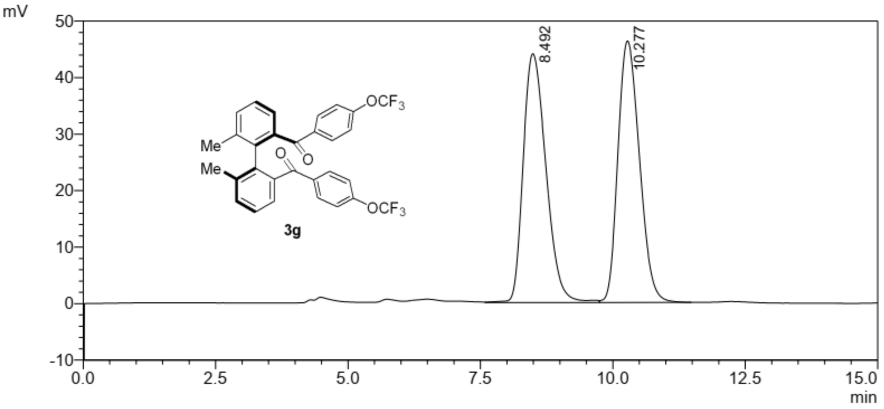

<Peak Table>

Detector A Channel 1 254nm

Peak#	Ret. Time	Area	Height	Conc.	Unit	Mark	Name
1	8.492	1321264	44059	50.256			
2	10.277	1307812	46273	49.744			
Total		2629075	90332				

<Chromatogram>

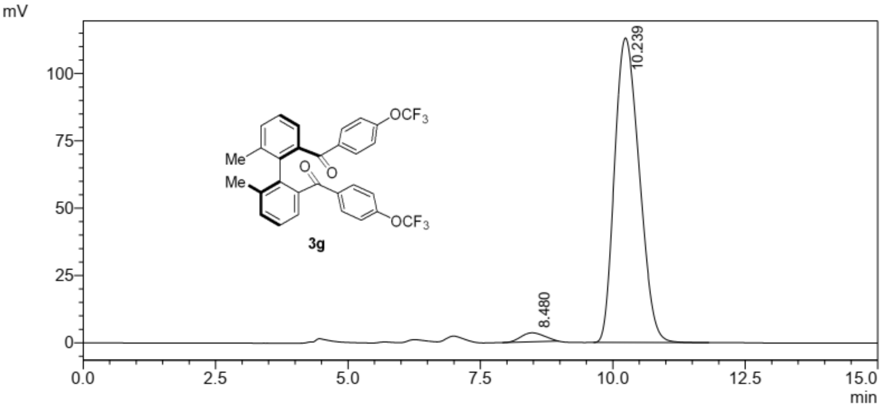

<Peak Table>

Detector A Channel 1 254nm

Peak#	Ret. Time	Area	Height	Conc.	Unit	Mark	Name
1	8.480	102273	3331	2.711			
2	10.239	3670377	113119	97.289			
Total		3772650	116449				

<Chromatogram>

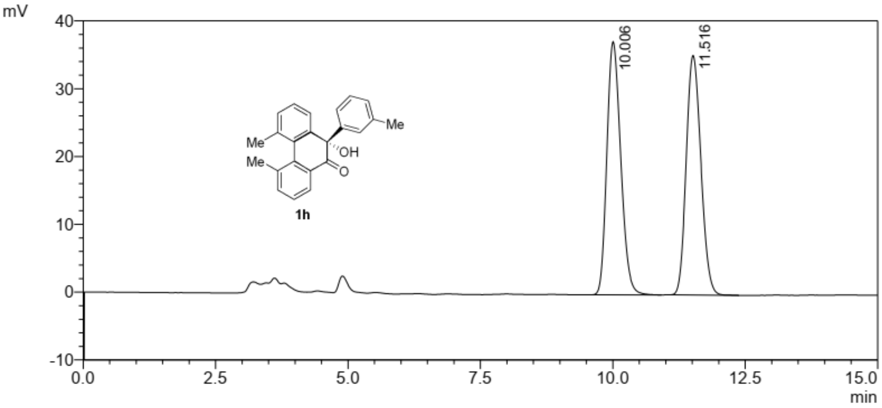

<Peak Table>

Detector A Channel 1 254nm

Peak#	Ret. Time	Area	Height	Conc.	Unit	Mark	Name
1	10.006	682204	37359	50.051			
2	11.516	680821	35314	49.949			
Total		1363025	72672				

<Chromatogram>

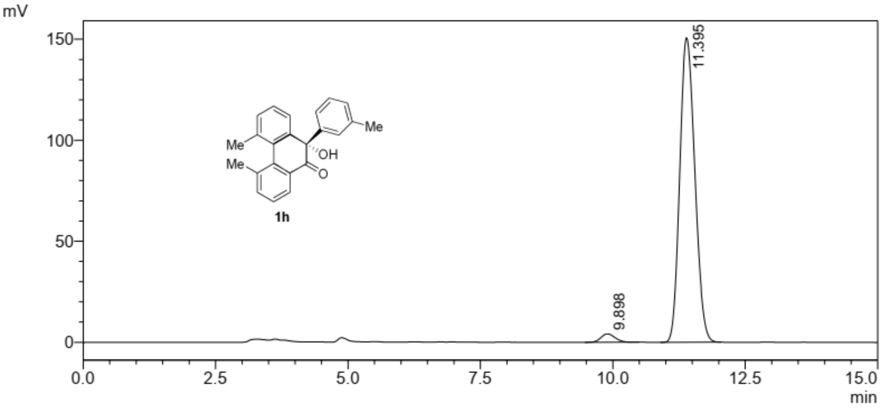

<Peak Table>

Detector A Channel 1 254nm

Peak#	Ret. Time	Area	Height	Conc.	Unit	Mark	Name
1	9.898	76376	4098	2.492			
2	11.395	2989049	150657	97.508			
Total		3065425	154755				

<Chromatogram>

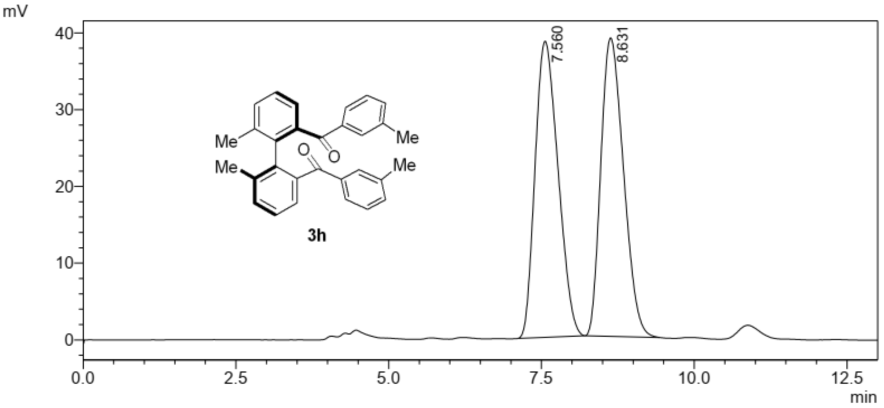

<Peak Table>

Detector A Channel 1 254nm

Peak#	Ret. Time	Area	Height	Conc.	Unit	Mark	Name
1	7.560	1007829	38581	50.119			
2	8.631	1003052	38875	49.881			
Total		2010881	77456				

<Chromatogram>

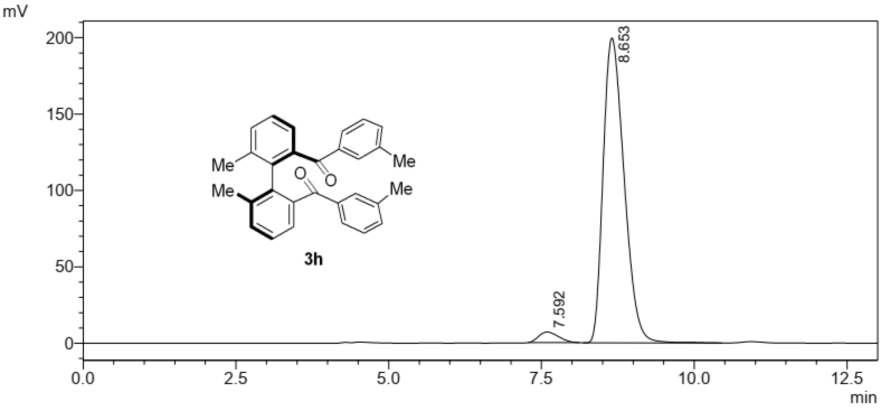

<Peak Table>

Detector A Channel 1 254nm

Peak#	Ret. Time	Area	Height	Conc.	Unit	Mark	Name
1	7.592	157233	6924	3.194			
2	8.653	4766237	199421	96.806			
Total		4923470	206346				

<Chromatogram>

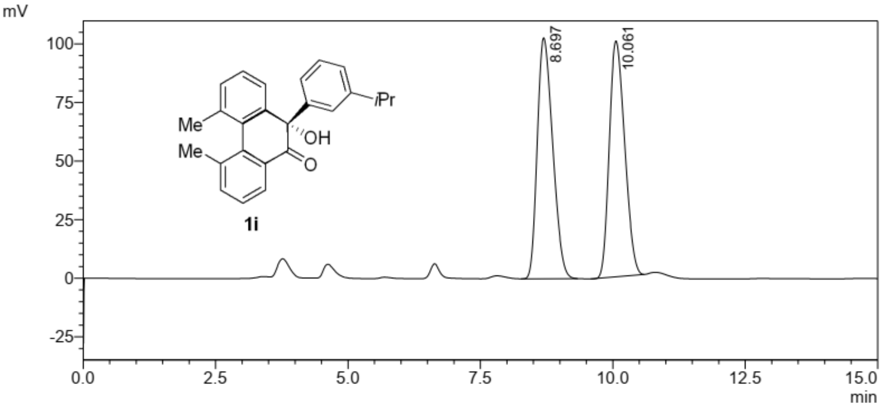

<Peak Table>

Detector A Channel 1 254nm

Peak#	Ret. Time	Area	Height	Conc.	Unit	Mark	Name
1	8.697	2084753	102767	50.601		M	
2	10.061	2035268	100638	49.399		M	
Total		4120022	203405				

<Chromatogram>

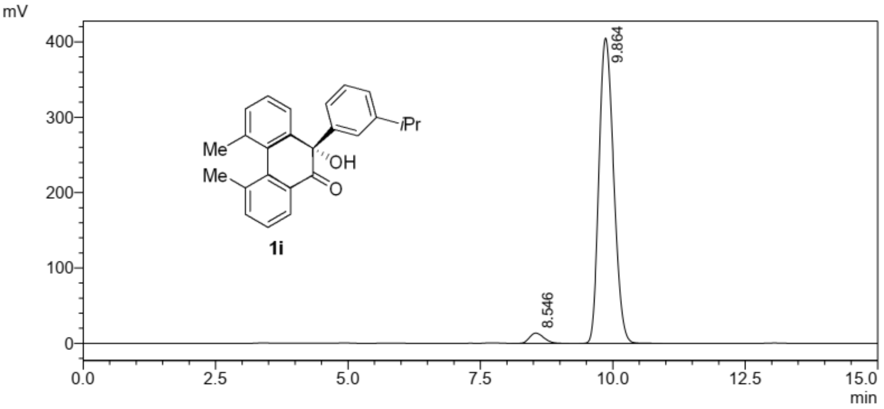

<Peak Table>

Detector A Channel 1 254nm

Peak#	Ret. Time	Area	Height	Conc.	Unit	Mark	Name
1	8.546	255373	13569	3.170		M	
2	9.864	7801635	404920	96.830		M	
Total		8057009	418489				

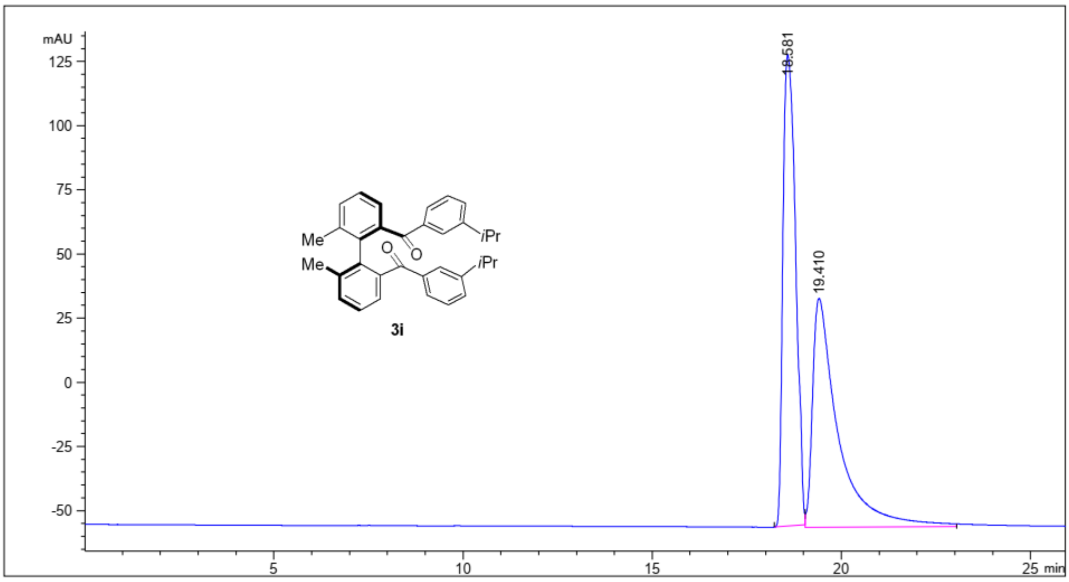

Signal 1: DAD1 A, Sig=254,4 Ref=360,100

Peak #	RetTime [min]	Type	Width [min]	Area [mAU*s]	Height [mAU]	Area %
1	18.581	BV	0.3760	4249.53760	183.62791	50.1707
2	19.410	MM	0.7879	4220.61426	89.28460	49.8293

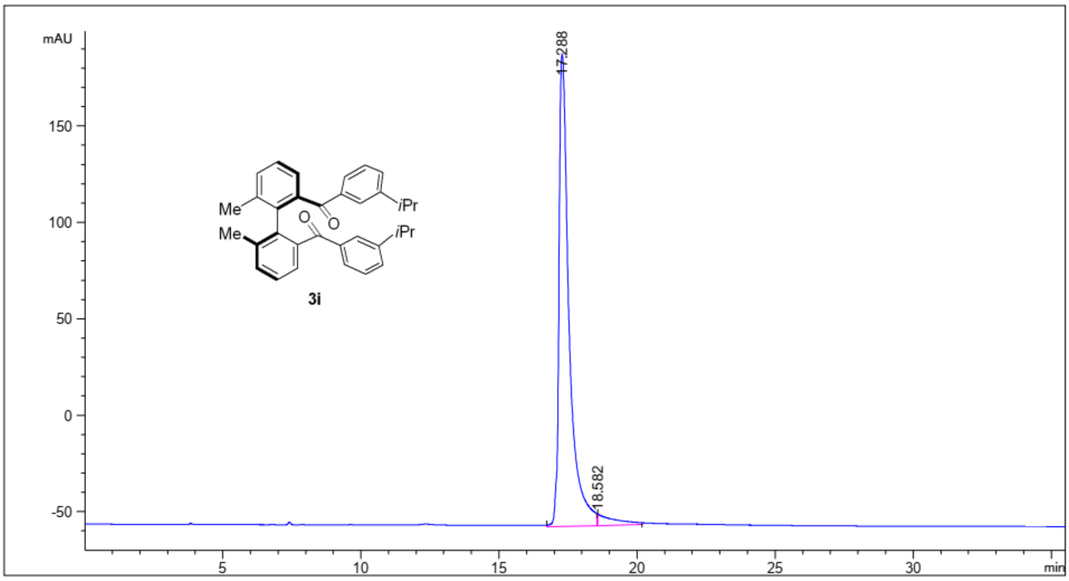

Signal 1: DAD1 A, Sig=254,4 Ref=360,100

Peak #	RetTime [min]	Type	Width [min]	Area [mAU*s]	Height [mAU]	Area %
1	17.288	MM	0.4252	6245.22021	244.77715	96.0970
2	18.582	MM	0.5013	253.65062	5.92736	3.9030

<Chromatogram>

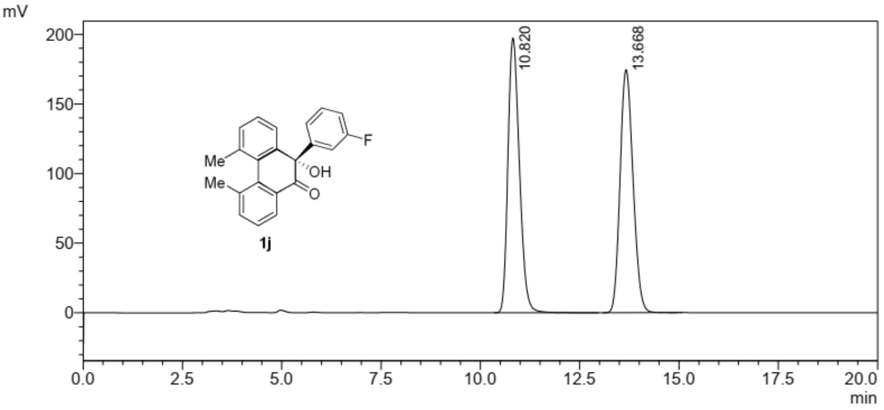

<Peak Table>

Detector A Channel 1 254nm

Peak#	Ret. Time	Area	Height	Conc.	Unit	Mark	Name
1	10.820	3960052	197146	49.948			
2	13.668	3968298	174478	50.052			
Total		7928349	371625				

<Chromatogram>

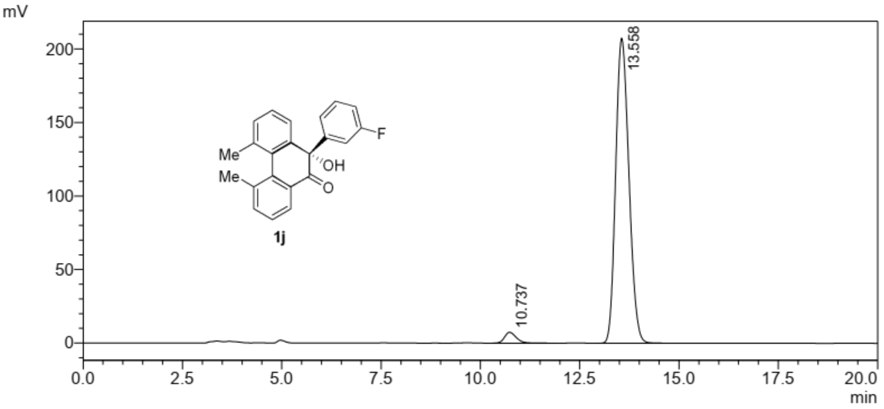

<Peak Table>

Detector A Channel 1 254nm

Peak#	Ret. Time	Area	Height	Conc.	Unit	Mark	Name
1	10.737	154393	7359	3.109			
2	13.558	4812187	207352	96.891			
Total		4966580	214711				

<Chromatogram>

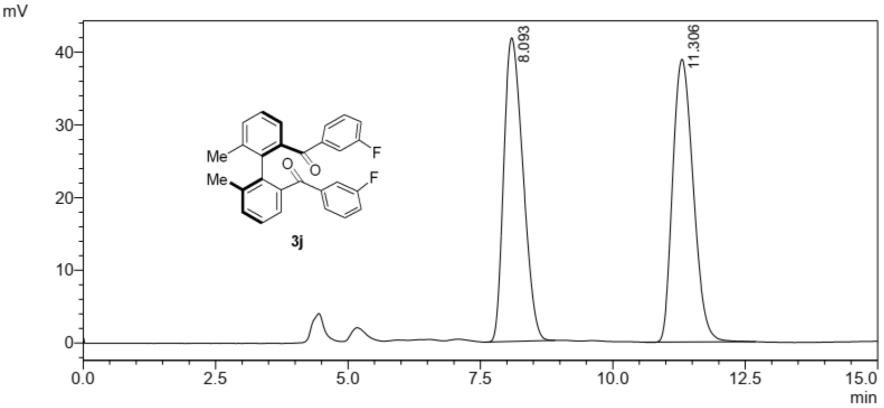

<Peak Table>

Detector A Channel 1 254nm

Peak#	Ret. Time	Area	Height	Conc.	Unit	Mark	Name
1	8.093	1052522	41726	49.970			
2	11.306	1053789	38884	50.030			
Total		2106311	80610				

<Chromatogram>

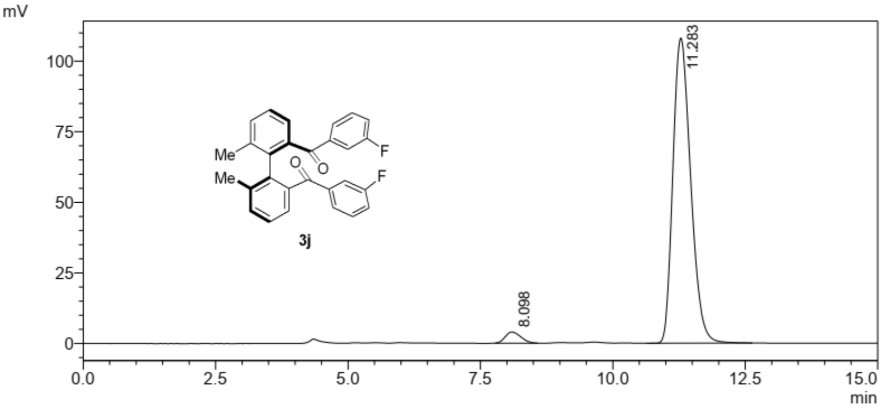

<Peak Table>

Detector A Channel 1 254nm

Peak#	Ret. Time	Area	Height	Conc.	Unit	Mark	Name
1	8.098	80999	3932	3.108			
2	11.283	2524892	108028	96.892			
Total		2605891	111960				

<Chromatogram>

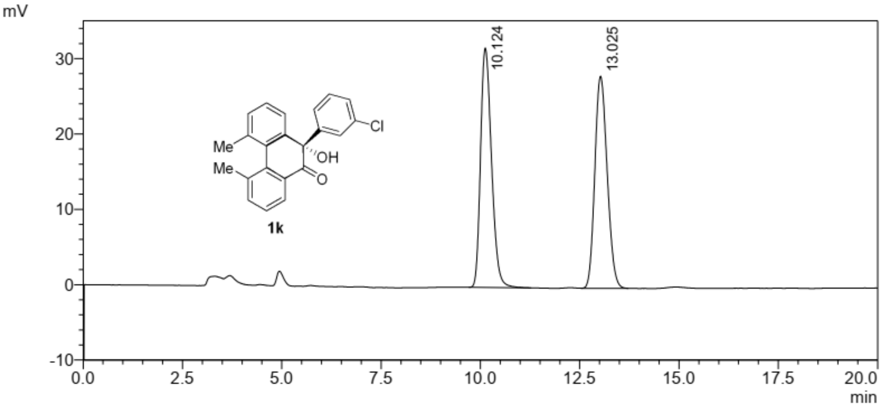

<Peak Table>

Detector A Channel 1 254nm

Peak#	Ret. Time	Area	Height	Conc.	Unit	Mark	Name
1	10.124	609553	31747	50.053			
2	13.025	608274	28126	49.947			
Total		1217827	59873				

<Chromatogram>

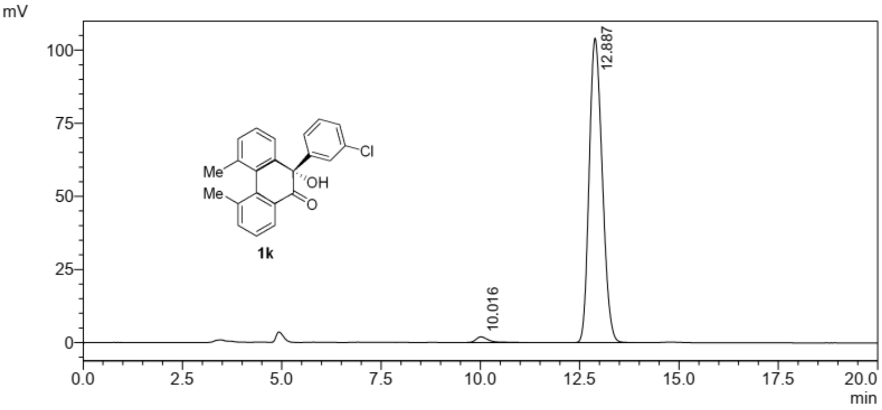

<Peak Table>

Detector A Channel 1 254nm

Peak#	Ret. Time	Area	Height	Conc.	Unit	Mark	Name
1	10.016	45009	1972	1.854			
2	12.887	2383321	104101	98.146			
Total		2428330	106074				

<Chromatogram>

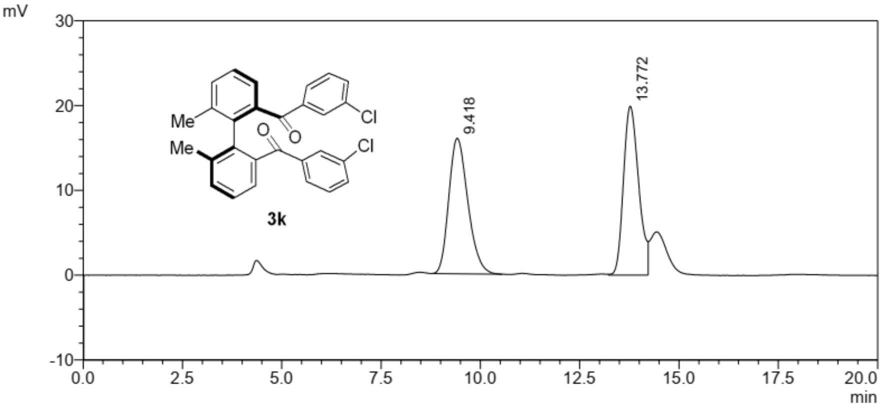

<Peak Table>

Detector A Channel 1 254nm

Peak#	Ret. Time	Area	Height	Conc.	Unit	Mark	Name
1	9.418	534012	15973	50.128			
2	13.772	531291	19936	49.872			
Total		1065303	35909				

<Chromatogram>

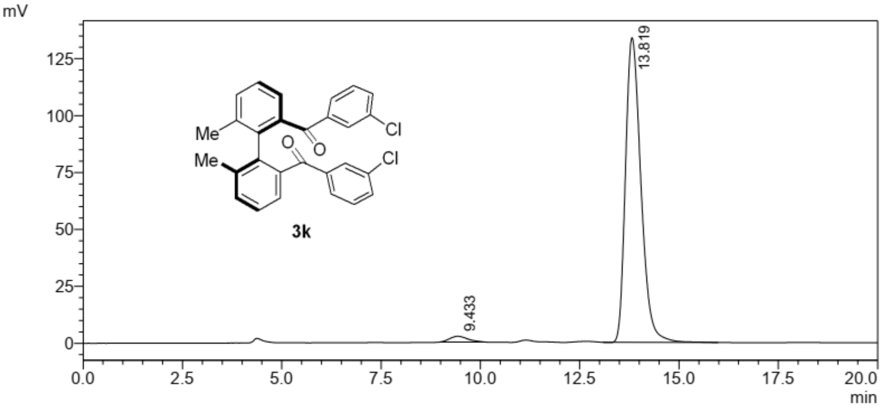

<Peak Table>

Detector A Channel 1 254nm

Peak#	Ret. Time	Area	Height	Conc.	Unit	Mark	Name
1	9.433	78280	2497	2.118			
2	13.819	3617034	133730	97.882			
Total		3695314	136227				

<Chromatogram>

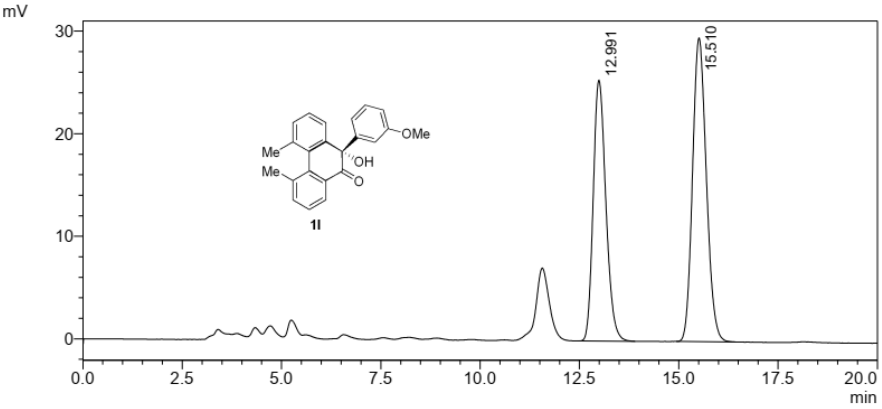

<Peak Table>

Detector A Channel 1 254nm

Peak#	Ret. Time	Area	Height	Conc.	Unit	Mark	Name
1	12.991	564901	25431	44.072			
2	15.510	716856	29606	55.928			
Total		1281757	55037				

<Chromatogram>

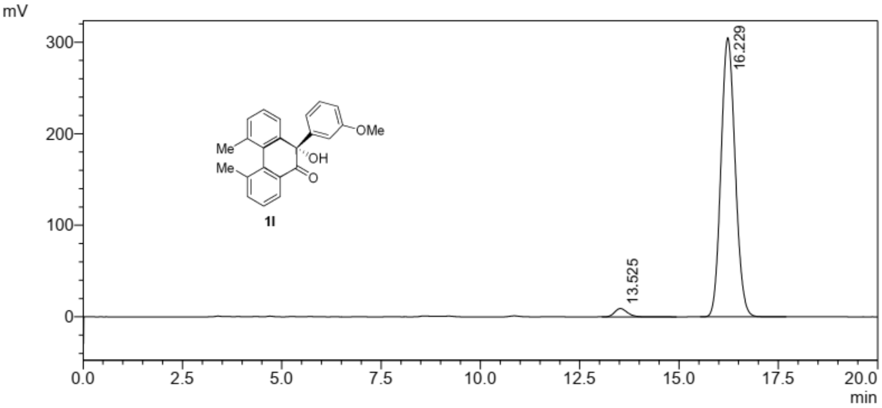

<Peak Table>

Detector A Channel 1 254nm

Peak#	Ret. Time	Area	Height	Conc.	Unit	Mark	Name
1	13.525	207299	9140	2.664			
2	16.229	7573810	304696	97.336			
Total		7781108	313836				

<Chromatogram>

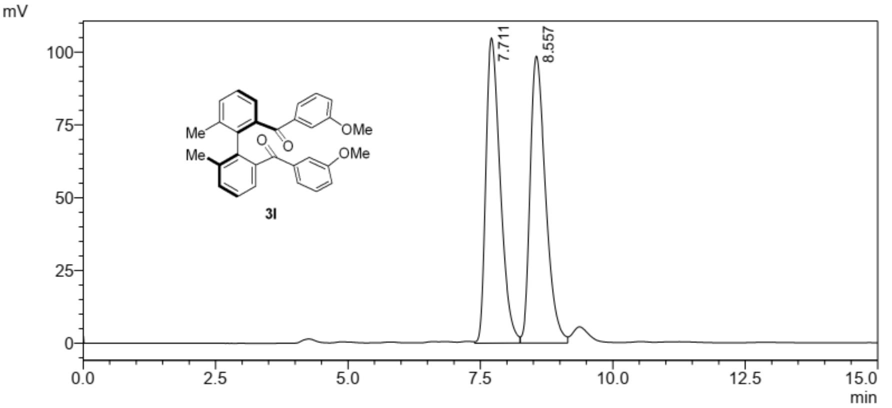

<Peak Table>

Detector A Channel 1 254nm

Peak#	Ret. Time	Area	Height	Conc.	Unit	Mark	Name
1	7.711	1976851	104771	49.711			
2	8.557	1999828	98579	50.289			
Total		3976679	203349				

<Chromatogram>

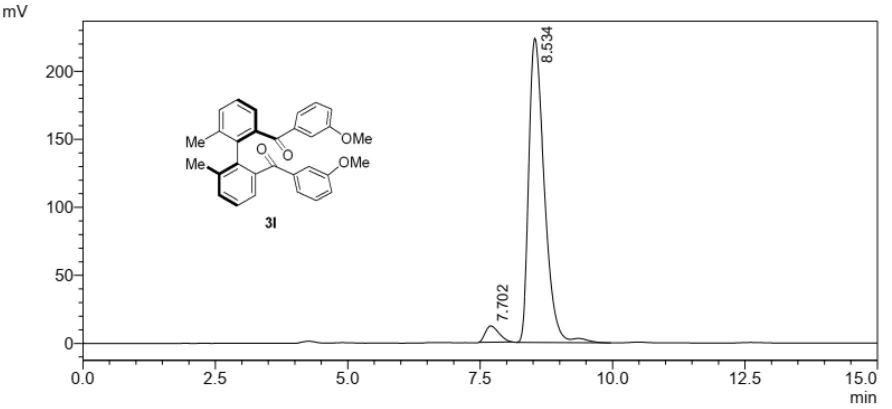

<Peak Table>

Detector A Channel 1 254nm

Peak#	Ret. Time	Area	Height	Conc.	Unit	Mark	Name
1	7.702	212745	11968	4.445			
2	8.534	4573331	223531	95.555			
Total		4786076	235498				

<Chromatogram>

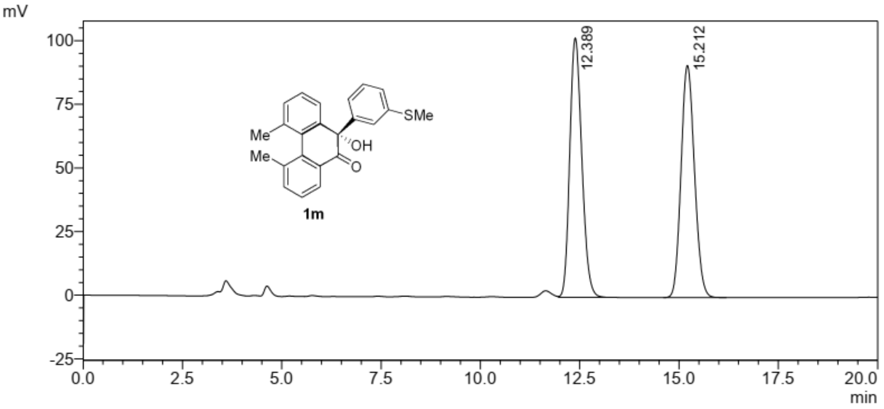

<Peak Table>

Detector A Channel 1 254nm

Peak#	Ret. Time	Area	Height	Conc.	Unit	Mark	Name
1	12.389	2147809	101893	50.013			
2	15.212	2146668	91195	49.987			
Total		4294478	193088				

<Chromatogram>

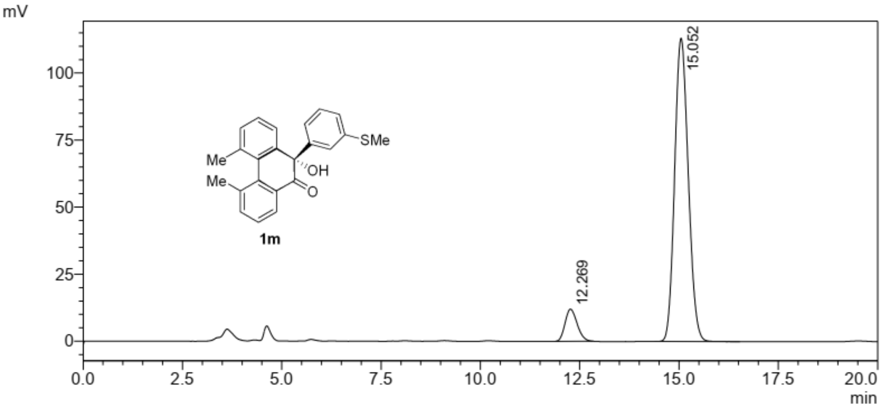

<Peak Table>

Detector A Channel 1 254nm

Peak#	Ret. Time	Area	Height	Conc.	Unit	Mark	Name
1	12.269	254278	11996	8.621			
2	15.052	2695405	113035	91.379			
Total		2949683	125031				

<Chromatogram>

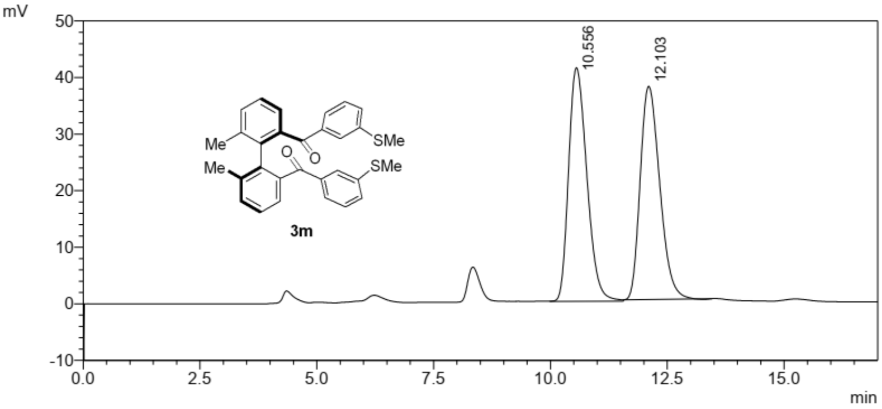

<Peak Table>

Detector A Channel 1 254nm

Peak#	Ret. Time	Area	Height	Conc.	Unit	Mark	Name
1	10.556	1123543	41305	50.668			
2	12.103	1093925	37682	49.332			
Total		2217468	78987				

<Chromatogram>

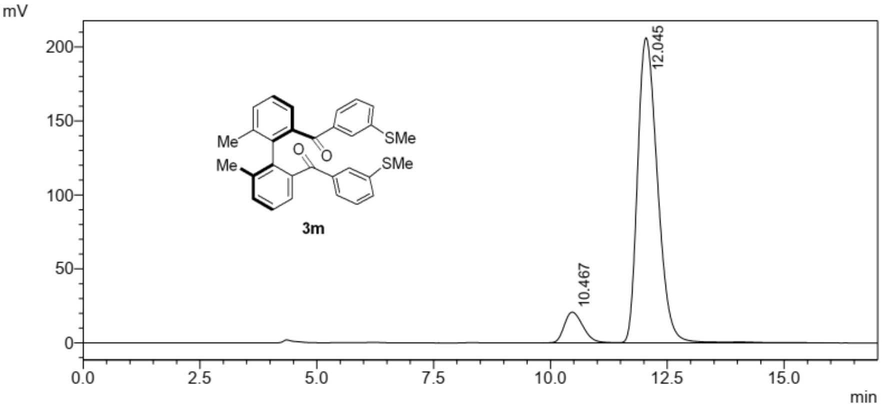

<Peak Table>

Detector A Channel 1 254nm

Peak#	Ret. Time	Area	Height	Conc.	Unit	Mark	Name
1	10.467	579641	20809	8.651			
2	12.045	6120293	206172	91.349			
Total		6699934	226981				

<Chromatogram>

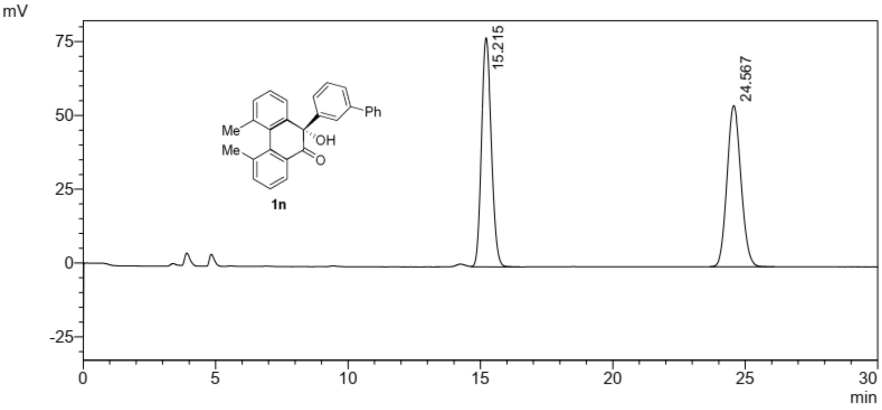

<Peak Table>

Detector A Channel 1 254nm

Peak#	Ret. Time	Area	Height	Conc.	Unit	Mark	Name
1	15.215	2001710	77543	50.002			
2	24.567	2001520	54595	49.998			
Total		4003230	132138				

<Chromatogram>

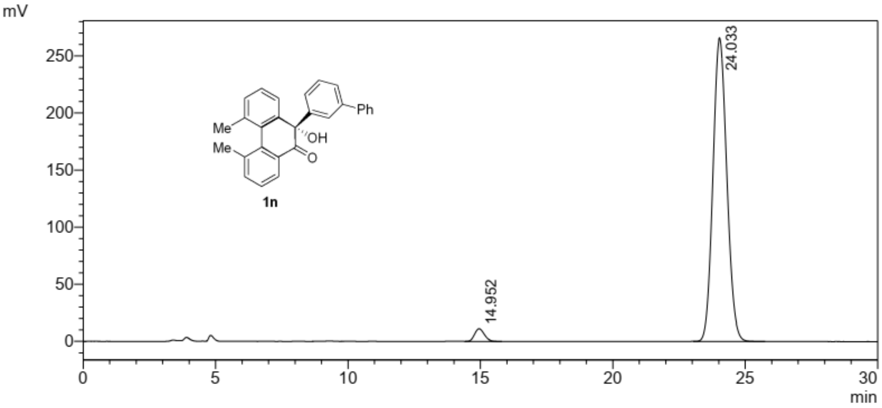

<Peak Table>

Detector A Channel 1 254nm

Peak#	Ret. Time	Area	Height	Conc.	Unit	Mark	Name
1	14.952	285263	11117	2.849			
2	24.033	9726831	265960	97.151			
Total		10012094	277078				

<Chromatogram>

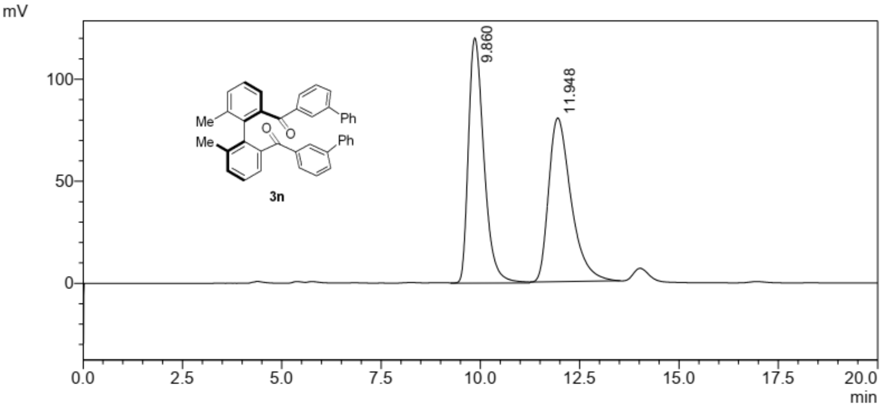

<Peak Table>

Detector A Channel 1 254nm

Peak#	Ret. Time	Area	Height	Conc.	Unit	Mark	Name
1	9.860	3268242	120107	50.921			
2	11.948	3149957	80177	49.079			
Total		6418198	200283				

<Chromatogram>

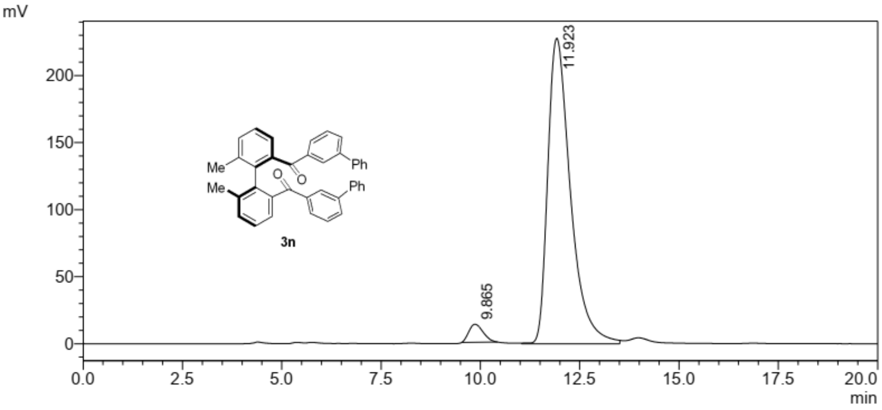

<Peak Table>

Detector A Channel 1 254nm

Peak#	Ret. Time	Area	Height	Conc.	Unit	Mark	Name
1	9.865	338448	13482	3.603			
2	11.923	9055898	227852	96.397			
Total		9394346	241334				

<Chromatogram>

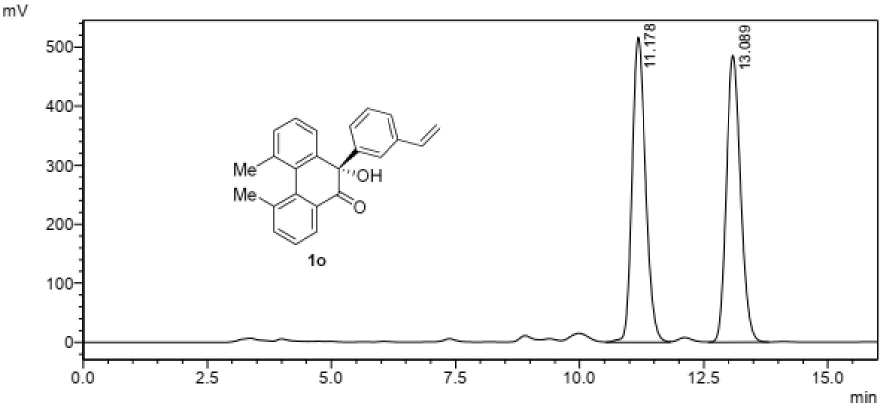

<Peak Table>

Detector A Channel 1 254nm

Peak#	Ret. Time	Area	Height	Conc.	Unit	Mark	Name
1	11.178	9914469	515863	50.201			
2	13.089	9835225	485681	49.799			
Total		19749694	1001543				

<Chromatogram>

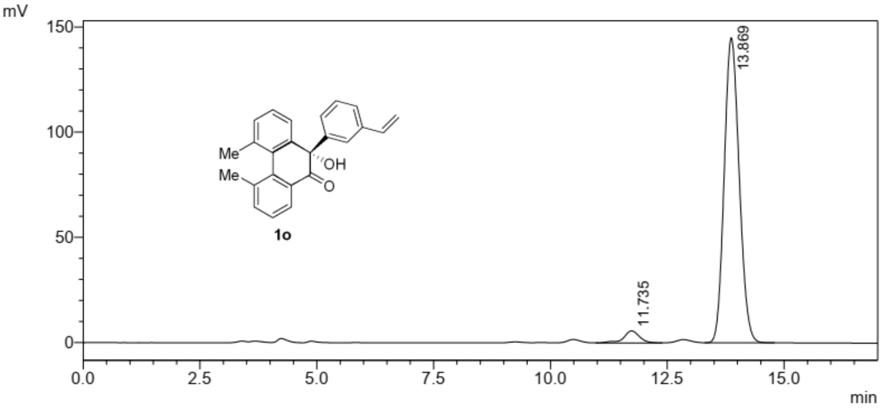

<Peak Table>

Detector A Channel 1 254nm

Peak#	Ret. Time	Area	Height	Conc.	Unit	Mark	Name
1	11.735	137297	5721	4.065			
2	13.869	3240567	144936	95.935			
Total		3377864	150657				

<Chromatogram>

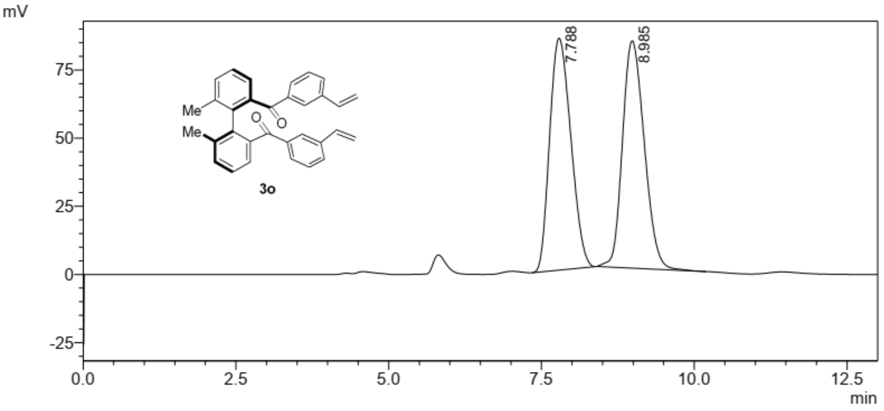

<Peak Table>

Detector A Channel 1 254nm

Peak#	Ret. Time	Area	Height	Conc.	Unit	Mark	Name
1	7.788	2050502	85008	49.733			
2	8.985	2072516	83369	50.267			
Total		4123018	168377				

<Chromatogram>

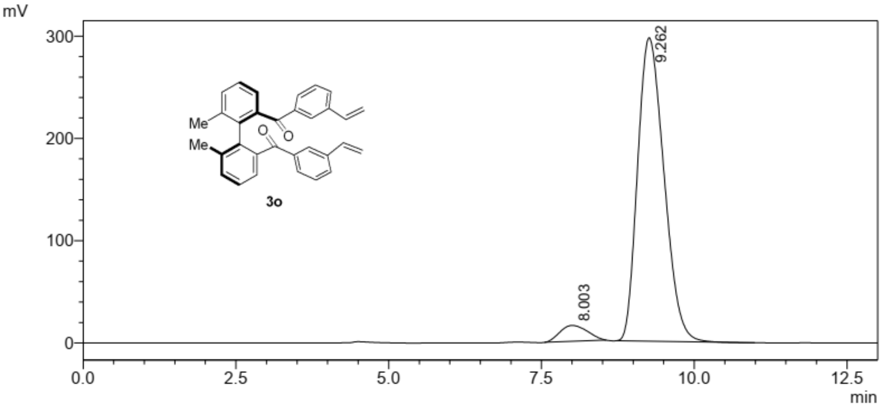

<Peak Table>

Detector A Channel 1 254nm

Peak#	Ret. Time	Area	Height	Conc.	Unit	Mark	Name
1	8.003	488553	15566	5.195			
2	9.262	8916071	296618	94.805			
Total		9404624	312184				

<Chromatogram>

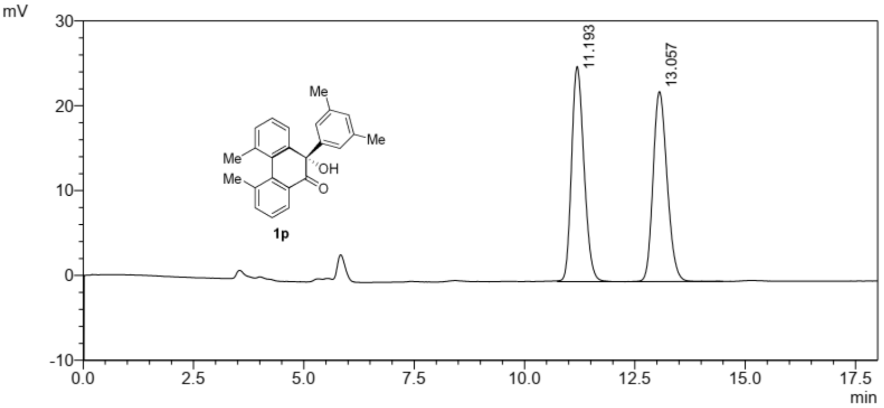

<Peak Table>

Detector A Channel 1 254nm

Peak#	Ret. Time	Area	Height	Conc.	Unit	Mark	Name
1	11.193	498008	25348	50.120			
2	13.057	495628	22405	49.880			
Total		993636	47753				

<Chromatogram>

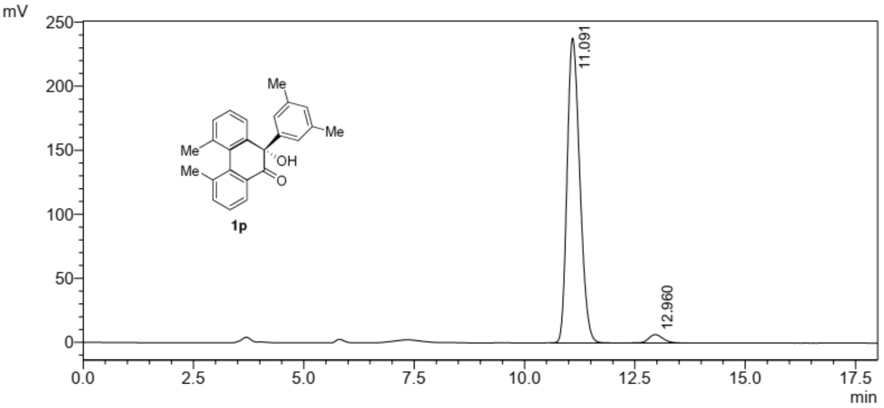

<Peak Table>

Detector A Channel 1 254nm

Peak#	Ret. Time	Area	Height	Conc.	Unit	Mark	Name
1	11.091	4747802	238031	97.010			
2	12.960	146346	6530	2.990			
Total		4894148	244561				

<Chromatogram>

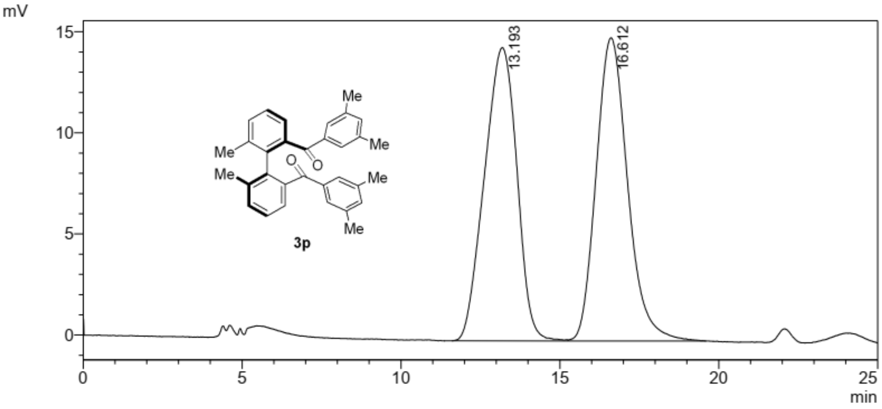

<Peak Table>

Detector A Channel 1 254nm

Peak#	Ret. Time	Area	Height	Conc.	Unit	Mark	Name
1	13.193	1032111	14507	50.001			
2	16.612	1032078	14994	49.999			
Total		2064189	29501				

<Chromatogram>

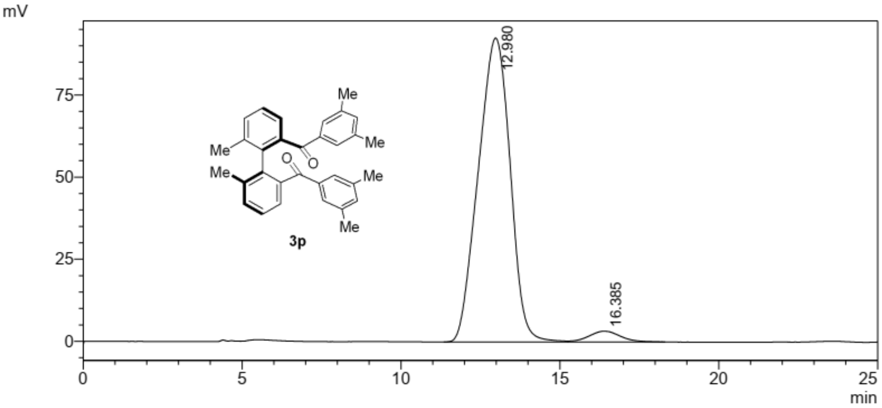

<Peak Table>

Detector A Channel 1 254nm

Peak#	Ret. Time	Area	Height	Conc.	Unit	Mark	Name
1	12.980	6457181	92567	96.571			
2	16.385	229249	3305	3.429			
Total		6686430	95872				

<Chromatogram>

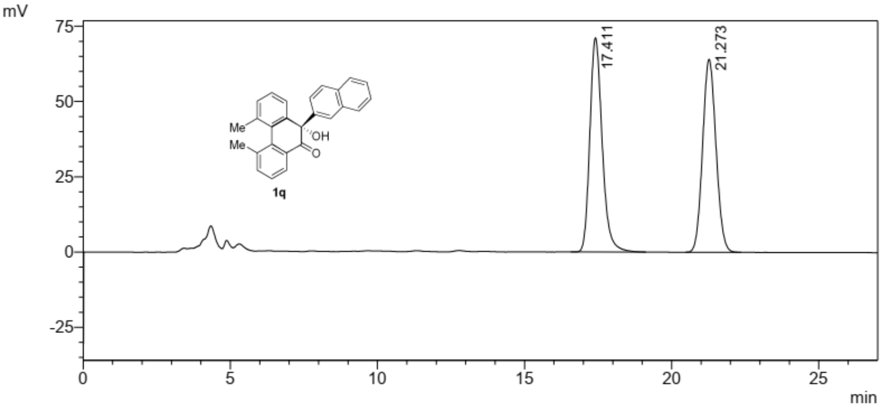

<Peak Table>

Detector A Channel 1 254nm

Peak#	Ret. Time	Area	Height	Conc.	Unit	Mark	Name
1	17.411	2076907	71123	50.563			
2	21.273	2030681	64135	49.437			
Total		4107588	135259				

<Chromatogram>

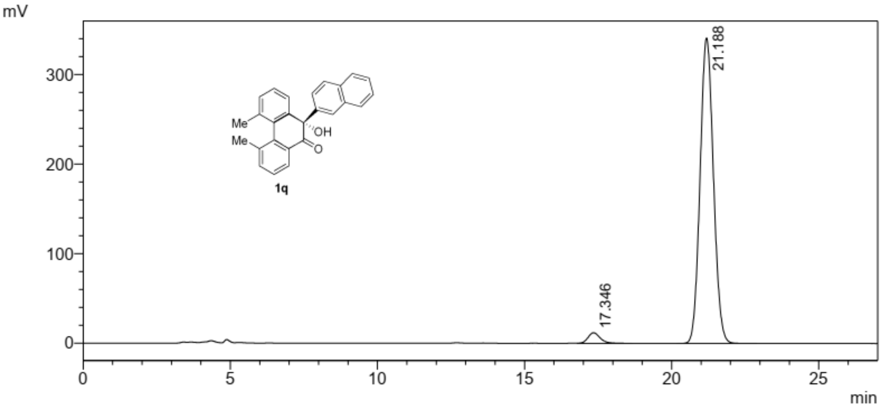

<Peak Table>

Detector A Channel 1 254nm

Peak#	Ret. Time	Area	Height	Conc.	Unit	Mark	Name
1	17.346	342467	11827	3.086			
2	21.188	10753770	340887	96.914			
Total		11096237	352715				

<Chromatogram>

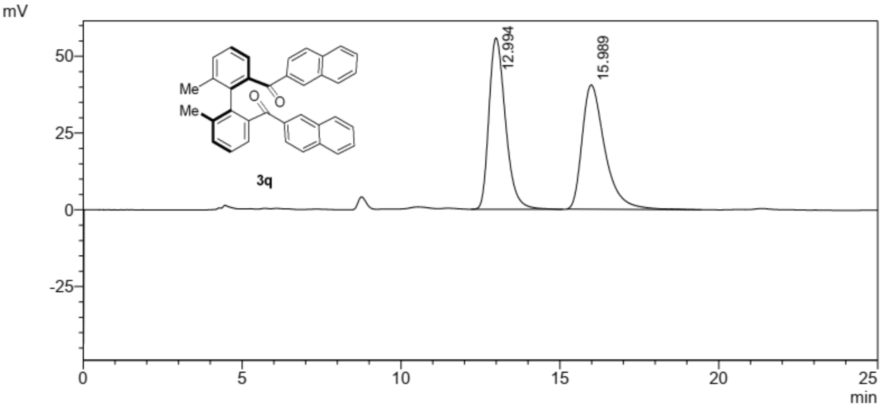

<Peak Table>

Detector A Channel 1 254nm

Peak#	Ret. Time	Area	Height	Conc.	Unit	Mark	Name
1	12.994	2022663	55787	50.102			
2	15.989	2014432	40458	49.898			
Total		4037094	96245				

<Chromatogram>

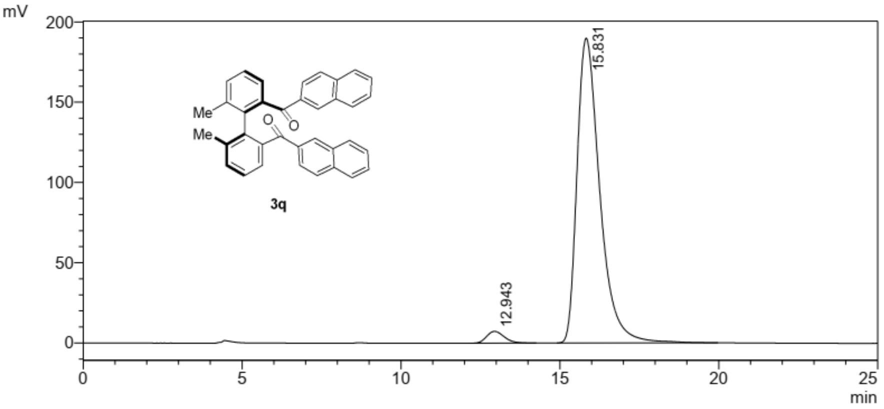

<Peak Table>

Detector A Channel 1 254nm

Peak#	Ret. Time	Area	Height	Conc.	Unit	Mark	Name
1	12.943	281601	7316	2.888			
2	15.831	9469953	189857	97.112			
Total		9751554	197174				

<Chromatogram>

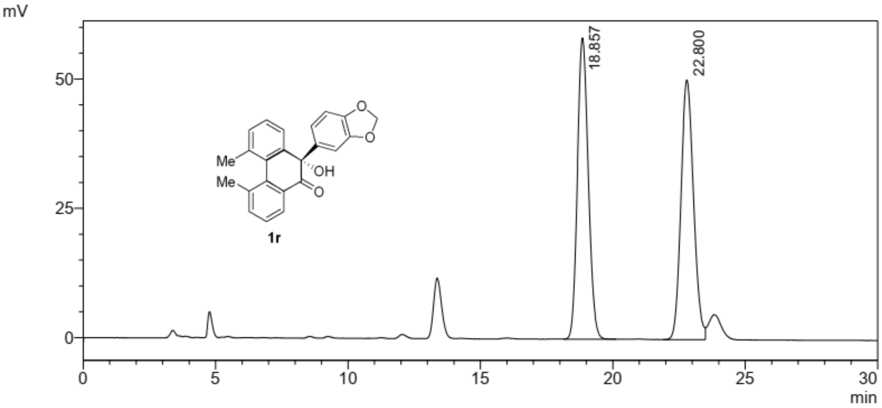

<Peak Table>

Detector A Channel 1 254nm

Peak#	Ret. Time	Area	Height	Conc.	Unit	Mark	Name
1	18.857	1659765	58248	50.000			
2	22.800	1659765	50209	50.000			
Total		3319530	108457				

<Chromatogram>

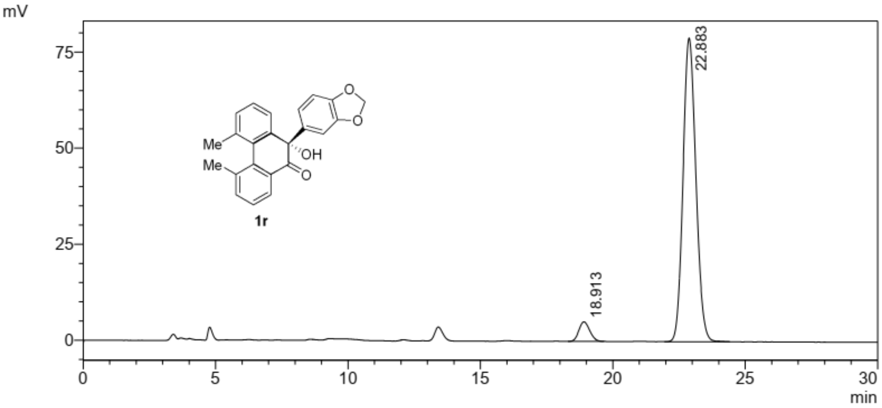

<Peak Table>

Detector A Channel 1 254nm

Peak#	Ret. Time	Area	Height	Conc.	Unit	Mark	Name
1	18.913	145520	5095	5.184			
2	22.883	2661513	79027	94.816			
Total		2807033	84122				

<Chromatogram>

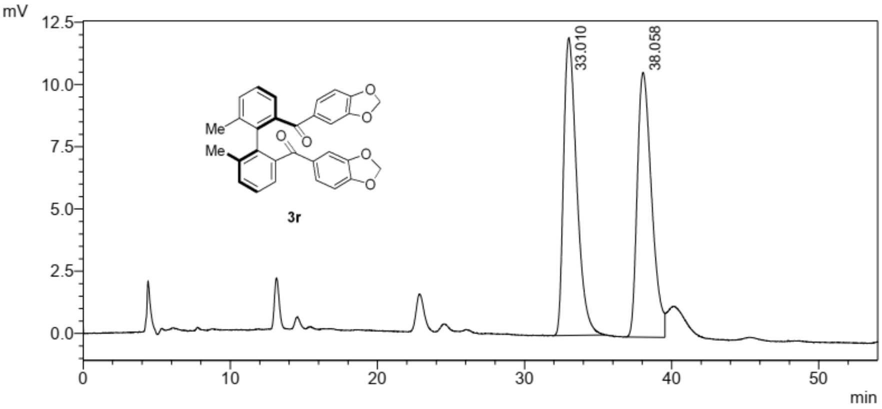

<Peak Table>

Detector A Channel 1 254nm

Peak#	Ret. Time	Area	Height	Conc.	Unit	Mark	Name
1	33.010	743404	11950	50.719			
2	38.058	722321	10633	49.281			
Total		1465725	22583				

<Chromatogram>

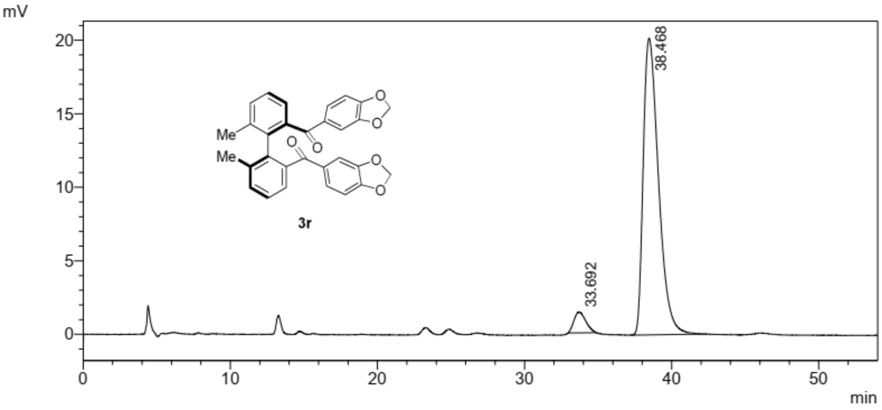

<Peak Table>

Detector A Channel 1 254nm

Peak#	Ret. Time	Area	Height	Conc.	Unit	Mark	Name
1	33.692	78825	1421	5.139			
2	38.468	1454901	20172	94.861			
Total		1533726	21594				

<Chromatogram>

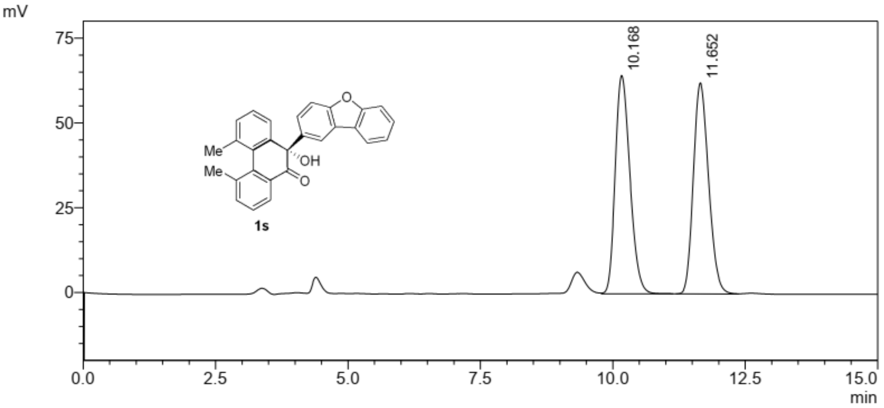

<Peak Table>

Detector A Channel 1 254nm

Peak#	Ret. Time	Area	Height	Conc.	Unit	Mark	Name
1	10.168	1243517	64351	49.954			
2	11.652	1245798	62216	50.046			
Total		2489315	126566				

<Chromatogram>

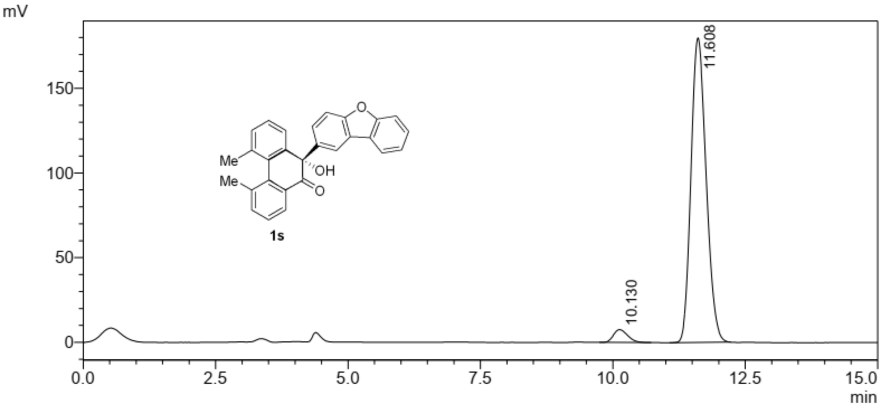

<Peak Table>

Detector A Channel 1 254nm

Peak#	Ret. Time	Area	Height	Conc.	Unit	Mark	Name
1	10.130	141300	7556	3.795			
2	11.608	3581819	179758	96.205			
Total		3723119	187314				

<Chromatogram>

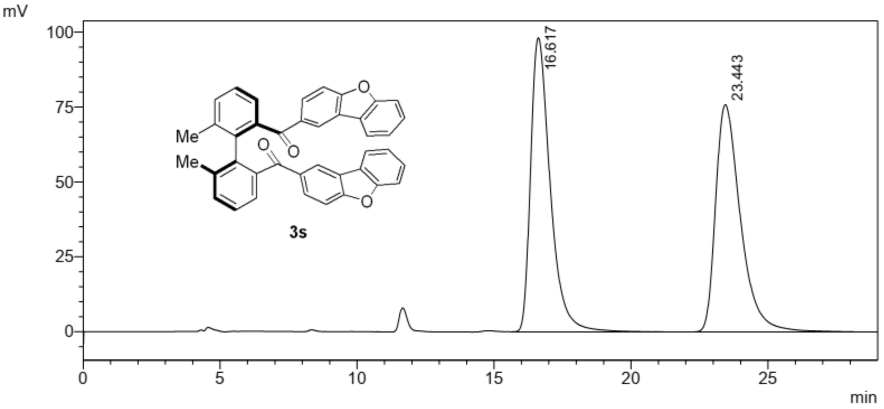

<Peak Table>

Detector A Channel 1 254nm

Peak#	Ret. Time	Area	Height	Conc.	Unit	Mark	Name
1	16.617	4894659	98164	50.446			
2	23.443	4808110	75849	49.554			
Total		9702768	174014				

<Chromatogram>

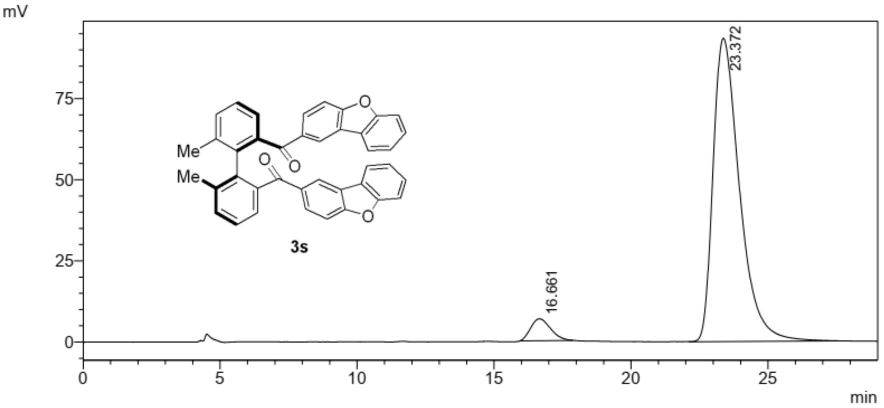

<Peak Table>

Detector A Channel 1 254nm

Peak#	Ret. Time	Area	Height	Conc.	Unit	Mark	Name
1	16.661	338502	6718	5.115			
2	23.372	6279906	93419	94.885			
Total		6618408	100137				

<Chromatogram>

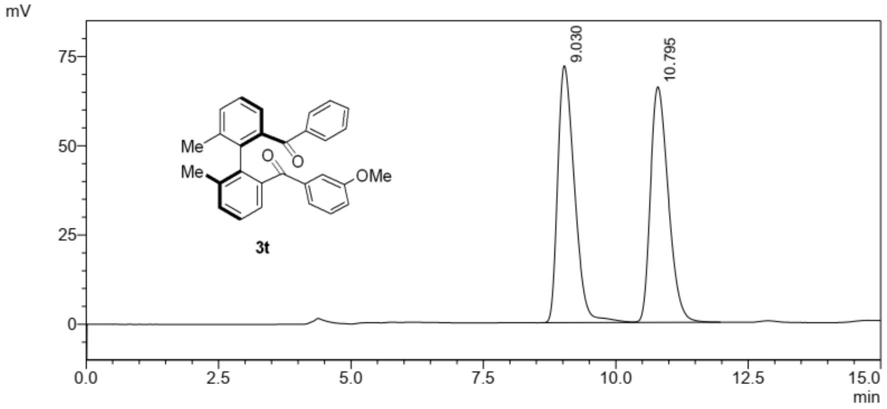

<Peak Table>

Detector A Channel 1 254nm

Peak#	Ret. Time	Area	Height	Conc.	Unit	Mark	Name
1	9.030	1592299	71972	50.694			
2	10.795	1548672	65973	49.306			
Total		3140971	137945				

<Chromatogram>

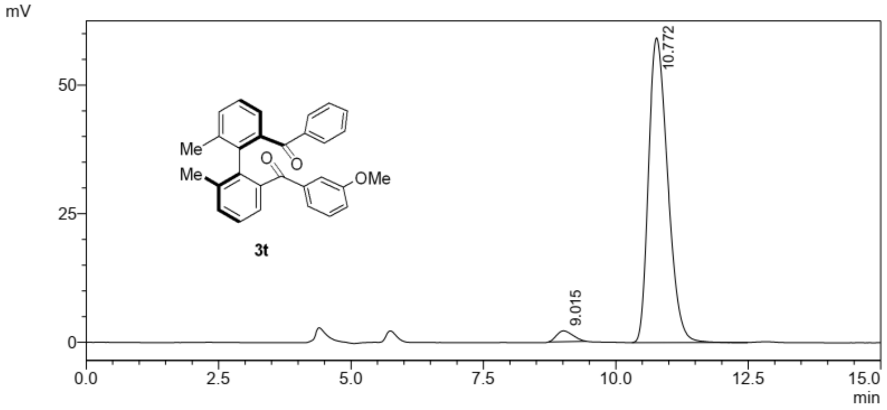

<Peak Table>

Detector A Channel 1 254nm

Peak#	Ret. Time	Area	Height	Conc.	Unit	Mark	Name
1	9.015	44584	2098	2.906			
2	10.772	1489785	59210	97.094			
Total		1534370	61308				

<Chromatogram>

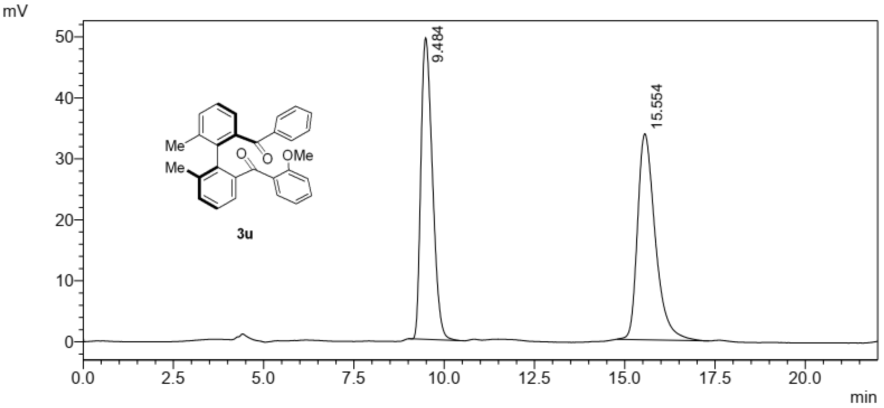

<Peak Table>

Detector A Channel 1 254nm

Peak#	Ret. Time	Area	Height	Conc.	Unit	Mark	Name
1	9.484	1097094	49441	48.704			
2	15.554	1155486	33767	51.296			
Total		2252580	83208				

<Chromatogram>

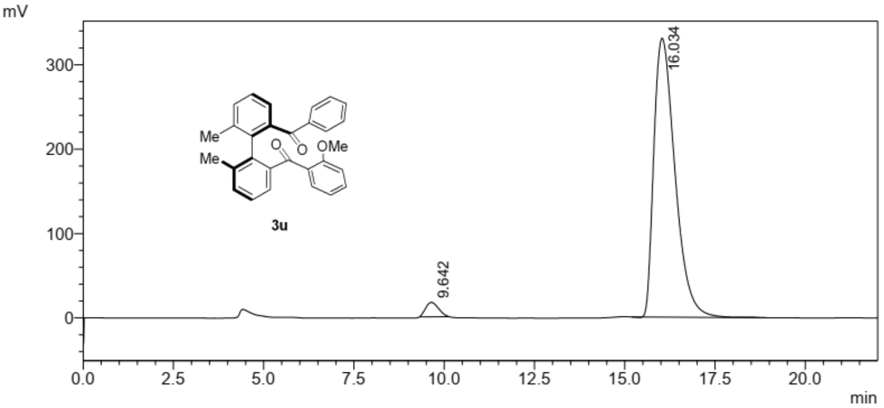

<Peak Table>

Detector A Channel 1 254nm

Peak#	Ret. Time	Area	Height	Conc.	Unit	Mark	Name
1	9.642	421988	17027	3.032			
2	16.034	13493722	330655	96.968			
Total		13915710	347682				

<Chromatogram>

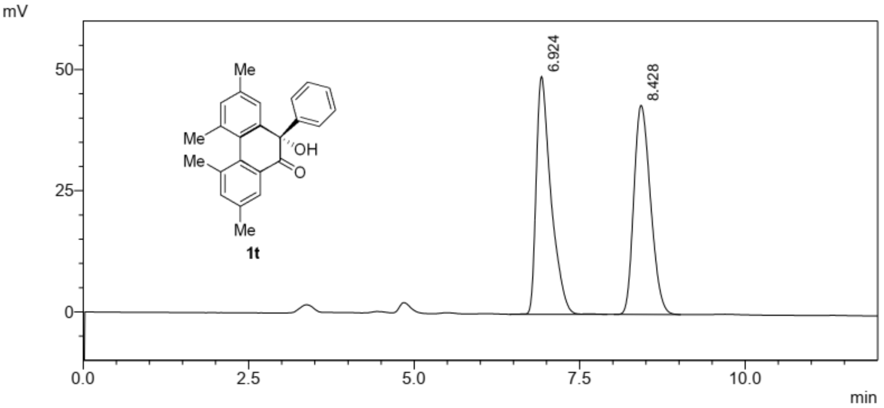

<Peak Table>

Detector A Channel 1 254nm

Peak#	Ret. Time	Area	Height	Conc.	Unit	Mark	Name
1	6.924	758957	49057	50.052			
2	8.428	757367	43132	49.948			
Total		1516323	92189				

<Chromatogram>

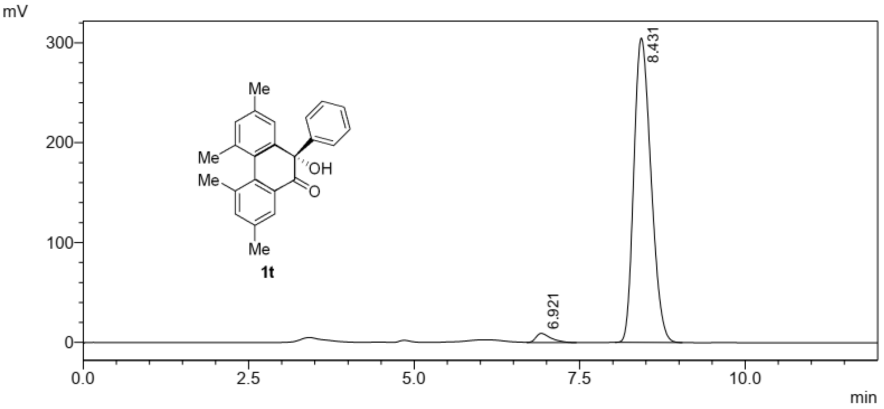

<Peak Table>

Detector A Channel 1 254nm

Peak#	Ret. Time	Area	Height	Conc.	Unit	Mark	Name
1	6.921	144859	9292	2.571			
2	8.431	5488625	304570	97.429			
Total		5633484	313862				

<Chromatogram>

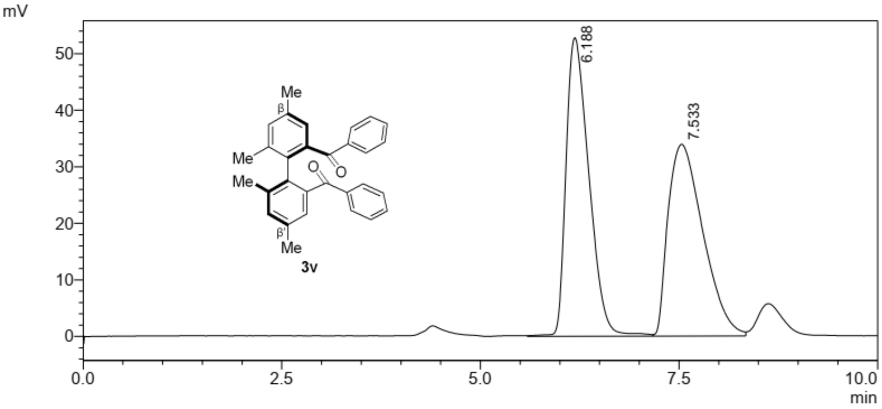

<Peak Table>

Detector A Channel 1 254nm

Peak#	Ret. Time	Area	Height	Conc.	Unit	Mark	Name
1	6.188	1057723	52753	50.505			
2	7.533	1036572	33919	49.495			
Total		2094295	86672				

<Chromatogram>

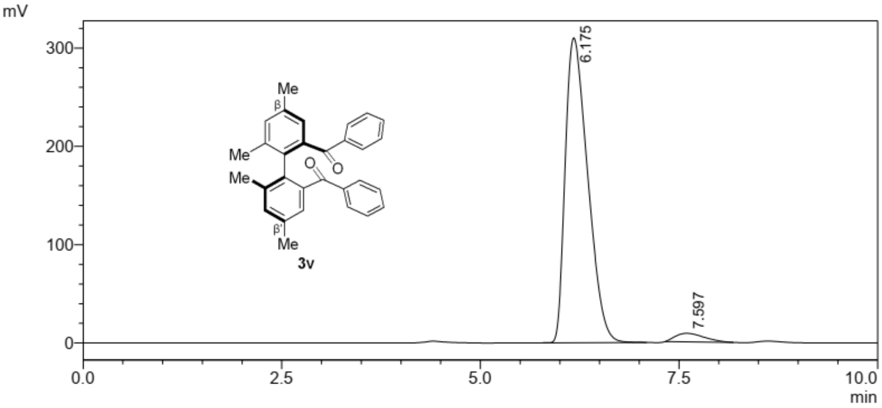

<Peak Table>

Detector A Channel 1 254nm

Peak#	Ret. Time	Area	Height	Conc.	Unit	Mark	Name
1	6.175	6100256	310008	96.528			
2	7.597	219444	8684	3.472			
Total		6319700	318692				

<Chromatogram>

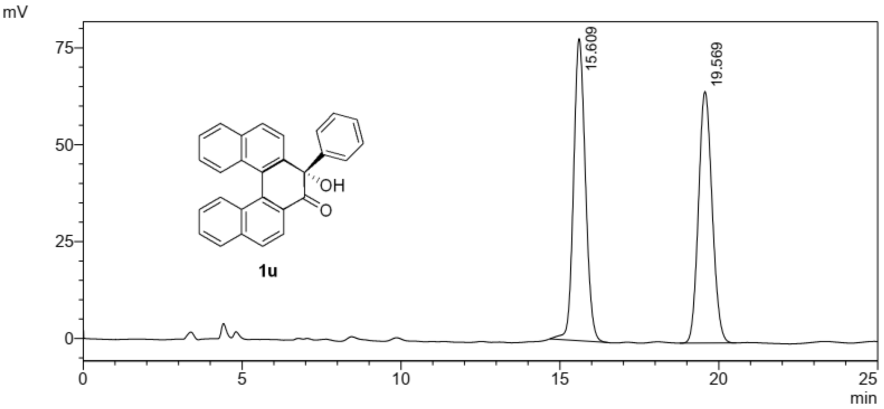

<Peak Table>

Detector A Channel 1 254nm

Peak#	Ret. Time	Area	Height	Conc.	Unit	Mark	Name
1	15.609	2012461	77908	50.903			
2	19.569	1941074	64915	49.097			
Total		3953535	142823				

<Chromatogram>

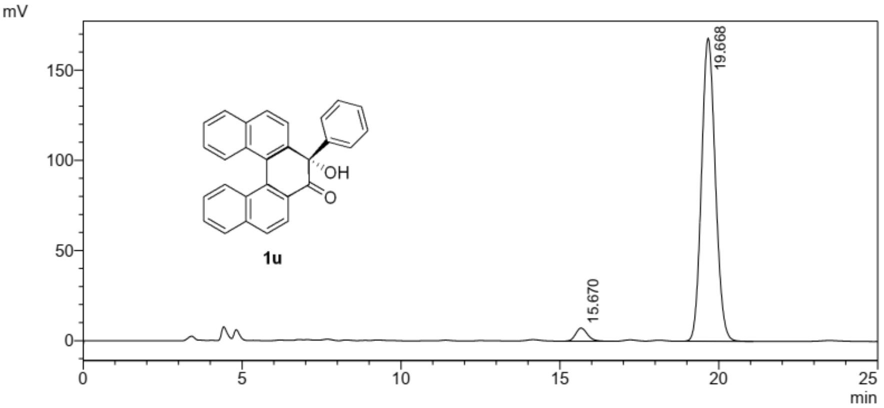

<Peak Table>

Detector A Channel 1 254nm

Peak#	Ret. Time	Area	Height	Conc.	Unit	Mark	Name
1	15.670	187318	7266	3.549			
2	19.668	5090215	168053	96.451			
Total		5277534	175319				

<Chromatogram>

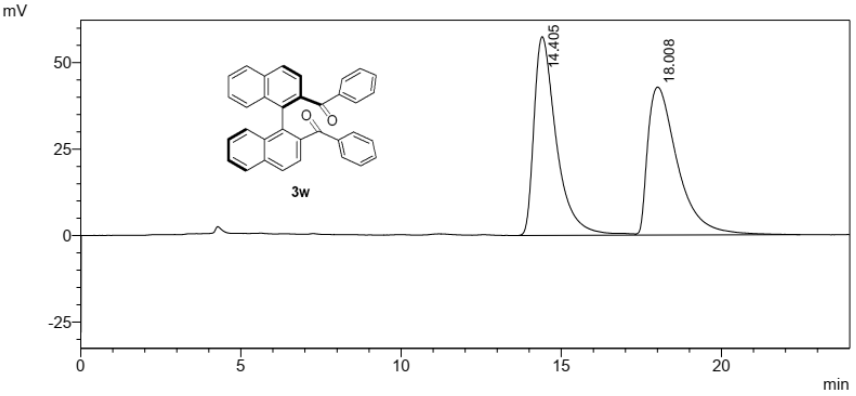

<Peak Table>

Detector A Channel 1 254nm

Peak#	Ret. Time	Area	Height	Conc.	Unit	Mark	Name
1	14.405	2798198	57473	50.285			
2	18.008	2766520	42779	49.715			
Total		5564718	100251				

<Chromatogram>

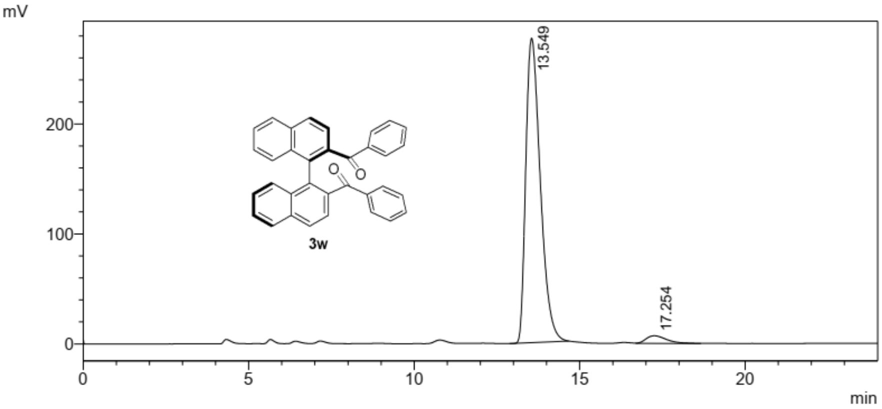

<Peak Table>

Detector A Channel 1 254nm

Peak#	Ret. Time	Area	Height	Conc.	Unit	Mark	Name
1	13.549	8440868	276718	96.554			
2	17.254	301238	6944	3.446			
Total		8742106	283662				

<Chromatogram>

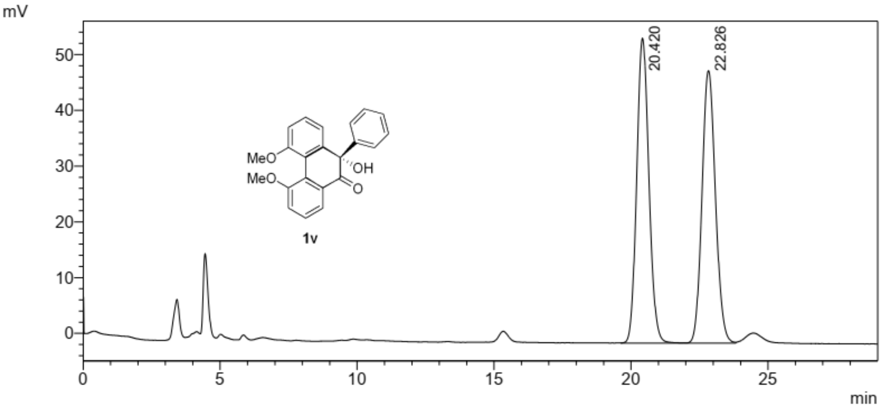

<Peak Table>

Detector A Channel 1 254nm

Peak#	Ret. Time	Area	Height	Conc.	Unit	Mark	Name
1	20.420	1691778	54666	50.414			
2	22.826	1663972	48848	49.586			
Total		3355750	103514				

<Chromatogram>

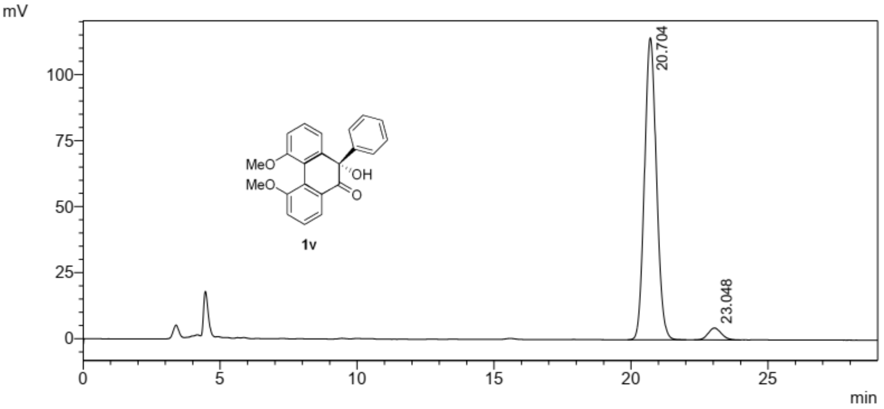

<Peak Table>

Detector A Channel 1 254nm

Peak#	Ret. Time	Area	Height	Conc.	Unit	Mark	Name
1	20.704	3469505	114320	95.850			
2	23.048	150223	4487	4.150			
Total		3619728	118807				

<Chromatogram>

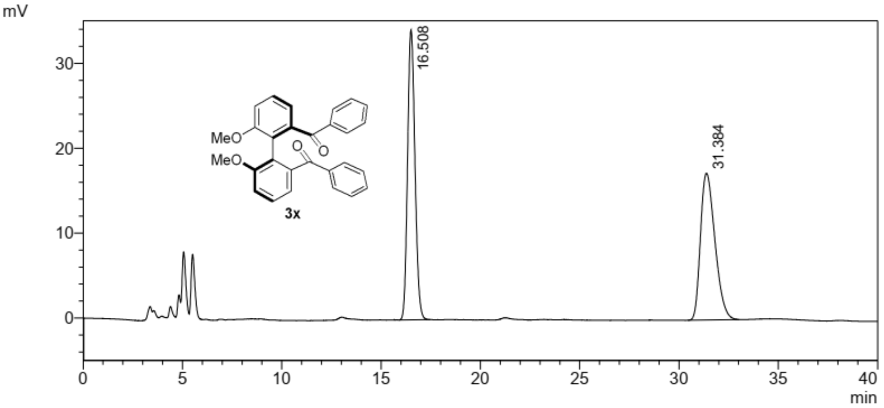

<Peak Table>

Detector A Channel 1 210nm

Peak#	Ret. Time	Area	Height	Conc.	Unit	Mark	Name
1	16.508	880851	34137	50.152			
2	31.384	875509	17294	49.848			
Total		1756360	51431				

<Chromatogram>

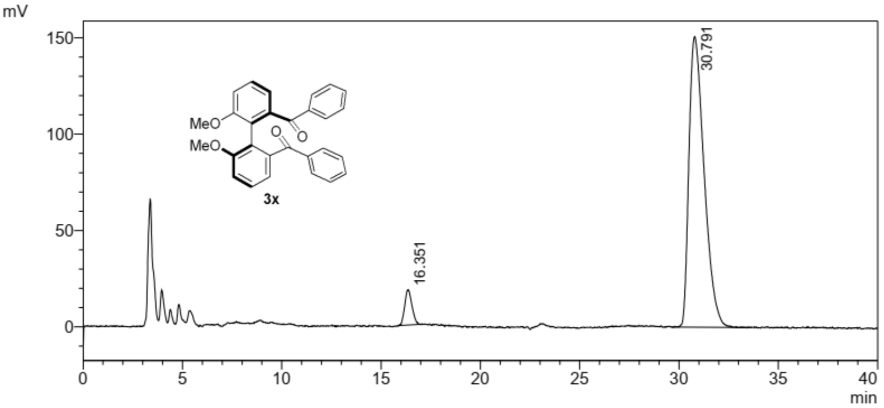

<Peak Table>

Detector A Channel 2 210nm

Peak#	Ret. Time	Area	Height	Conc.	Unit	Mark	Name
1	16.351	464575	18195	5.474			
2	30.791	8022900	150817	94.526			
Total		8487475	169013				

<Chromatogram>

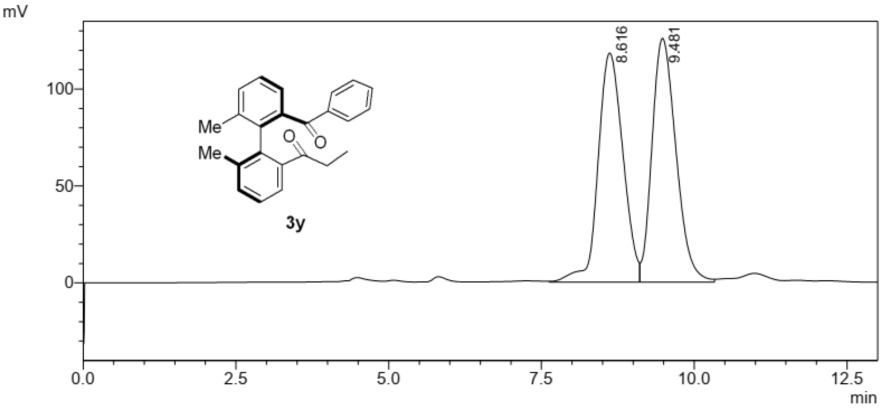

<Peak Table>

Detector A Channel 1 254nm

Peak#	Ret. Time	Area	Height	Conc.	Unit	Mark	Name
1	8.616	3348777	118261	49.331			
2	9.481	3439577	125829	50.669			
Total		6788354	244090				

<Chromatogram>

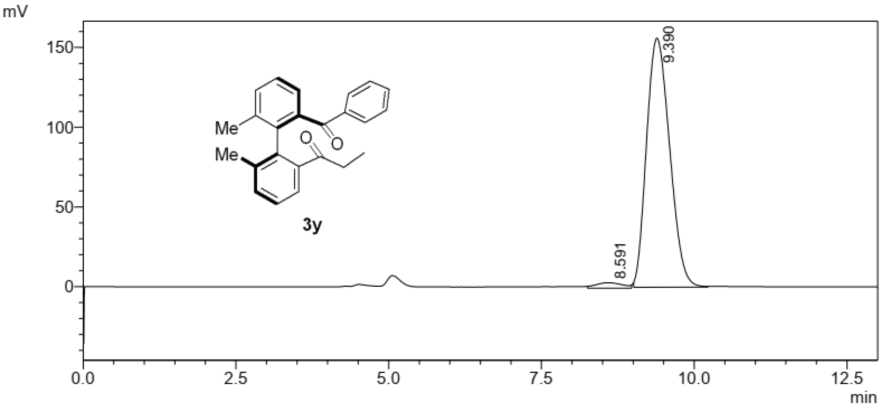

<Peak Table>

Detector A Channel 1 254nm

Peak#	Ret. Time	Area	Height	Conc.	Unit	Mark	Name
1	8.591	107068	3442	2.537			
2	9.390	4112820	156161	97.463			
Total		4219888	159603				

<Chromatogram>

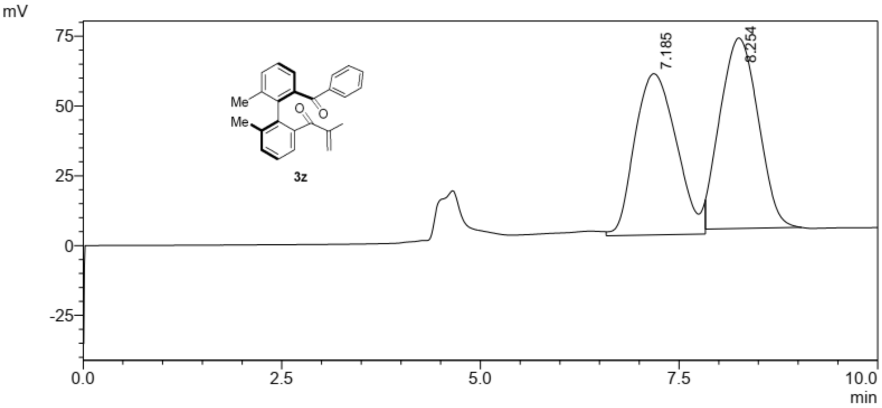

<Peak Table>

Detector A Channel 1 254nm

Peak#	Ret. Time	Area	Height	Conc.	Unit	Mark	Name
1	7.185	2120854	57778	48.296			
2	8.254	2270535	68199	51.704			
Total		4391390	125977				

<Chromatogram>

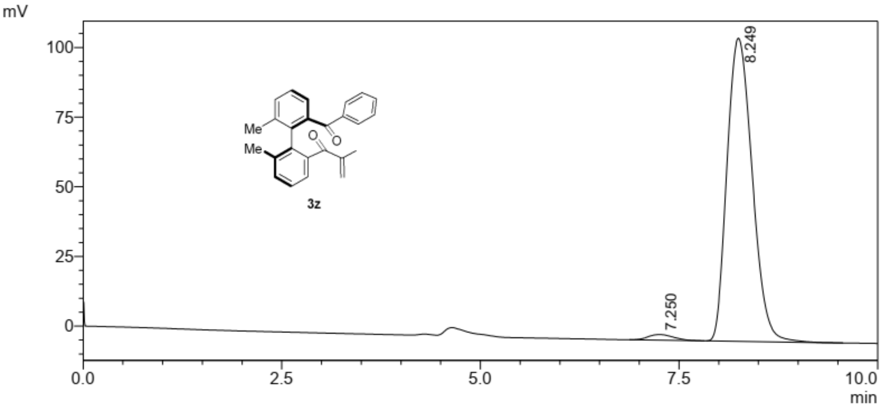

<Peak Table>

Detector A Channel 1 254nm

Peak#	Ret. Time	Area	Height	Conc.	Unit	Mark	Name
1	7.250	44587	2013	1.822			
2	8.249	2403005	108789	98.178			
Total		2447592	110802				

<Chromatogram>

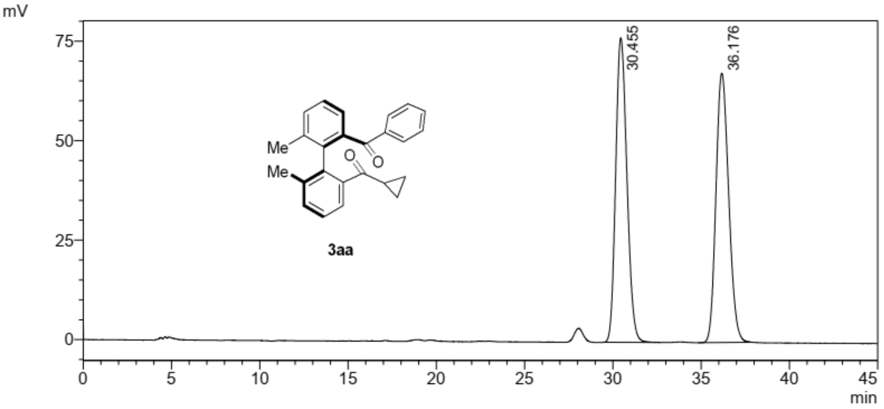

<Peak Table>

Detector A Channel 1 254nm

Peak#	Ret. Time	Area	Height	Conc.	Unit	Mark	Name
1	30.455	3385735	76495	49.984			
2	36.176	3387913	67677	50.016			
Total		6773648	144172				

<Chromatogram>

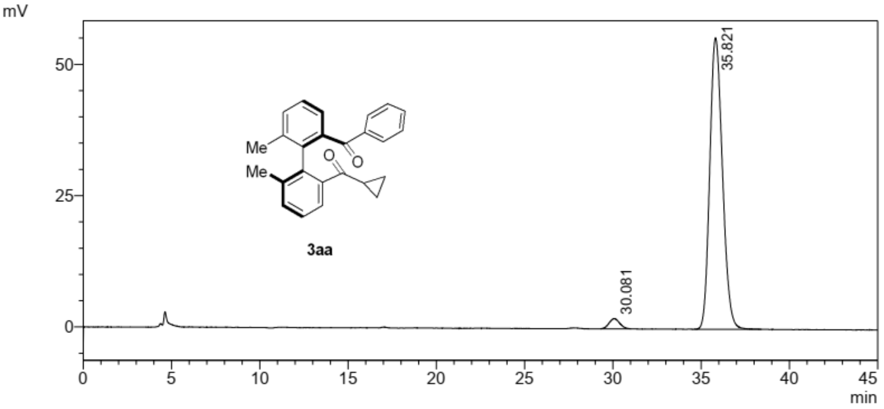

<Peak Table>

Detector A Channel 1 254nm

Peak#	Ret. Time	Area	Height	Conc.	Unit	Mark	Name
1	30.081	81664	1947	2.858			
2	35.821	2776071	55487	97.142			
Total		2857735	57434				

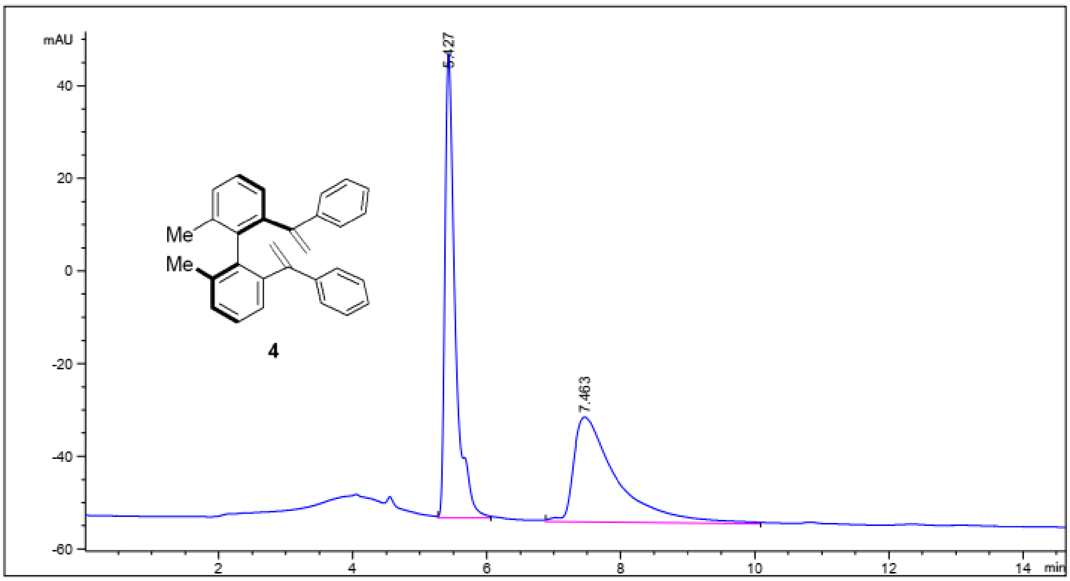

Signal 1: DAD1 A, Sig=254,4 Ref=360,100

Peak #	RetTime [min]	Type	Width [min]	Area [mAU*s]	Height [mAU]	Area %
1	5.427	MM	0.1776	1065.67090	100.02626	51.1863
2	7.463	MM	0.7457	1016.27515	22.71397	48.8137

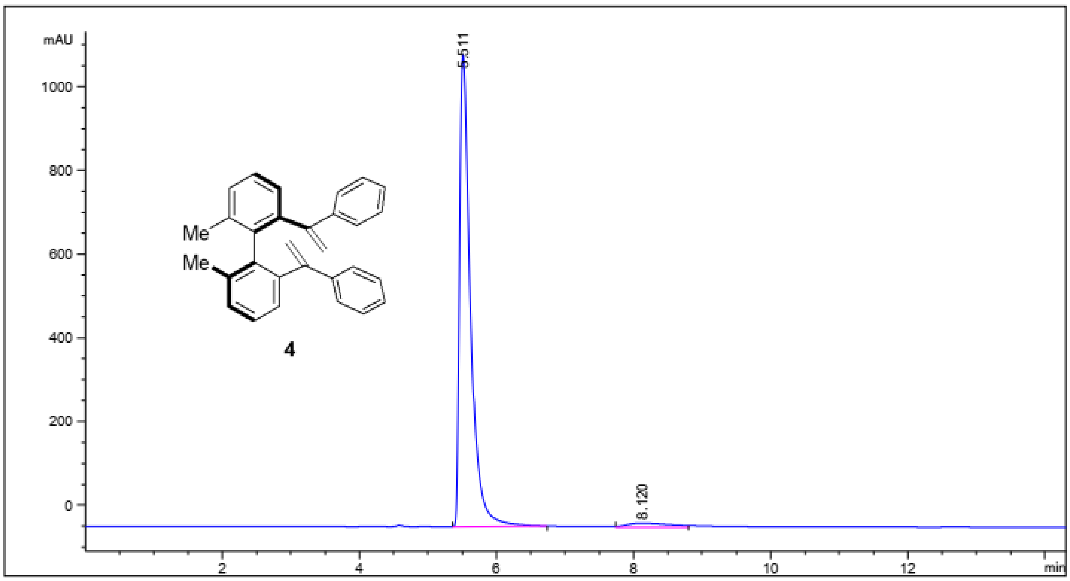

Signal 1: DAD1 A, Sig=254,4 Ref=360,100

Peak #	RetTime [min]	Type	Width [min]	Area [mAU*s]	Height [mAU]	Area %
1	5.511	BB	0.1684	1.26104e4	1128.08789	96.5156
2	8.120	MM	0.7375	455.25446	10.28883	3.4844

<Chromatogram>

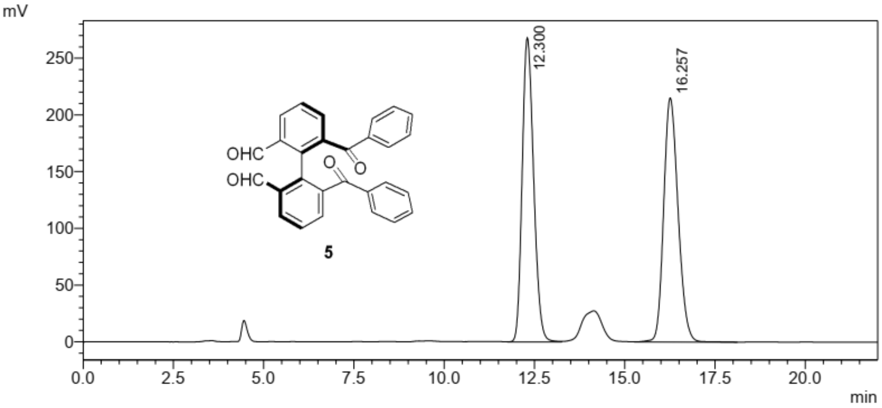

<Peak Table>

Detector A Channel 1 254nm

Peak#	Ret. Time	Area	Height	Conc.	Unit	Mark	Name
1	12.300	5920567	268079	49.761			
2	16.257	5977374	215171	50.239			
Total		11897940	483250				

<Chromatogram>

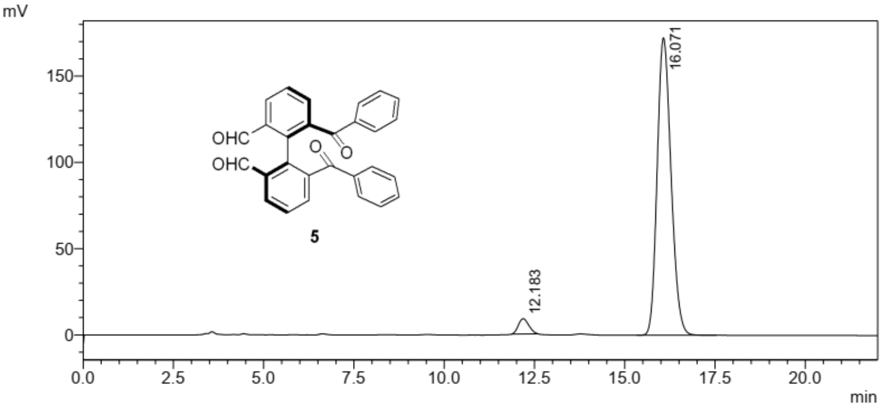

<Peak Table>

Detector A Channel 1 254nm

Peak#	Ret. Time	Area	Height	Conc.	Unit	Mark	Name
1	12.183	179033	8870	3.740			
2	16.071	4607472	172376	96.260			
Total		4786505	181246				

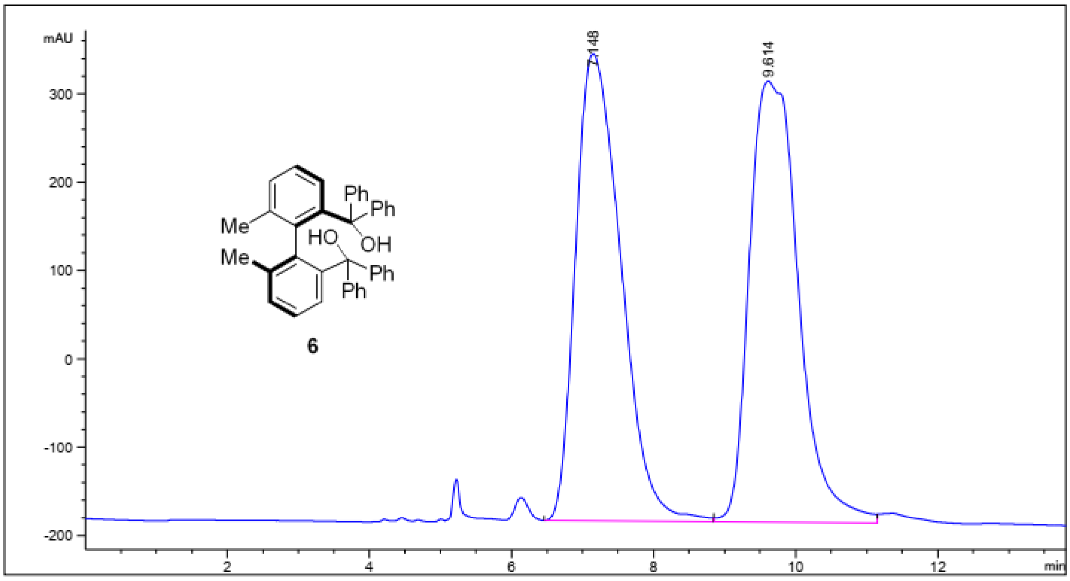

Signal 1: DAD1 C, Sig=230,4 Ref=360,100

Peak #	RetTime [min]	Type	Width [min]	Area [mAU*s]	Height [mAU]	Area %
1	7.148	VB	0.7214	2.34489e4	528.77924	49.3565
2	9.614	BB	0.6736	2.40604e4	499.83356	50.6435

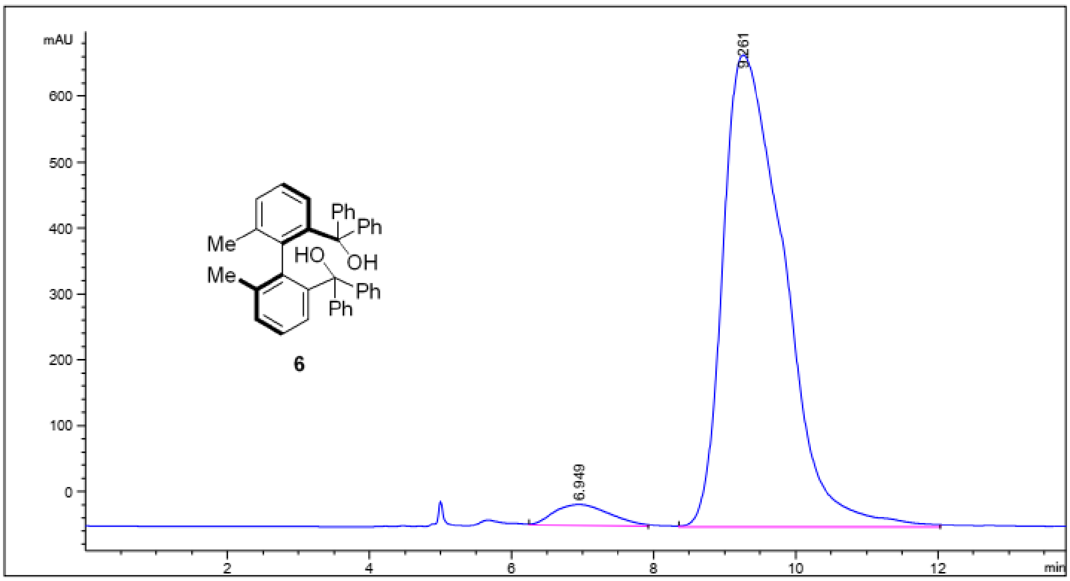

Signal 1: DAD1 D, Sig=230,4 Ref=360,100

Peak #	RetTime [min]	Type	Width [min]	Area [mAU*s]	Height [mAU]	Area %
1	6.949	MM	0.9096	1745.80261	31.98970	3.9179
2	9.261	MM	0.9957	4.28138e4	716.62024	96.0821
